# Lack of ADAP1/Centaurin-α1 Ameliorates Cognitive Impairment and Neuropathological Hallmarks in a Mouse Model of Alzheimer's Disease

**DOI:** 10.1523/ENEURO.0063-25.2025

**Published:** 2025-11-21

**Authors:** Erzsebet M. Szatmari, Corey Moran, Sarah J. Cohen, Denys Bashtovyy, Amanda Jacob, Wyatt Bunner, Mary Phipps, Joan Cristino Lora, Robert W. Stackman, Ryohei Yasuda

**Affiliations:** ^1^Max Planck Florida Institute for Neuroscience, Jupiter, Florida 33458; ^2^Department of Physical Therapy, East Carolina University, Greenville, North Carolina 27834; ^3^Florida Atlantic University, John D. McArthur Campus, Jupiter, Florida 33458

**Keywords:** aging, Alzheimer's disease, dendritic spines, neuroinflammation, neuronal signaling, transcriptome

## Abstract

ArfGAP, with dual PH domain-containing protein 1/Centaurin-α1 (ADAP1/CentA1), is a brain-enriched and highly conserved Arf6 GTPase-activating and Ras-anchoring protein. CentA1 is involved in dendritic outgrowth and arborization, synaptogenesis, and axonal polarization by regulating the actin cytoskeleton dynamics. CentA1 upregulation and association with amyloid plaques in the human Alzheimer's disease (AD) brain suggest the role of this protein in AD progression. To understand the role of CentA1 in neurodegeneration, we crossbred CentA1 knock-out (KO) mice with the J20 mouse model of AD. We evaluated AD-associated behavioral and neuropathological hallmarks and gene expression profiles in J20 and J20 crossed with CentA1 KO (J20xKO) male mice to determine the impact of eliminating CentA1 expression on AD-related phenotypes. Spatial memory assessed by the Morris water maze test showed significant impairment in J20 mice, which was rescued in J20xKO mice. Moreover, neuropathological hallmarks of AD, such as amyloid plaque deposits and neuroinflammation, were significantly reduced in J20xKO mice. To identify potential mediators of AD phenotype rescue, we analyzed differentially expressed genes between genotypes. We found that changes in the gene profile by deletion of CentA1 from J20 (J20xKO vs J20) were anticorrelated with changes caused by APP overexpression (J20 vs wild type), consistent with rescue of J20 phenotypes by CentA1 KO. In summary, our data indicate that CentA1 is required for the progression of AD phenotypes in this model and that targeting CentA1 signaling might have therapeutic potential for AD prevention or treatment.

## Significance Statement

ADAP1/Centaurin-α1 (CentA1) is highly enriched in the brain, and an increased CentA1 level has been linked to Alzheimer's disease (AD). However, the precise role of CentA1 in the pathogenesis of AD is poorly understood. We found that genetic deletion of CentA1 in the AD model mice rescues the pathological hallmarks of AD, including loss of dendritic spines in the hippocampus, amyloid plaque deposition, neuroinflammation, and spatial memory deficits. Transcriptome analysis of the forebrain demonstrated that gene expression changes caused by APP overexpression were restored in J20 mice lacking CentA1. These findings support the role of CentA1 in AD progression.

## Introduction

Alzheimer's disease (AD) is a pervasive neurodegenerative condition characterized by progressive decline of memory and cognitive function (McKhann et al., 2011; Dawson et al., 2018; van der Flier et al., 2023). Extracellular amyloid plaque depositions, formation of intracellular neurofibrillary tangles, widespread neuroinflammation, neuronal cell death, and alterations in brain morphology are the major pathologies of AD (De Strooper and Karran, 2016; Long and Holtzman, 2019; Manno et al., 2019). However, the molecular complexity underlying these pathological transformations is poorly understood. Early in AD, soluble Aβ oligomer-induced loss of dendritic spines, aberrant synaptic remodeling, and abnormal synaptic transmission cause deficits in the activity of the networks mediating cognitive functions and learning and memory (Jacobsen et al., 2006; Wei et al., 2010; Li et al., 2011; Kommaddi et al., 2018). Several molecular signaling mechanisms have been implicated in the Aβ-triggered synaptic dysfunction, including eIK2α kinases, NMDA receptor signaling, the Ras-ERK pathway, CAMKK2-AMPK kinase pathway, GSK3β, and the Centaurin-α1 (CentA1)-Ras-Elk1 signaling at mitochondria (Ma et al., 2013; Mairet-Coello et al., 2013; Szatmari et al., 2013; Sinnen et al., 2016; Kirouac et al., 2017). CentA1 is highly expressed in brain areas involved in AD, including the hippocampus (Hammonds-Odie et al., 1996; Sedehizade et al., 2002; Aggensteiner and Reiser, 2003). In neurons, CentA1 is expressed in the axon (Kreutz et al., 1997; Stricker et al., 1997), dendrites, dendritic spines, and the nucleus (Yoshimura et al., 2004; Moore et al., 2007), and it is localized to the plasma membrane and the mitochondria (Stricker and Reiser, 2014).

Structurally, CentA1 is a multidomain protein with an ArfGAP domain, which targets Arf6, and two PH domains (Thacker et al., 2004; Venkateswarlu et al., 2005). CentA1 interacts with several signaling molecules, including PI3K; cytoskeletal, nuclear, and mitochondrial proteins; and cytosolic kinases, providing a scaffolding platform for these proteins (Stricker and Reiser, 2014). Additionally, CentA1 interacts with Ras and facilitates the activation of the Ras-ERK1/2 pathway (Hayashi et al., 2006; Szatmari et al., 2013).

Several studies suggested that CentA1 is involved in AD progression. In the postmortem human AD brain, the level of CentA1 increases, particularly around neuritic plaques (Reiser and Bernstein, 2002, 2004; Stricker and Reiser, 2014). Using cellular models of AD, it was previously reported that in cultured neurons and hippocampal slices, Aβ-induced dendritic spine loss and aberrant spine structural plasticity are significantly suppressed if CentA1 is downregulated via shRNA (Szatmari et al., 2013). In addition, CentA1 knock-out (KO) mice exhibited enhanced dendritic spine density and structural LTP in the hippocampus and improved performance in a hippocampus-dependent spatial memory task (Szatmari et al., 2021).

In this study, to verify the involvement of CentA1 in the molecular pathogenesis of AD, we evaluated AD-related phenotypes in the J20 mouse model of AD, in which CentA1 is knocked out (J20xKO). The J20 mouse model overexpresses human APP (hAPP) with Swedish and Indiana mutations. These mice simulate several hallmarks of sporadic and autosomal dominant AD, including neuroinflammation, cerebral Aβ plaque burden, synaptic dysfunction, spontaneous epileptic activity, and deficits in spatial memory and learning (Mucke et al., 2000; Verret et al., 2012; Suberbielle et al., 2013; Wright et al., 2013). Using histological analysis, biochemical studies, and behavioral assay, we found that the removal of CentA1 rescues many of the pathological hallmarks of J20, including dendritic spine elimination, amyloid plaque deposition, inflammatory elevation, and learning impairment. Differentially expressed gene (DEG) analysis indicated that CentA1 KO restores the expression of many genes abnormally regulated in J20 animals. Thus, we conclude that CentA1 signaling represents a promising therapeutic target for treating AD.

## Materials and Methods

### Animals

All mice were housed in an Animal Resource Facility compliant with the US National Institutes of Health Guide for Care and Use of Laboratory Animals. CentA1 KO mice previously described (Szatmari et al., 2021) were crossbred with the J20 mouse model of AD. J20 mice (Mucke et al., 2000) were in-house bred by crossing heterozygous and wild-type (WT; C57BL/6) mice. J20 mice were heterozygous for transgene. Genotyping was performed before experiments using PCR of genomic DNA extracted from ear snip material (Transnetyx). At the end of the experiment, the genotype was reconfirmed by Western blotting of brain samples. Mice were group-housed (2–4 mice/cage), and littermates were randomly distributed over different cages. Nontransgenic littermates, referred to as WT mice, were used as controls. Only male mice were used for this study. The experimental groups were J20, J20 crossed with CentA1 KO (J20xKO); controls were WT littermates, ensuring that the mice were from the same parents, living in the same cage and uniformly affected by environmental factors.

### Immunofluorescence (IF) staining and imaging

We used a previously described method (Szatmari et al., 2021). Briefly, mice were deeply anesthetized with a ketamine/xylazine cocktail and then perfused transcardially with saline, followed by fixative [4% paraformaldehyde (PFA) in 0.1 M phosphate buffer (PB)] perfusion. Brains were postfixed at 4°C overnight. The 50 µm free-floating coronal sections were cut and collected in ice-cold 0.1 M PB, followed by incubation in blocking buffer (0.3% Triton X-100 and 0.5% normal goat serum in 0.1 M PB; 30 min). Samples were then incubated overnight with anti-NeuN antibody (ABN78, rabbit polyclonal, Millipore; 1:1,000 in blocking buffer). After a wash in 0.1 M PB, samples were incubated at room temperature for 2 h with Alexa Fluor 488-conjugated secondary antibodies (A-11008, Life Technologies) diluted 1:500 in blocking buffer. After a wash in 0.1 M PB, the nuclei were stained with Hoechst (1:10,000, H3570, Life Technologies) for 10 min. Sections were mounted with Fluoromount-G on Superfrost Plus slides (Thermo Fisher Scientific) and then imaged using a Zeiss LSM 710 confocal microscope. We imaged four sections from four mice for each condition (WT, J20, J20xKO). To ensure unbiased counting, we systemically sampled across each section, taking *z*-series every 150 µm for the dorsal CA1 hippocampus and 300 µm for the motor cortex, obtaining ∼12 images per section. Each *z*-series was imaged at a 20× magnification with a 0.23 × 0.23 µm pixel size at a 1 µm *z*-step for the hippocampus and 0.83 × 0.83 µm at a 1 µm *z*-step for the cerebral cortex.

#### Cell number quantification

To determine density, we performed a blinded, manual three-dimensional cell count using Fiji (NIMH). Within the CA1 hippocampus, we counted Hoechst-stained neuronal nuclei, since it was difficult to distinguish individual neuronal cells bodies immunoreactive with NeuN. Nuclei of non-neuronal cells were easy to distinguish and not included in our counts. Given that within the cerebral cortex neuronal density is lower, we used sections stained for NeuN, since Hoechst staining would include non-neuronal cells. Our cell count was based on the optical dissector method. We used a 16 by 16 by 41 µm three-dimensional counting frame for the hippocampus and 50 by 50 by 41 µm three-dimensional counting frame for Layer 5 of the cerebral cortex. Counting frame borders were divided into inclusion lines or exclusion lines (three each). Cell bodies were included in the count if they lie completely inside the counting frame or if they were touching an inclusion line. Cell bodies overlapping an exclusion line were not counted. Density was calculated by dividing average neuron cell numbers by the optical dissector volume.

### SDS-PAGE and immunoblotting

Hippocampi were extracted with T-PER protein extraction buffer (Pierce) supplemented with protease and phosphatase inhibitors (Roche). The lysates were centrifuged at 15,000 × *g* for 15 min at 4°C, and the supernatants were used for further analysis. Samples were prepared for standard SDS-PAGE and separated on 4–20% gradient acrylamide gel (Mini-PROTEAN TGX precast gels, Bio-Rad Laboratories) and then transferred onto 0.45 µm pore size PVDF membranes (Millipore) using semi-dry immunoblotting. Membranes were blocked with 5% nonfat milk in TBS-T (Tris-buffered saline with 0.2% Tween 20) for 1 h at room temperature and then incubated overnight at 4°C with primary antibodies diluted in 5% BSA in TBS-T. The following commercially available antibodies were used: mouse anti-β-amyloid (clone 6E10, Covance/BioLegend; 1:1,000), goat anti-CentA1 (Abcam; 1:500), and mouse anti-β-actin (Sigma-Aldrich, 1:1,000). Membranes were washed three times for 15 min in TBS-T, followed by incubation for 2 h at room temperature with HRP-conjugated donkey anti-goat or rabbit anti-mouse secondary antibodies (Bio-Rad Laboratories), diluted 1:2,000 in 5% nonfat milk in TBS-T. Membranes were washed three times for 15 min in TBS-T and then incubated with Pierce ECL Plus Western blotting substrate to detect Western blotted proteins. We used the Bio-Rad ChemiDoc imaging system to visualize protein bands. The Fiji software was used for Western blot quantification.

### Morris water maze (MWM) test

We used a previously described MWM protocol (Szatmari et al., 2021) to evaluate spatial learning and memory. Briefly, mice were handled and habituated to the testing facility for 3 d before testing. The water maze was a 1.4 m diameter white tank with a 10 cm diameter platform submerged ∼1 cm below the surface. The water temperature was kept at 22–24°C. To make the platform invisible during trials, nontoxic white washable paint was added to the water. Visual cues were placed around the tank for spatial reference. To verify the mice's visual ability and rule out motor deficits, we administered a visual platform test by removing the spatial cues and elevating the platform marked with a cue above the water's surface. During the acquisition phase of the hidden platform test, mice were given four trials/day for 8 consecutive days. If a mouse did not find the platform within 60 s, it was guided to it and kept on the platform for 15 s. To prevent hypothermia, mice were dried after each trial and placed into cages atop heating pads. On Day 9, mice were given a probe test without the platform. The EthoVision XT software (Noldus Information Technology) was used to record activity and performance. Total quadrant time, total number of entries into the target quadrant, total number of platform crossings, latency to first platform crossing, average distance to the platform center, and the number of trials to criterion for reaching platform center (20 s) were analyzed in a double-blinded manner.

### Golgi–Cox staining for dendritic spine density measurements

We employed the FD Rapid Golgi Staining protocol for Golgi–Cox staining of brain slices. Mice were deeply anesthetized and then decapitated. Each brain was rinsed with Milli-Q water and placed in an impregnation solution provided by the manufacturer. FD NeuroTechnologies performed sample processing and slice cutting at 100 µm thickness. Pyramidal neurons from the CA1 area of the hippocampus were imaged on slices using a Zeiss 780 confocal microscope (transmission mode; 488 nm wavelength laser; Plan-Neofluar 63× water objective with 1.3 numerical aperture). Each frame was acquired eight times and then averaged to obtain noise-free images. The number of spines/100 µm in the stratum lacunosum moleculare (SLM) and stratum radiatum (SR) regions of the CA1 pyramidal neurons was calculated using the Fiji software.

### Immunohistochemistry (IHC) staining and imaging

For IHC studies, mice were deeply anesthetized with a ketamine/xylazine cocktail until a lack of response to toe pinch was recorded. Mice were transcardially perfused with 50 ml PBS and 50 ml 4% PFA, and the brain was harvested and postfixed in 4% PFA for at least 24 h. Paraffin-embedded sagittal brain sections (5 µm thickness) were mounted on Superfrost Plus slides (Fisherbrand). Sections were deparaffinized and then either pretreated with 70% formic acid (6E10, Covance/BioLegend) or heat retrieved in a BioCare Decloaker (anti-GFAP; Abcam, anti-Iba1, Abcam). We used Rodent Block M (BioCare Medical) for 30 min at room temperature for blocking. Samples were incubated overnight at 4°C with primary antibody diluted in Van Gogh Diluent (BioCare Medical): 1:4,000 (6E10, Covance), 1:200 (Iba1, ab12267, Abcam), and 1:1,000 (anti-GFAP, Abcam). Sections were rinsed with TBS-T and incubated with secondary HRP-polymers (Mouse-on-Mouse, Rabbit-on-Rodent, or Goat-on-Rodent; BioCare Medical) for 30 min at room temperature. After several TBS-T rinses, the samples were reacted with Betazoid DAB (BioCare Medical), followed by TBS washes, then stained with hematoxylin, dehydrated, and coverslipped with Leica Micromount mounting medium.

#### Immunohistochemical imaging and image processing

Immunohistochemically stained sections were captured using a BZ-X800 digital microscope (Keyence) and analyzed using the Aperio ImageScope program as previously described (Chakrabarty et al., 2010). Amyloid plaque burden and neuroinflammation in the forebrain (cortex and hippocampus) were calculated using the Positive Pixel Count program within the Aperio ImageScope software. Four sections per brain (*n* = 3–4 mice/group) cut at 30 µm apart were analyzed and averaged for each mouse by an investigator blinded to the genotype. The final images and layouts were created using Creative Cloud Photoshop and Illustrator (Adobe).

### RNA extraction and analysis

**Figure 1.**
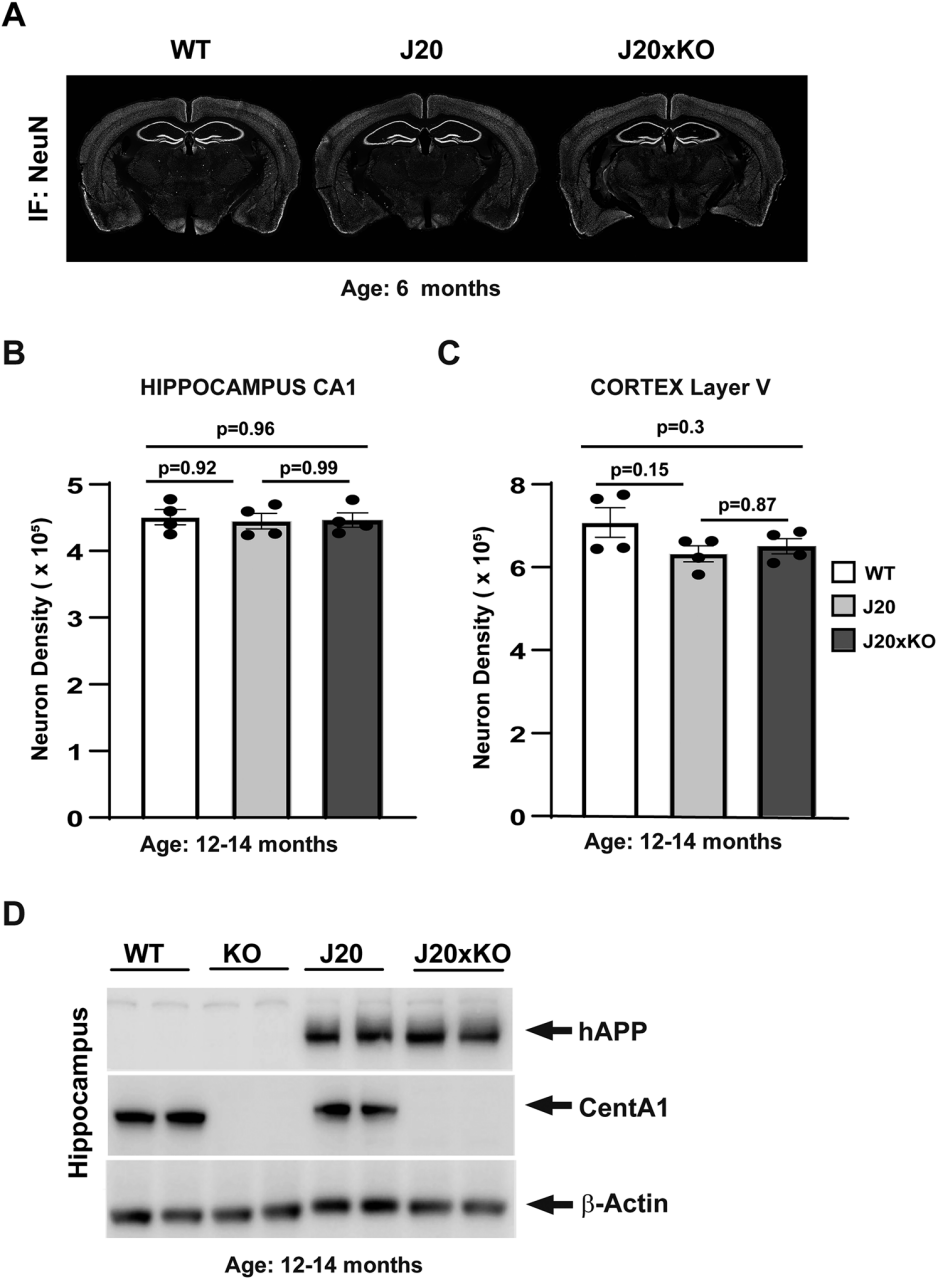
Generation and validation of J20 × CentA1 KO mice. ***A***, NeuN IHC of coronal sections from 6-month-old hAPP-J20 (J20); hAPP-J20 × CentA1 KO (J20xKO) and nontransgenic littermate (WT) mice show normal brain morphology in the J20xKO mice. ***B***, Neuron density in the CA1 region of the hippocampus evaluated on NeuN-stained coronal sections of 12–14-month-old J20; J20xKO and WT mice. ***C***, Neuron density in Layer 5 of the cortex evaluated on NeuN-stained coronal sections of 12–14-month-old J20; J20xKO and WT mice. ***D***, Immunoblots show the presence of hAPP and the complete lack of CentA1 protein in the hippocampus of J20xKO mice. β-Actin was used as a loading control. The number of mice was two/genotype. For full-length Western blots, see Extended Data Figure 1-1.

RNA was isolated from the frozen mouse cortical tissue and purified using the Qiagen RNeasy kit. RNA concentration was determined using Nanodrop (Thermo Fisher Scientific), and the quality was confirmed using the Bioanalyzer system with RNA pico chips. All samples had RNA integrity number score between 9.7 and 10 (Extended Date Fig. 6-1).

### Gene expression analysis

Gene expression analysis was performed as previously described (Bunner et al., 2023). Briefly, RNA was isolated from the fresh-frozen cortical tissue (3–4 mice/genotype). A 100 ng of the total RNA was hybridized with a reporter and capture probes for nCounter Gene Expression code sets (Neuropathology and Neuroinflammation by NanoString Technologies). Data were normalized to spiked positive controls and housekeeping genes using the NanoString nSolver Analysis system. Transcript counts less than the mean of the negative control transcripts plus 2STDEV for each sample was considered background. The gene profile was further analyzed in Python.

### Experimental design and statistical analysis

For all experiments, all genotypes were processed in parallel. Specific sexes (males only) were used for behavioral, IF, and immunohistology studies. GraphPad Prism (version 10 for Windows, GraphPad Software, www.graphpad.com) was used for most statistical analysis. Unpaired student's *t* test was used to compare two independent datasets. We used one-way ANOVA followed by Tukey's multiple-comparison test for multiple comparisons. Differences between genotypes or samples were considered significant at *p* < 0.05. Data are reported as mean ± SEM unless otherwise stated. GraphPad Prism was also used to calculate a 95% confidence intervals (CI) for the difference between means (Calin-Jageman and Cumming, 2019; Ho et al., 2019; Bernard, 2021). For DEG analysis, the significance was calculated with unpaired *t* test. The correlation and its significance of fold changes were calculated as Pearson's *R* (Python).

## Results

### Normal gross brain morphology of J20 and J20 × CentA1 KO mice

We generated J20 × CentA1 KO mice by crossing J20 mice with CentA1 KO mice. To assess whether CentA1 deletion affects gross brain structure in AD model mice, we compared the overall brain morphology between J20, J20 × CentA1 KO, and their nontransgenic (WT) littermates. NeuN immunostaining of coronal sections was indistinguishable between genotypes, suggesting that these animals have normal brain gross anatomy (Fig. 1*A*). Neuronal density was not statistically different between genotypes in the hippocampal CA1 region [Fig. 1*B*; neuronal density (×10^5^); WT, 4.30 ± 0.17; J20, 4.24 ± 0.2; J20xKO, 4.26 ± 0.15; *n* = 4 mice/genotype; WT vs J20, *p* = 0.92; 95% CI [−0.3872 to 0.5045]; WT vs J20xKO, *p* = 0.96; 95% CI [−0.4024 to 0.4893]; J20 vs J20xKO, *p* = 0.1; 95% CI [−0.4611 to 0.4306]; SEM; one-way ANOVA followed by Tukey's multiple-comparison test] and in Layer 5 of the cortex [Fig. 1*C*; neuronal density (×10^5^); WT, 6.92 ± 0.43; J20, 6.18 ± 0.27; J20xKO, 6.36 ± 0.34; *n* = 4 mice/genotype; WT vs J20, *p* = 0.15; 95% CI [−0.2642 to 1.772]; WT vs J20xKO, *p* = 0.3; 95% CI [−0.4459 to 1.590]; J20 vs J20xKO, *p* = 0.87; 95% CI [−0.1.200 to 0.8365]; SEM; one-way ANOVA followed by Tukey's multiple-comparison test]. We also confirmed the lack of CentA1 protein and the expression of the hAPP transgene in the J20xKO mice in the hippocampus using Western blotting (Fig. 1*D*). Full-length Western blots are presented on Extended Data Figure 1-1.

10.1523/ENEURO.0063-25.2025.f1-1 Figure 1-1 **Biochemical validation of J20xKO mice**: Full-length western blots showing the presence of transgene and lack of CentA1 immunoreactivity in the hippocampus of J20xKO mice at 12 months of age. β-Actin re-probing of the blots shows equal amount of protein samples loaded on the gels. The number of mice was 2/genotype. Download Figure 1, DOCX file.

### Deletion of CentA1 rescues spatial learning and memory in AD model mice

**Figure 2.**
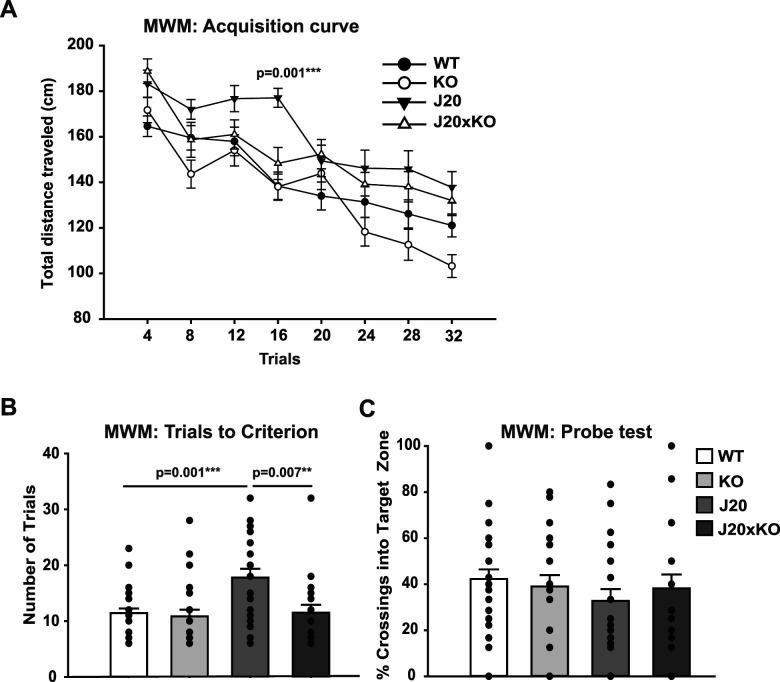
Lack of CentA1 protein rescues spatial memory deficit in the J20 mice. ***A***, The acquisition curve of hippocampal-dependent spatial learning and memory was evaluated using the MWM test. The distance traveled to reach the target escape platform decreased across training days for all genotypes. However, the J20 mice demonstrated a significant deficit in learning acquisition by Day 4 of training compared with J20xKO mice (*p* = 0.001). ***B***, The number of trials needed to reach the predefined criterion of performance (escape latency maximum of 20 s) was significantly higher for the J20 mice compared with WT (*p* = 0.001) and J20xKO mice (*p* = 0.007), indicating rescue of learning deficit by the lack of CentA1. ***C***, All genotypes performed similarly on the memory probe test administered on Day 9. The number of mice was WT, *n* = 26; KO, *n* = 24; J20, *n* = 25; and J20xKO, *n* = 20. All behavior data are presented as mean ± SEM.

Next, we assessed whether the deletion of CentA1 influences the behavioral phenotypes of the J20 mice through the MWM task to evaluate hippocampus-dependent spatial learning and memory. We trained the mice of four genotypes, WT, CentA1 KO, J20, and J20 × KO (aged 6–7 months), using four trials/day for 8 d, with an intertrial interval of 20–30 min. As animals learn the location of the hidden platform, the distance traveled to the target location decreased with training for all genotypes, as indicated by a two-way repeated–measure ANOVA for the genotype and the trial block (*F*_(3,637)_ = 12.27; *p* = 0.001; *η*^2^*p* = 0.06). Holm–Sidak post hoc analyses indicated that the J20 animals had a slower learning rate than other genotype groups, as shown at Day 4 of training (compared with WT, *t*_(44)_ = 3.16; *p* = 0.001; average distance traveled for Day 4 training, mean ± SEM; WT, 138.37 ± 5.95 cm; *n* = 26; KO, 138.07 ± 6.19 cm; *n* = 24; J20, 177.08 ± 6.07 cm; *n* = 25; J20xKO, 148.31 ± 6.78 cm; *n* = 20; Fig. 2*A*). The number of trials to reach a predefined group criterion of performance, which was set to 20 s of escape latency (Colgan et al., 2018), also indicated a slower learning rate for J20 mice (Fig. 2*B*). A one-way (genotype) ANOVA revealed a significant genotype effect (*F*_(3,91)_ = 6.37; *p* = 0.001; *η*^2^*p* = 0.17), and Holm–Sidak post hoc analyses indicated that it took significantly longer for the J20 mice to reach this predefined criterion compared with WT (WT vs J20, *t*_(49)_ = 3.18; *p* = 0.001). This effect was rescued in J20xKO (J20xKO vs J20, *t*_(20)_ = 3.30; *p* = 0.007; the average number of trials to reach criterion, mean ± SEM; WT, 11.42 ± 0.83 trials; *n* = 26; KO, 10.83 ± 1.21 trials; *n* = 24; J20, 17.80 ± 1.58 trials; *n* = 25; J20xKO, 11.50 ± 1.40 trials; *n* = 20; Fig. 2*B*). However, all genotypes showed similar performance on the memory probe test administered on Day 9 [one-way (genotype) ANOVA on the percentage of crossings into the target search zone, *F*_(3,91)_ = 0.66; *p* = n.s.; *η*^2^*p* = 0.02; Fig. 2*C*]. These results suggest that J20 animals have a spatial learning deficit and the removal of CentA1 rescues this phenotype.

### Deletion of CentA1 rescues dendritic spines in the hippocampus of J20 mice

**Figure 3.**
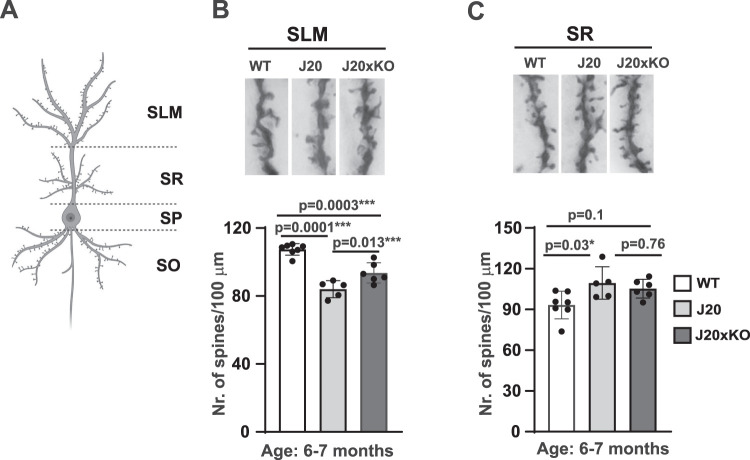
Lack of CentA1 rescues dendritic spine density in the hippocampus of J20 mice. ***A***, The diagram depicts imaged dendritic segments of Golgi-stained CA1 neurons within distinct hippocampal layers: stratum oriens (SO); stratum pyramidale (SP); stratum radiatum (SR), and stratum lacunosum moleculare (SLM; created with BioRender.com). ***B***, Significantly reduced dendritic spine density in the hippocampus lacunosum moleculare (SLM) neurons of J20 mice compared with WT littermates (*p* = 0.001). CentA1 KO partially restored dendritic spine density in the SLM of J20 mice (*p* = 0.02). ***C***, Dendritic spine density in the SR of the hippocampus in J20 mice is significantly higher compared with WT littermates (*p* = 0.03), while CentA1 KO does not rescue this effect (*p* = 0.9). The number of mice was 4–5/genotype, and five neurons/animal were analyzed. All data are presented as mean ± SEM.

Loss of dendritic spines and synapses are early pathological hallmarks of AD that precede plaque deposits in the AD brain (Dorostkar et al., 2015; Mijalkov et al., 2021). Therefore, we analyzed the effect of CentA1 KO on the loss of dendritic spines in the hippocampus CA1 neurons of J20 mice. We counted the number of dendritic spines in the SLM and the SR of the hippocampus of J20, J20xKO mice, and their WT littermates (Fig. 3*A*). Five neurons/animal were included in the analysis (*n* = 4–6 mice/genotype). We found that spine density was reduced in the SLM of J20 mice compared with WT, while CentA1 KO attenuated this effect (Fig. 3*B*; number of spines/100 µm; WT = 107.4 ± 1.28; J20 = 84.05 ± 1.88; J20xKO = 93.6 ± 2.23; WT vs J20, *p* = 0.0001, 95% CI [16.09–30.64]; WT vs J20xKO, *p* = 0.0003; 95% CI [6.915–20.74]; J20 vs J20xKO, *p* = 0.012; 95% CI [−17.06 to −2.012]; SEM; one-way ANOVA followed by Tukey's multiple-comparison test). We found a small but statistically significant increase in the spine number in the SR area in J20 mice compared with WT, while in the J20xKO mice, spine density was not statistically different when compared with WT mice (Fig. 3*C*; number of spines/100 µm; WT = 93.25 ± 3.83; J20 = 109.5 ± 4.52; J20xKO = 105.3 ± 2.6; WT vs J20, *p* = 0.03; 95% CI [−31.08 to −1.381]; WT vs J20xKO, *p* = 0.10; 95% CI [−26.16 to 2.054]; J20 vs J20xKO, *p* = 0.76; 95% CI [−11.18 to 19.53]; SEM; one-way ANOVA followed by Tukey's multiple-comparison test).

### Deletion of CentA1 reduces amyloid plaque deposition and neuroinflammation in J20 mice

**Figure 4.**
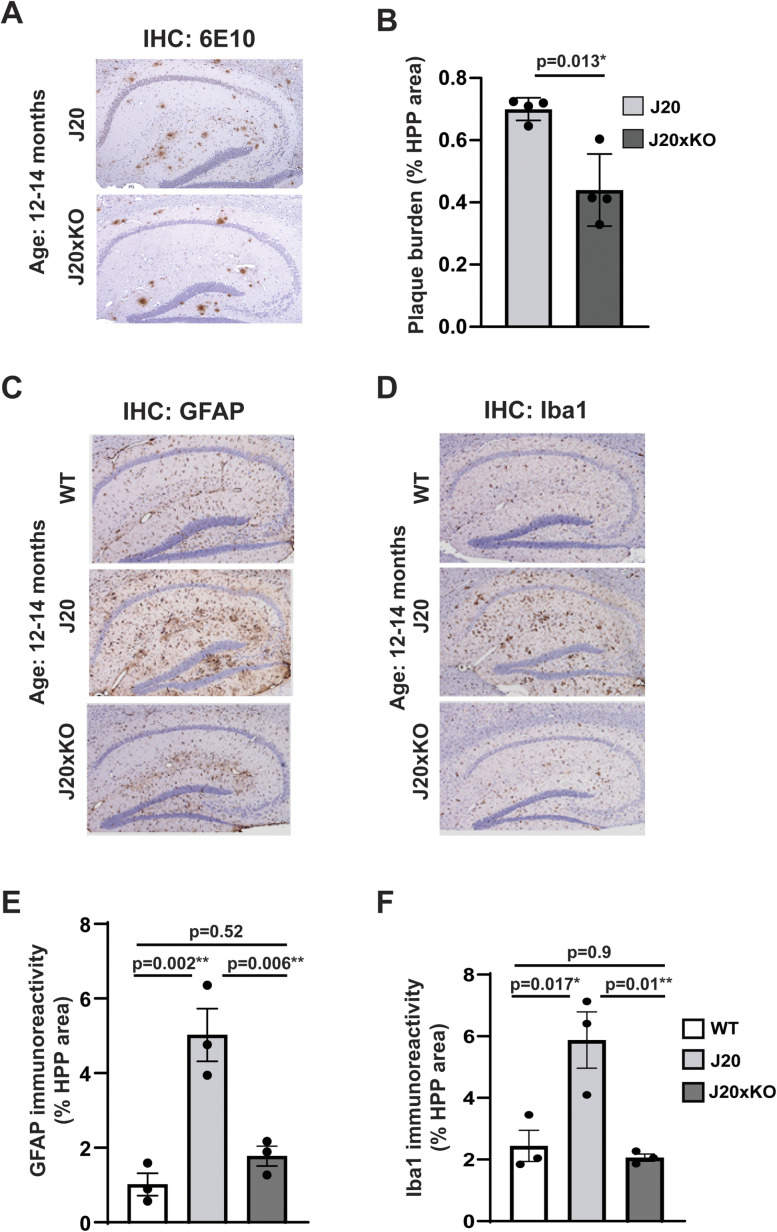
Effect of CentA1 KO on the histopathological hallmarks of AD in the hippocampus of the J20 mice. ***A***, Representative images of amyloid plaque burden in the hippocampus in paraffin-embedded whole–brain sections from J20 and J20xKO mice. ***B***, The graph shows significantly reduced plaque burden in the hippocampus of J20xKO mice compared with J20 mice (*p* = 0.013). ***C***, Representative images of GFAP immunoreactivity in the hippocampus in paraffin-embedded whole–brain sections from WT, J20, and J20xKO mice. ***D***, Representative images of Iba-1 immunoreactivity in the hippocampus in paraffin-embedded whole–brain sections from WT, J20, and J20xKO mice. ***E***, The graph shows significantly increased astrogliosis in the hippocampus of J20 mice compared with control mice (*p* = 0.006) and J20 mice on CentA1 KO background (*p* = 0.01), while there was no significant difference between WT and J20xKO genotypes (*p* = 0.13). ***F***, The graph shows significantly increased microgliosis in the hippocampus of J20 mice compared with control mice (*p* = 0.003). CentA1 KO rescued microgliosis in the J20 mice (*p* = 0.001). The number of mice was 3–4/genotype. All data are presented as mean ± SEM.

**Figure 5.**
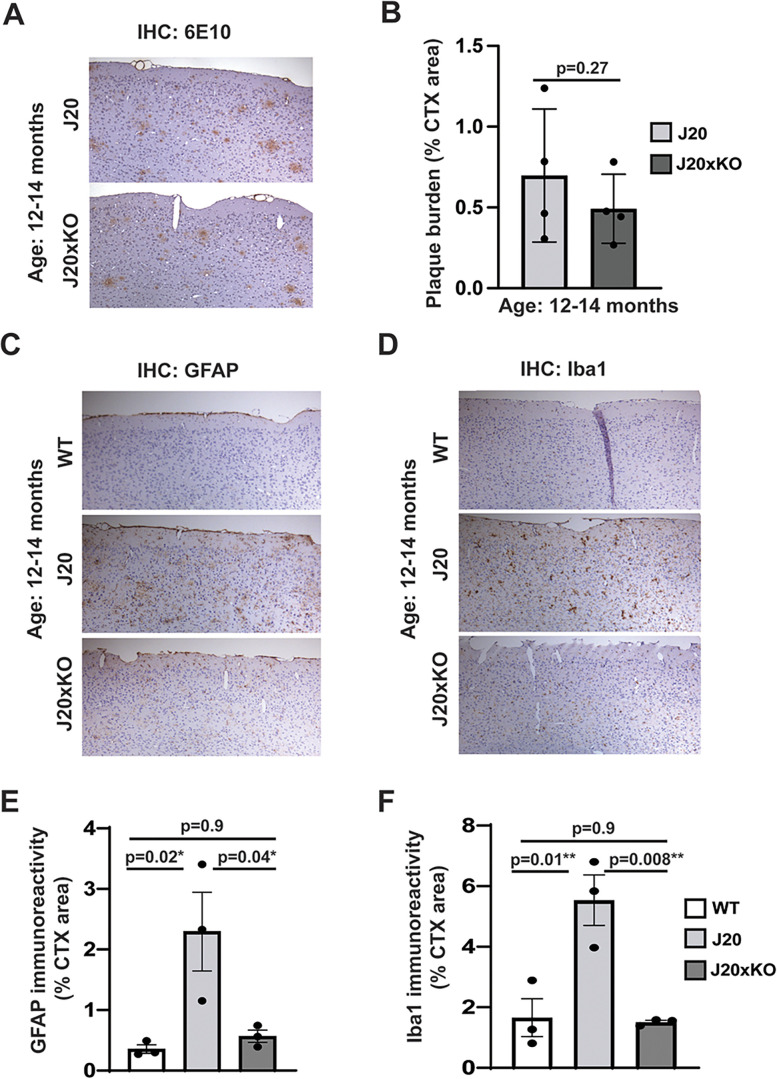
Effect of CentA1 KO on the histopathological hallmarks of AD in the cortex of the J20 mice. ***A***, Representative images of cortical amyloid plaque burden in paraffin-embedded whole–brain sections from J20 and J20xKO mice. ***B***, The graph shows the plaque burden in the cortex of J20 mice on CentA1 KO background (*p* = 0.27); however, the difference did not reach statistical significance. ***C***, Representative images of GFAP immunoreactivity in the cortex in paraffin-embedded whole–brain sections from WT, J20, and J20xKO mice. ***D***, Representative images of Iba-1 immunoreactivity in the cortex in paraffin-embedded whole–brain sections from WT, J20, and J20xKO mice. ***E***, The graph shows significantly increased astrogliosis in the cortex of J20 mice compared with control mice (*p* = 0.01) and J20 mice on CentA1 KO background (*p* = 0.04). ***F***, The graph shows significantly increased microgliosis in the cortex of J20 mice compared with control mice (*p* = 0.03). CentA1 KO reduced microgliosis to a level that was not statistically significant from WT mice (*p* = 0.89).

To evaluate whether CentA1 regulates Aβ production in J20 animals, we compared the amyloid deposition in the hippocampus and the neocortex between J20 and their J20xKO littermates at age 12–14 months (Fig. 4). Quantification of Aβ plaque immunoreactivity in the hippocampus (Fig. 4*A*,*B*) showed a significant reduction in plaque burden in the J20 mice lacking CentA1 (the percentage of the hippocampal area covered; J20, 0.64 ± 0.34; J20xKO, 0.403 ± 0.21; *n* = 4 mice/genotype; *p* = 0.0123; 95% CI [−0.4143 to −0.062]; SEM; two-tailed unpaired *t* test). However, amyloid deposition in the neocortex showed no significant difference between J20 and J20xKO mice (Fig. 5*A*,*B*; the percentage of the cortical area covered; J20, 0.64 ± 0.34; J20xKO, 0.45 ± 0.24; *n* = 4 mice/genotype; *p* = 0.27; 95% CI [−0.551 to 0.175]; SEM; two-tailed unpaired *t* test). Thus, CentA1 may regulate the amyloid plaque formation in a brain region-specific manner.

Next, we evaluated astrogliosis and microgliosis in the hippocampus (Fig. 4*C*–*F*), using immunohistochemical analysis with antibodies against GFAP (Fig. 4*C*) and Iba1 (Fig. 4*D*), respectively. Quantitation of GFAP immunoreactivity in the hippocampus (Fig. 4*E*) indicated a substantial increase in astrocytic activation in AD model mice compared with WT mice. This increased astrocyte activation was significantly reduced in J20xKO compared with J20 mice (Fig. 4*E*; the percentage of the hippocampal area covered; WT, 1.01854 ± 0.26; *n* = 3; J20, 5.0226 ± 0.84; *n* = 3; J20xKO, 02.5668 ± 0.81, *n* = 3; WT vs J20, *p* = 0.0023; 95% CI [−6.043 to −1.966]; WT vs J20xKO, *p* = 0.52; 95% CI [−2.797 to 1.281]; J20 vs J20xKO, *p* = 0.006; 95% CI [1.208–5.285]; SEM; one-way ANOVA followed by Tukey's multiple-comparison test). In the cortex, the J20xKO group had a significantly reduced level of astrogliosis than the J20 mice (Fig. 5*C*,*E*; the percentage of the cortex area covered; WT, 0.355 ± 0.07; *n* = 3; J20, 2.29 ± 0.54; *n* = 3; J20xKO, 0.56 ± 0.19, *n* = 3; WT vs J20, *p* = 0.026; 95% CI [−3.599 to −0.283]; WT vs J20xKO, *p* = 0.92; 95% CI [−1.870 to 1.447]; J20 vs J20xKO, *p* = 0.04; 95% CI [0.0714–3.388]; SEM; one-way ANOVA followed by Tukey's multiple-comparison test).

Widespread reactive microgliosis, indicated by increased immunoreactivity to Iba-1, was also observed in the brain of the J20 mice, especially in and around the hippocampus (Fig. 4*D*). ANOVA analysis showed a statistically significant increase in the expression of Iba-1 in J20 mice compared with WT and J20xKO in the hippocampus (Fig. 4*F*; the percentage of the hippocampal area covered; WT, 2.80 ± 0.4; *n* = 3; J20, 4.8 ± 1.17; *n* = 3; J20xKO, 2.06 ± 0.1, *n* = 3; WT vs J20, *p* = 0.017; 95% CI [−6.070 to −0.7970]; WT vs J20xKO, *p* = 0.89; 95% CI [−2.254 to 3.019]; J20 vs J20xKO, *p* = 0.01; 95% CI [1.180–6.453]; SEM; one-way ANOVA followed by Tukey's multiple-comparison test). Similar effects were observed in the cortex (Fig. 5*D*,*F*; percentage of the cortex area covered; WT, 2.2 ± 0.7; *n* = 3; J20, 4.47 ± 1.21; *n* = 3; J20xKO, 2.4 ± 0.9, *n* = 3; WT vs J20, *p* = 0.009; 95% CI [−6.501 to −1.262]; WT vs J20xKO, *p* = 0.98; 95% CI [−2.473 to 2.766]; J20 vs J20xKO, *p* = 0.008; 95% CI [1.408–6.647]; SEM; one-way ANOVA followed by Tukey's multiple-comparison test).

### Transcriptome profiling of J20 mice on CentA1 KO background

**Figure 6.**
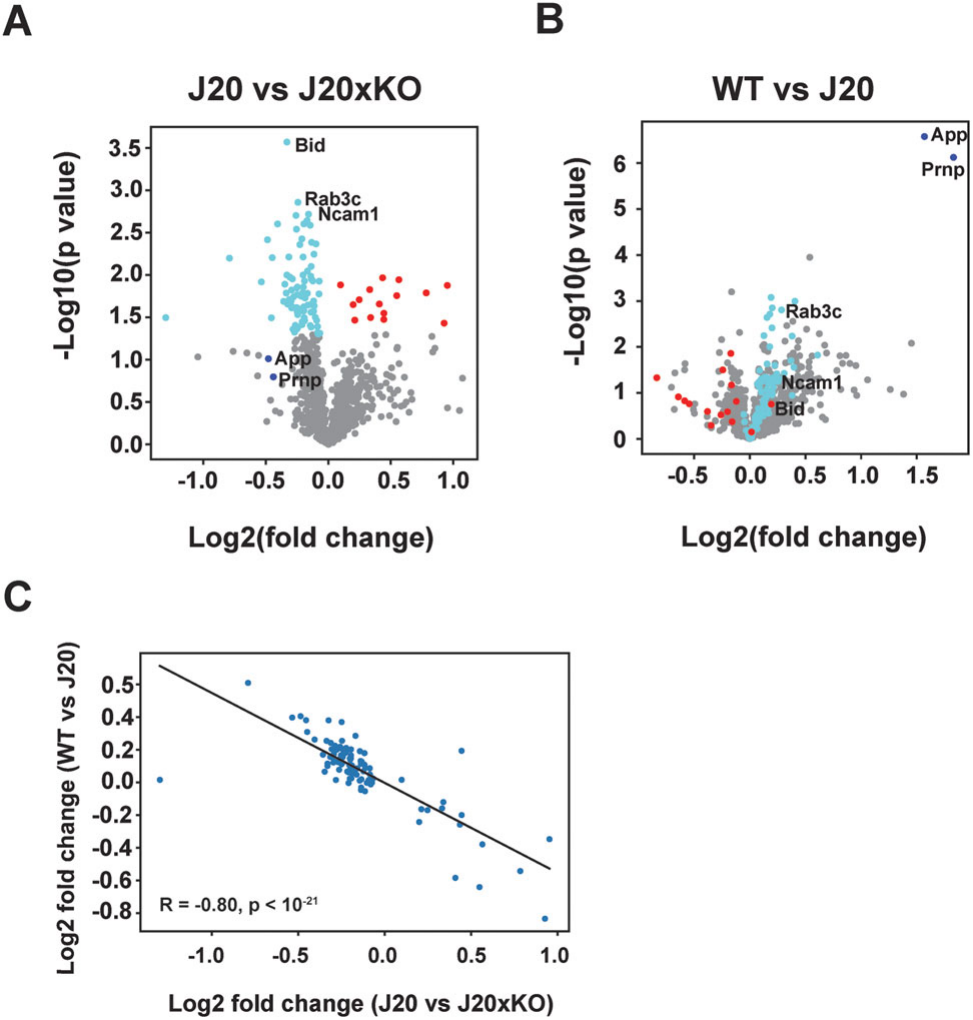
DEGs between genotypes identified with NanoString nCounter profiling. ***A***, ***B***, Volcano plot analysis of DEGs between J20 and J20xKO mice (***A***) and between WT and J20 mice (***B***). Colors indicate genes that show significantly down- (cyan) or upregulated (red) between J20 and J20xKO. ***C***, The graph shows that the fold changes in DEGs between J20 versus J20xKO and WT versus J20 are strongly anticorrelated (Pearson's *R* −0.8; *p* < 10^−21^). RNA integrity scores for individual samples are reported in Extended Data Figure 6-1. The list of all identified DEGs is reported in Extended Data Figure 6-2.

To gain insights into the molecular mechanisms by which CentA1 influences AD-related phenotypes, we employed the NanoString nCounter platform. We evaluated the expression profile of genes associated with neurodegeneration, neuroinflammation, and aging in our cohorts. We isolated RNA from the brains of WT, J20, and J20xKO mice. We analyzed DEGs between genotypes using a volcano plot analysis (Fig. 6). We identified 83 genes that are significantly downregulated in J20xKO (cyan) compared with J20 mice, while 15 genes are upregulated (red) (*p* < 0.05; Fig. 6*A*; Extended Data Fig. 6-2). Almost all the genes downregulated in J20xKO versus J20 (82 out of 83) were upregulated in J20 compared with WT (Fig. 6*B*, red points; Extended Data Fig. 6-2). Conversely, most genes significantly downregulated in J20xKO compared with J20 were upregulated in J20 (13 out of 15; Fig. 6*A*,*B*, cyan plots; Extended Data Fig. 6-2). For these significantly regulated genes, the gene expression fold changes between J20 versus J20xKO and J20 versus WT were strongly anticorrelated (Pearson's *R* −0.8; *p* < 10^−21^; Fig. 6*C*).

10.1523/ENEURO.0063-25.2025.f6-1 Figure 6-1 **Report of RNA integrity (RIN) scores.** All RNA integrity (RIN) scores for the samples analyzed in Figure 6 were between 9.7–10. Download Figure 6, DOCX file.

10.1523/ENEURO.0063-25.2025.f6-2 Figure 6-2 **List of differentially expressed genes (DEGs) identified by NanoString nCounter gene expression profiling.** Table shows all differentially expressed genes (DEGs) in the forebrain of J20, J20xKO mice and their wild-type littermates. Download Figure 6-2, DOCX file.

## Discussion

In the present study, we evaluated the involvement of CentA1 in the pathophysiology of AD using J20 mice, a well-established mouse model of AD (Mucke et al., 2000; Wright et al., 2013; Lee et al., 2022). J20 crossed with CentA1 KO (J20xKO) ameliorated many AD-related phenotypes, including behavioral deficits, dendritic spine loss, amyloid plaque deposition, and neuroinflammation. Furthermore, DEG analysis showed that lack of CentA1 can restore the expression of many dysregulated genes in the J20 mice.

Our findings align with previous research showing that CentA1 is transiently upregulated by Aβ42 application and that downregulation of CentA1 via shRNA reduces Aβ42-induced cellular phenotypes in organotypic hippocampal slice cultures (Szatmari et al., 2013). Further studies using CentA1 KO models have indicated that CentA1 acts as a negative regulator of dendritic spine density, synaptic long-term potentiation, and learning and memory functions (Szatmari et al., 2021). However, the specific role of CentA1 in AD-related phenotypes in vivo has not been analyzed. This study demonstrated that CentA1 mediates AD-related phenotypes in vivo by generating AD model mice lacking CentA1.

CentA1 deletion (J20xKO) improved many pathological phenotypes observed in the J20 animals. Since amyloid plaque formation is reduced without a change in APP expression levels, CentA1 may play a role in amyloid production, clearance, or aggregation. Prior research similarly indicates that CentA1 downregulation in neurons improves AD-related cellular phenotypes when soluble amyloid is directly applied to organotypic slices (Szatmari et al., 2013). Thus, CentA1 may function both upstream and downstream of Aβ42, contributing to multiple facets of AD pathology.

Our inflammation analysis suggests that CentA1 plays a role in glial activation in J20, as J20xKO mice showed significantly lower astrocytosis and microgliosis. These results are consistent with previous studies showing that CentA1 is highly expressed in the brain (Zhang et al., 2016), mainly in neurons and to a lower extent in glial cells, and that the level of CentA1 increases in AD (Reiser and Bernstein, 2002, 2004). Moreover, neuronal CentA1 was shown to mediate Aβ42-induced spine loss (Szatmari et al., 2013). Thus, it appears that CentA1 contributes to AD-related pathology in both neurons and glial cells.

DEG analysis revealed that the deletion of CentA1 in J20 mice leads to the downregulation of numerous genes, particularly those involved in Ca^2+^ signaling, such as *Grin2a*, *Grin2b*, *Ryr2*, *Cacna1b*, and *Cacna1c*. Many of these genes were upregulated in J20 mice compared with WT mice, suggesting that aberrant Ca^2+^ signaling plays a significant role in the pathological development associated in the J20 model. This finding aligns with the existing research indicating that NMDA receptor dysregulation is a core component of AD pathology (Palop et al., 2007; Talantova et al., 2013; Zhang et al., 2021). Importantly, CentA1 deletion appears to mitigate this abnormal signaling gene profile. Thus, CentA1 deletion appears to ameliorate many pathological phenotypes in J20 mice, at least partially by restoring the normal expression of genes implicated in Ca^2+^ signaling. Our data provide evidence for a neuroprotective outcome of lowering the level of CentA1 in the brain.

### Study limitations

The present study concentrated on the role of CentA1 in the development of AD-like phenotypes in the J20 mouse model, which has amyloid pathology, however does not have tau pathology (Mucke et al., 2000); therefore, continued investigation using other mouse models, such as the 3xTg-AD (Javonillo et al., 2021), is warranted. Biological sex differences associated with AD onset and progression are well documented; however the underlying biological mechanisms are poorly understood (Aggarwal and Mielke, 2023; Lopez-Lee et al., 2024a,b). For instance, males are at higher risk for mid-life dementia risk and at lower risk for late-onset AD than females, while women are affected more frequently and severely at both the cognitive and neuropathological level (Rosende-Roca et al., 2025). As only the male sex of the J20 mice was used, future studies should include both sexes. We acknowledge that the present study was entirely performed on a mouse model and validation of these findings on autopsied human brain samples is warranted.

Another limitation to note is the use of noncomprehensive RNA analysis. We employed nCounter as an exploratory approach to analyze known, well-defined factors associated with neurodegeneration. We acknowledge that follow-up studies using RNA-Seq are necessary for a comprehensive view of the transcriptome and to detect novel targets of CentA1 signaling associated with neurodegeneration.

## Synthesis

Reviewing Editor: Timothy Jarome, Virginia Polytechnic Institute and State University Decisions are customarily a result of the Reviewing Editor and the peer reviewers coming together and discussing their recommendations until a consensus is reached. When revisions are invited, a fact-based synthesis statement explaining their decision and outlining what is needed to prepare a revision will be listed below. The following reviewer(s) agreed to reveal their identity: NONE.

Overall, the manuscript is interesting and straightforward. However, several concerns need to be addressed:

Major:

1. It is unclear why only male mice were used in this study, as it is generally the standard now to include both sexes in research. Please include data from female mice or provide scientifically sound justification for excluding female mice. If the latter, please at minimum include in the discussion how this omission affects the conclusions and interpretations made in this study.

2. Fig 1A: While we agree that the NeuN staining looks similar between the different genotypes, one example section from each genotype seems insufficient to draw the conclusion that these brains are grossly normal in their anatomy. Please provide some sort of quantification, such as brain weight, brain size, cortical widths, hippocampus layer widths, etc.

3. The sample sizes seem to change between experiments that would reasonably have been conducted on the same set of mice or even the same brain sections (specifically, in Fig 3B and C, n for J20 mice goes from 5 for SLM to 4 for SR; in Fig 4E and F, n for WT and J20xKO mice goes from 3 in hippocampus to 4 in cortex; in Fig 4H and I, n for WT mice goes from 4 in hippocampus to 3 in cortex). This suggests that some animals might have been excluded from certain experiments. If so, what is the criteria for exclusion of animals? Please include an explanation in the manuscript text.

4. Related, in figure 3 there are 5 J20 datapoints for SLM but 4 shown on the graph for SR, with an average for SR that looks too high. Is there a data point off the graph? If so, please adjust the axis so that it is visible. Furthermore, the text states 4-6 mice per group, and the legend states 4-5, but WT has 7. (and, if the above supposition is correct, the minimum would be 5 for J20, so correct range would be 5-7?).

5. Figures 1 and 2 include data on CentA1 KO animals, but this genotype was not included in the rest of the figures. Please include from CentA1-KO animals for the rest of the figures for completeness and to help put the study in context.

6. Please include example images for plaques, GFAP astrocytes, and Iba1 microglia in the cortex for Fig 4C, F, and I.

7. Fig 5: Fig 5C shows that a lot of the changes in gene expression in J20 vs control mice are changed in the opposite direction when CentA1 is knocked out in J20 mice, suggesting that J20xKO mice might thus look more similar to WT mice in gene expression. This point might be further strengthened by looking directly at the gene expression changes between WT vs J20xKO mice, which should be included if available.

8. It is not specified, but appears that differential RNA expression was not adjusted for multiple comparisons. This should be done and only genes passing a multiple corrections threshold should be reported as significant.

9. A limitations section should be included in the discussion section. A few limitations to note include use of male only mice, non-comprehensive RNA analysis due to use of nCounter instead of RNA-seq, and general limitations of findings in animal models without corresponding analysis of human tissues.

10. Some discussion, even if speculative, of the mechanism by which CentA1 may be achieving these effects is needed, both for contextualizing the study, but also for demonstrating how future directions could lead to further insights. For example, if "CentA1 signaling represents a promising therapeutic target for treating AD," how could it be targeted?

Minor comments:

1. Figure 1 uses "ADAP1", whereas the manuscript text uses "CentA1." The authors should consider sticking with one terminology throughout the manuscript for clarity.

2. Figure 1B: The effect of the knockout is clear from the blots; however, it seems odd to not quantify or statistically analyze and compare the bands (probably due to there only being 2 mice in some genotypes). It is recommended to increase the n if possible to include quantification and statistics if possible.

3. While the authors cite that CentA1 is increased in the brains of AD patients, it is unclear whether this is replicated in J20 mice. This piece of data would help put the findings in this study in context. Please provide quantification of CentA1 expression in J20 mice compared with WT mice if possible.

4. Please include the statistical comparison bar between WT and J20xKO groups in Fig 3B and 3C, especially since that comparison is significant in Fig 3B.

5. The figure legend colors in Fig 4 don't match the actual colors of the bars.

6. There were a few places where wording is a little strong and should be revised. In describing Figs 3B, the authors state that CentA1KO "rescued" the spine density reduction, but J20xKO mice spine density is still significantly reduced compared with that in WT mice. In 3C, authors state that J20xKO mice dendrites showed "normal" spine number, but the J20xKO spine density looks closer to J20 levels than WT levels.

7. Please explain the y-axis for Fig 2A. Why is this not just distance traveled?

8. The authors state in the discussion that "amyloid plaque formation is reduced without a change in APP expression levels" in J20xKO mice, but APP expression levels were not actually assessed and quantified in these mice vs J20 mice. It would strengthen the paper to include this result if available; otherwise, this statement should be removed.

9. Pearson test assumes normality as does t-test - please test for normality, and if a given dataset fails, a non-parametric test would be more appropriate.

10. Full-length western blots should be provided as supplementary material.

11. Please note age range for MWM in figure 2

12. From frozen brain, I expect that the RNA integrity was good, but this is not explicitly reported. A table with RIN scores for RNA (or, if all quite high, a statement that "all samples had RIN scores > X" would be helpful in this regard, these data should be readily available from the bioanalyzer runs.

13. The quality of the images (non-graph) are sub-optimal, this should be corrected for the revised manuscript.

## Author Response

Response to Reviewers We thank the reviewers for their constructive feedback. Below we provide a point-by-point response. Reviewer comments are in italics; our responses follow.

Major Points 1. Why were only male mice used? Please include data from female mice or provide scientifically sound justification.

Response: We acknowledge this limitation. We cannot provide female data in this revision, but we now explicitly state it in the "Study Limitations" section (page 15): "Biological sex-differences associated with AD onset and progression are well documented... As only the male sex of the J20 mice was used, future studies should include both sexes." 2. Fig 1A: One representative section per genotype is insufficient to conclude brains are grossly normal.

Response: We added quantification of neuronal density in both hippocampal CA1 and cortical layer V. (Page 9, lines 217-227) "Neuronal density was not statistically different between genotypes in the hippocampal CA1 region (Fig. 1B ... WT=4.30{plus minus}0.17; J20: 4.24{plus minus}0.2; J20xKO: 4.26{plus minus}0.15 ...)." 3. Sample sizes change between experiments; clarify exclusion criteria.

Response: All n-values are now consistently reported in figure legends (for example, Fig. 4E: WT n=3, J20 n=3, J20xKO n=3). Animals were only excluded when tissue quality was insufficient for a specific assay. This clarification is in Methods.

4. CentA1 KO animals missing in later figures.

Response: CentA1 KO animals are included in early results (Fig. 1 and Fig. 2). For pathology and transcriptome studies we focused on WT, J20, and J20xKO, as these comparisons directly address AD-related phenotypes. This rationale is noted in the Results and in the Study Limitations section.

5. Please include example images for plaques, GFAP, and Iba1 in the cortex.

Response: We have now added representative cortical images. See Figures 5A (plaques), 5C (GFAP), and 5D (Iba1).

6. RNA differential expression not corrected for multiple comparisons.

Response: Our NanoString analysis was designed as a targeted exploratory screening to prioritize candidate pathways for follow-up, not to make definitive claims about individual transcripts. For this reason, we reported DEGs at p < 0.05 (uncorrected) together with effect sizes. Crucially, our main result does not depend on individual gene calls: the global fold-change relationship between groups shows a strong anti-correlation (Pearson's R = -0.8) (Fig. 6C), which is independent of any particular significance cutoff.

In the revised manuscript, we acknowledge the limitation and note it in the Study limitations section, page 15, lines 370-373: "Another limitation to note is the use of non-comprehensive RNA analysis. We employed nCounter as an exploratory approach... follow-up studies using RNA-Seq are necessary for a comprehensive view." 7. Some discussion of mechanism is needed.

Response: We expanded the Discussion to include mechanisms involving Ca²⁺ signaling and amyloid pathways. Page 14, lines 346-350: "Deletion of CentA1 ... leads to the downregulation of numerous genes, particularly those involved in Ca²⁺ signaling, such as Grin2a, Grin2b, Ryr2, Cacna1b, and Cacna1c ... Thus, CentA1 deletion ameliorates many pathological phenotypes by restoring normal expression of genes implicated in Ca²⁺ signaling." 8. Add a limitations section.

Response: A dedicated Study Limitations section has been added on page 15-16. It covers 1) male-only animals 2) absence of tau pathology in J20 3) nCounter vs RNA-seq 4) need for validation in human tissue.

Minor Points 1. Terminology inconsistent (ADAP1 vs CentA1).

Response: We now consistently use "CentA1" throughout (title, abstract, figures).

2. Fig 1B blot quantification missing.

Response: Blots validate absence of CentA1 and the presence of APP transgene but were not quantified; clarified in figure legend.

3. Quantify CentA1 expression in J20 vs WT.

Response: We do not have these data. The text has been softened. Page 14, lines 334-335: "Since amyloid plaque formation is reduced without a change in APP expression levels, CentA1 may play a role in amyloid production, clearance, or aggregation." 4. Statistical bars missing in Fig. 3.

Response: Now included.

5. Figure legend colors inconsistent.

Response: Corrected.

6. Wording too strong ("rescued," "normal").

Response: Revised wording throughout; for example, Fig. 3B legend now states "attenuated" rather than "rescued." 7. Explain y-axis for Fig. 2A.

Response: Clarified as "average distance traveled (cm)." See Fig. 2 legend.

8. APP expression not quantified.

Response: APP expression validated by immunoblot (Fig. 1D-E) but not quantified. Text revised to avoid overstatement.

9. Normality assumptions not tested.

Response: Clarified in Methods: "Normality was assumed; CIs reported. Future studies may use non-parametric tests where appropriate." 10. Provide full-length western blots.

Response: Added as supplementary material.

11. Age range for MWM missing.

Response: Added in Methods: "... four genotypes ... (aged 6-7 months) ..." 12. Report RNA integrity (RIN) scores.

Response: Added in Methods: "All samples had RNA integrity number (RIN) score between 9.7-10." Full report was added as supplementary material.

13. Image quality suboptimal.

Response: Replaced with higher-resolution images. See Figs. 4 and 5.

## References

[B1] Aggarwal NT, Mielke MM (2023) Sex differences in Alzheimer's disease. Neurol Clin 41:343–358. 10.1016/j.ncl.2023.01.001 37030962 PMC10321561

[B2] Aggensteiner M, Reiser G (2003) Expression of the brain-specific membrane adapter protein p42IP4/centaurin alpha, a Ins(1,3,4,5)P4/PtdIns(3,4,5)P3 binding protein, in developing rat brain. Brain Res Dev Brain Res 142:77–87. 10.1016/S0165-3806(03)00033-6 12694946

[B3] Bernard C (2021) Estimation statistics, one year later. eNeuro 8:ENEURO.0091-21.2021. 10.1523/ENEURO.0091-21.2021 PMC8021395 33795354

[B4] Bunner W, et al. (2023) Behavioral and transcriptome profiling of heterozygous Rab10 knock-out mice. eNeuro 10:ENEURO.0459-22.2023. 10.1523/ENEURO.0459-22.2023 PMC10208283 37156612

[B5] Calin-Jageman RJ, Cumming G (2019) Estimation for better inference in neuroscience. eNeuro 6:ENEURO.0205-19.2019. 10.1523/ENEURO.0205-19.2019 PMC6709209 31453316

[B6] Chakrabarty P, Ceballos-Diaz C, Beccard A, Janus C, Dickson D, Golde TE, Das P (2010) IFN-gamma promotes complement expression and attenuates amyloid plaque deposition in amyloid beta precursor protein transgenic mice. J Immunol 184:5333–5343. 10.4049/jimmunol.0903382 20368278 PMC3798002

[B7] Colgan LA, Hu M, Misler JA, Parra-Bueno P, Moran CM, Leitges M, Yasuda R (2018) PKCalpha integrates spatiotemporally distinct Ca(2+) and autocrine BDNF signaling to facilitate synaptic plasticity. Nat Neurosci 21:1027–1037. 10.1038/s41593-018-0184-3 30013171 PMC6100743

[B8] Dawson TM, Golde TE, Lagier-Tourenne C (2018) Animal models of neurodegenerative diseases. Nat Neurosci 21:1370–1379. 10.1038/s41593-018-0236-8 30250265 PMC6615039

[B9] De Strooper B, Karran E (2016) The cellular phase of Alzheimer's disease. Cell 164:603–615. 10.1016/j.cell.2015.12.056 26871627

[B10] Dorostkar MM, Zou C, Blazquez-Llorca L, Herms J (2015) Analyzing dendritic spine pathology in Alzheimer's disease: problems and opportunities. Acta Neuropathol 130:1–19. 10.1007/s00401-015-1449-5 26063233 PMC4469300

[B11] Hammonds-Odie LP, Jackson TR, Profit AA, Blader IJ, Turck CW, Prestwich GD, Theibert AB (1996) Identification and cloning of centaurin-alpha: a novel phosphatidylinositol 3,4,5-trisphosphate-binding protein from rat brain. J Biol Chem 271:18859–18868. 10.1074/jbc.271.31.18859 8702546 PMC4298166

[B12] Hayashi H, Matsuzaki O, Muramatsu S, Tsuchiya Y, Harada T, Suzuki Y, Sugano S, Matsuda A, Nishida E (2006) Centaurin-alpha1 is a phosphatidylinositol 3-kinase-dependent activator of ERK1/2 mitogen-activated protein kinases. J Biol Chem 281:1332–1337. 10.1074/jbc.M505905200 16287813

[B13] Ho J, Tumkaya T, Aryal S, Choi H, Claridge-Chang A (2019) Moving beyond P values: data analysis with estimation graphics. Nat Methods 16:565–566. 10.1038/s41592-019-0470-3 31217592

[B14] Jacobsen JS, et al. (2006) Early-onset behavioral and synaptic deficits in a mouse model of Alzheimer's disease. Proc Natl Acad Sci U S A 103:5161–5166. 10.1073/pnas.0600948103 16549764 PMC1405622

[B15] Javonillo DI, et al. (2021) Systematic phenotyping and characterization of the 3xTg-AD mouse model of Alzheimer's disease. Front Neurosci 15:785276. 10.3389/fnins.2021.785276 35140584 PMC8818877

[B16] Kirouac L, Rajic AJ, Cribbs DH, Padmanabhan J (2017) Activation of Ras-ERK signaling and GSK-3 by amyloid precursor protein and amyloid beta facilitates neurodegeneration in Alzheimer's disease. eNeuro 4:ENEURO.0149-16.2017. 10.1523/ENEURO.0149-16.2017 PMC5367084 28374012

[B17] Kommaddi RP, Das D, Karunakaran S, Nanguneri S, Bapat D, Ray A, Shaw E, Bennett DA, Nair D, Ravindranath V (2018) Abeta mediates F-actin disassembly in dendritic spines leading to cognitive deficits in Alzheimer's disease. J Neurosci 38:1085–1099. 10.1523/JNEUROSCI.2127-17.2017 29246925 PMC5792472

[B18] Kreutz MR, Bockers TM, Sabel BA, Hulser E, Stricker R, Reiser G (1997) Expression and subcellular localization of p42IP4/centaurin-alpha, a brain-specific, high-affinity receptor for inositol 1,3,4,5-tetrakisphosphate and phosphatidylinositol 3,4,5-trisphosphate in rat brain. Eur J Neurosci 9:2110–2124. 10.1111/j.1460-9568.1997.tb01378.x 9421171

[B19] Lee A, et al. (2022) Abeta42 oligomers trigger synaptic loss through CAMKK2-AMPK-dependent effectors coordinating mitochondrial fission and mitophagy. Nat Commun 13:4444. 10.1038/s41467-022-32130-5 35915085 PMC9343354

[B20] Li S, Jin M, Koeglsperger T, Shepardson NE, Shankar GM, Selkoe DJ (2011) Soluble Abeta oligomers inhibit long-term potentiation through a mechanism involving excessive activation of extrasynaptic NR2B-containing NMDA receptors. J Neurosci 31:6627–6638. 10.1523/JNEUROSCI.0203-11.2011 21543591 PMC3100898

[B21] Long JM, Holtzman DM (2019) Alzheimer disease: an update on pathobiology and treatment strategies. Cell 179:312–339. 10.1016/j.cell.2019.09.001 31564456 PMC6778042

[B22] Lopez-Lee C, et al. (2024a) Tlr7 drives sex differences in age- and Alzheimer's disease-related demyelination. Science 386:eadk7844. 10.1126/science.adk7844 39607927 PMC12396121

[B23] Lopez-Lee C, Torres ERS, Carling G, Gan L (2024b) Mechanisms of sex differences in Alzheimer's disease. Neuron 112:1208–1221. 10.1016/j.neuron.2024.01.024 38402606 PMC11076015

[B24] Ma T, Trinh MA, Wexler AJ, Bourbon C, Gatti E, Pierre P, Cavener DR, Klann E (2013) Suppression of eIF2alpha kinases alleviates Alzheimer's disease-related plasticity and memory deficits. Nat Neurosci 16:1299–1305. 10.1038/nn.3486 23933749 PMC3756900

[B25] Mairet-Coello G, Courchet J, Pieraut S, Courchet V, Maximov A, Polleux F (2013) The CAMKK2-AMPK kinase pathway mediates the synaptotoxic effects of Abeta oligomers through Tau phosphorylation. Neuron 78:94–108. 10.1016/j.neuron.2013.02.003 23583109 PMC3784324

[B26] Manno FAM, Isla AG, Manno SHC, Ahmed I, Cheng SH, Barrios FA, Lau C (2019) Early stage alterations in white matter and decreased functional interhemispheric hippocampal connectivity in the 3xTg mouse model of Alzheimer's disease. Front Aging Neurosci 11:39. 10.3389/fnagi.2019.00039 30967770 PMC6440287

[B27] McKhann GM, et al. (2011) The diagnosis of dementia due to Alzheimer's disease: recommendations from the national institute on aging-Alzheimer's association workgroups on diagnostic guidelines for Alzheimer's disease. Alzheimers Dement 7:263–269. 10.1016/j.jalz.2011.03.005 21514250 PMC3312024

[B28] Mijalkov M, Volpe G, Fernaud-Espinosa I, DeFelipe J, Pereira JB, Merino-Serrais P (2021) Dendritic spines are lost in clusters in Alzheimer's disease. Sci Rep 11:12350. 10.1038/s41598-021-91726-x 34117272 PMC8196005

[B29] Moore CD, et al. (2007) The neuronal Arf GAP centaurin alpha1 modulates dendritic differentiation. J Cell Sci 120:2683–2693. 10.1242/jcs.006346 17635995 PMC2810648

[B30] Mucke L, Masliah E, Yu GQ, Mallory M, Rockenstein EM, Tatsuno G, Hu K, Kholodenko D, Johnson-Wood K, McConlogue L (2000) High-level neuronal expression of abeta 1-42 in wild-type human amyloid protein precursor transgenic mice: synaptotoxicity without plaque formation. J Neurosci 20:4050–4058. 10.1523/JNEUROSCI.20-11-04050.2000 10818140 PMC6772621

[B31] Palop JJ, et al. (2007) Aberrant excitatory neuronal activity and compensatory remodeling of inhibitory hippocampal circuits in mouse models of Alzheimer's disease. Neuron 55:697–711. 10.1016/j.neuron.2007.07.025 17785178 PMC8055171

[B32] Reiser G, Bernstein HG (2002) Neurons and plaques of Alzheimer's disease patients highly express the neuronal membrane docking protein p42IP4/centaurin alpha. Neuroreport 13:2417–2419. 10.1097/00001756-200212200-00008 12499840

[B33] Reiser G, Bernstein HG (2004) Altered expression of protein p42IP4/centaurin-alpha 1 in Alzheimer's disease brains and possible interaction of p42IP4 with nucleolin. Neuroreport 15:147–148. 10.1097/00001756-200401190-00028 15106847

[B34] Rosende-Roca M, et al. (2025) Exploring sex differences in Alzheimer's disease: a comprehensive analysis of a large patient cohort from a memory unit. Alzheimers Res Ther 17:27. 10.1186/s13195-024-01656-9 39844303 PMC11753069

[B35] Sedehizade F, Hanck T, Stricker R, Horstmayer A, Bernstein HG, Reiser G (2002) Cellular expression and subcellular localization of the human Ins(1,3,4,5)P(4)-binding protein, p42(IP4), in human brain and in neuronal cells. Brain Res Mol Brain Res 99:1–11. 10.1016/S0169-328X(01)00335-7 11869802

[B36] Sinnen BL, Bowen AB, Gibson ES, Kennedy MJ (2016) Local and use-dependent effects of beta-amyloid oligomers on NMDA receptor function revealed by optical quantal analysis. J Neurosci 36:11532–11543. 10.1523/JNEUROSCI.1603-16.2016 27911757 PMC5125218

[B37] Stricker R, Hulser E, Fischer J, Jarchau T, Walter U, Lottspeich F, Reiser G (1997) cDNA cloning of porcine p42IP4, a membrane-associated and cytosolic 42 kDa inositol(1,3,4,5)tetrakisphosphate receptor from pig brain with similarly high affinity for phosphatidylinositol (3,4,5)P3. FEBS Lett 405:229–236. 10.1016/S0014-5793(97)00188-9 9089296

[B38] Stricker R, Reiser G (2014) Functions of the neuron-specific protein ADAP1 (centaurin-alpha1) in neuronal differentiation and neurodegenerative diseases, with an overview of structural and biochemical properties of ADAP1. Biol Chem 395:1321–1340. 10.1515/hsz-2014-0107 24854535

[B39] Suberbielle E, Sanchez PE, Kravitz AV, Wang X, Ho K, Eilertson K, Devidze N, Kreitzer AC, Mucke L (2013) Physiologic brain activity causes DNA double-strand breaks in neurons, with exacerbation by amyloid-beta. Nat Neurosci 16:613–621. 10.1038/nn.3356 23525040 PMC3637871

[B40] Szatmari EM, Oliveira AF, Sumner EJ, Yasuda R (2013) Centaurin-alpha1-Ras-Elk-1 signaling at mitochondria mediates beta-amyloid-induced synaptic dysfunction. J Neurosci 33:5367–5374. 10.1523/JNEUROSCI.2641-12.2013 23516302 PMC3866502

[B41] Szatmari EM, Moran C, Cohen S, Jacob A, Parra-Bueno P, Kamasawa N, Guerrero-Given D, Klein M, Stackman R Jr, Yasuda R (2021) ADAP1/centaurin-alpha1 negatively regulates dendritic spine function and memory formation in the hippocampus. eNeuro 8:ENEURO.0111-20.2020. 10.1523/ENEURO.0111-20.2020 PMC7808333 33139322

[B42] Talantova M, et al. (2013) Abeta induces astrocytic glutamate release, extrasynaptic NMDA receptor activation, and synaptic loss. Proc Natl Acad Sci U S A 110:E2518–E2527. 10.1073/pnas.1306832110 23776240 PMC3704025

[B43] Thacker E, Kearns B, Chapman C, Hammond J, Howell A, Theibert A (2004) The arf6 GAP centaurin alpha-1 is a neuronal actin-binding protein which also functions via GAP-independent activity to regulate the actin cytoskeleton. Eur J Cell Biol 83:541–554. 10.1078/0171-9335-00416 15679100

[B44] van der Flier WM, de Vugt ME, Smets EMA, Blom M, Teunissen CE (2023) Towards a future where Alzheimer's disease pathology is stopped before the onset of dementia. Nat Aging 3:494–505. 10.1038/s43587-023-00404-2 37202515

[B45] Venkateswarlu K, Hanada T, Chishti AH (2005) Centaurin-alpha1 interacts directly with kinesin motor protein KIF13B. J Cell Sci 118:2471–2484. 10.1242/jcs.02369 15923660

[B46] Verret L, et al. (2012) Inhibitory interneuron deficit links altered network activity and cognitive dysfunction in Alzheimer model. Cell 149:708–721. 10.1016/j.cell.2012.02.046 22541439 PMC3375906

[B47] Wei W, Nguyen LN, Kessels HW, Hagiwara H, Sisodia S, Malinow R (2010) Amyloid beta from axons and dendrites reduces local spine number and plasticity. Nat Neurosci 13:190–196. 10.1038/nn.2476 20037574 PMC3310198

[B48] Wright AL, Zinn R, Hohensinn B, Konen LM, Beynon SB, Tan RP, Clark IA, Abdipranoto A, Vissel B (2013) Neuroinflammation and neuronal loss precede Abeta plaque deposition in the hAPP-J20 mouse model of Alzheimer's disease. PLoS One 8:e59586. 10.1371/journal.pone.0059586 23560052 PMC3613362

[B49] Yoshimura Y, Yamauchi Y, Shinkawa T, Taoka M, Donai H, Takahashi N, Isobe T, Yamauchi T (2004) Molecular constituents of the postsynaptic density fraction revealed by proteomic analysis using multidimensional liquid chromatography-tandem mass spectrometry. J Neurochem 88:759–768. 10.1046/j.1471-4159.2003.02136.x 14720225

[B50] Zhang L, Qin Z, Sharmin F, Lin W, Ricke KM, Zasloff MA, Stewart AFR, Chen HH (2021) Tyrosine phosphatase PTP1B impairs presynaptic NMDA receptor-mediated plasticity in a mouse model of Alzheimer's disease. Neurobiol Dis 156:105402. 10.1016/j.nbd.2021.105402 34044147

[B51] Zhang Y, et al. (2016) Purification and characterization of progenitor and mature human astrocytes reveals transcriptional and functional differences with mouse. Neuron 89:37–53. 10.1016/j.neuron.2015.11.013 26687838 PMC4707064

