# Supplementary material for: Lack of ADAP1/Centaurin-α1 Ameliorates Cognitive Impairment and Neuropathological Hallmarks in a Mouse Model of Alzheimer's Disease
Source: eNeuro. 2025 Nov 21;12(11):ENEURO.0063-25.2025. doi: 10.1523/ENEURO.0063-25.2025 (PMC12658313; doi:10.1523/ENEURO.0063-25.2025)
Supplement: Figure 1-1 — Biochemical validation of J20xKO mice: Full-length western blots showing the presence of transgene and lack of CentA1 immunoreactivity in the hippocampus of J20xKO mice at 12 months of age. β-Actin re-probing of the blots shows equal amount of protein samples loaded on the gels. The number of mice was 2/genotype. Download Figure 1, DOCX file. [file eneuro-12-ENEURO.0063-25.2025-s002.docx]

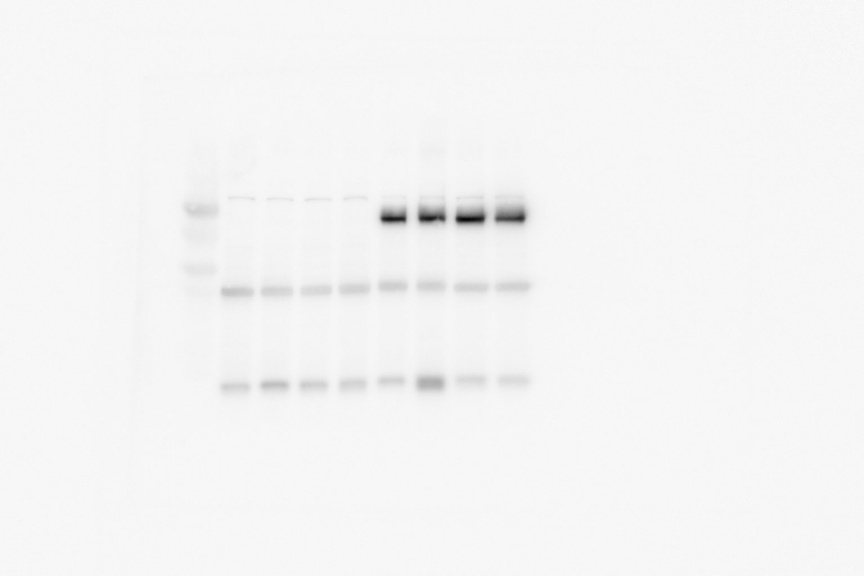

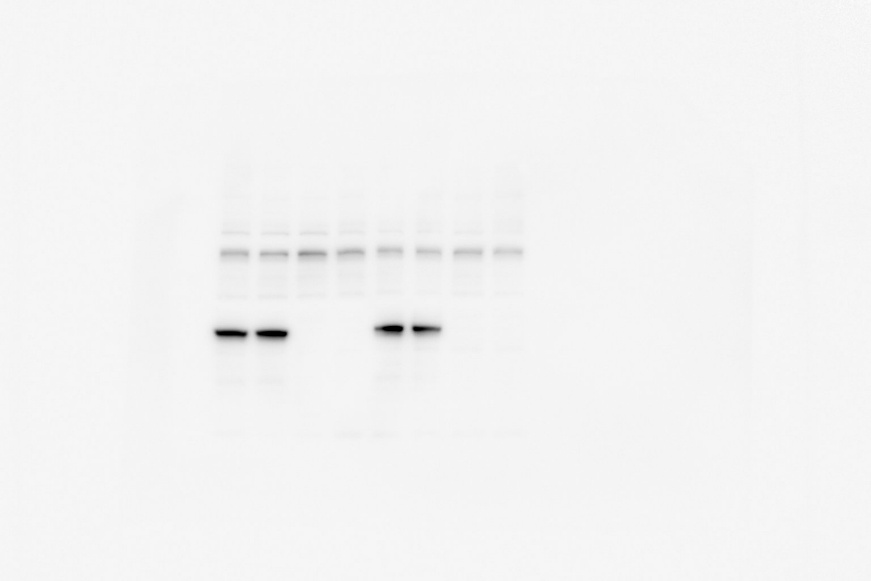

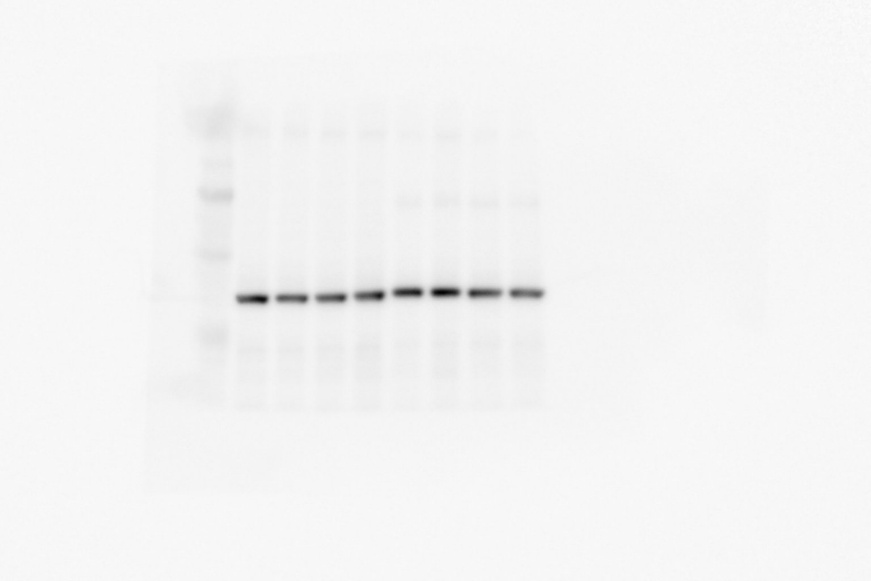


**WB:** CentA1/ADAP1

**Order of loading:**

Lane 1: Molecular Weight Marker

Lane 2-3: WT

Lane 4-5: KO

Lane 6-7: J20

Lane 8-9: J20xKO

**WB:** APP

**Order of loading:**

Lane 1: Molecular Weight Marker

Lane 2-3: WT

Lane 4-5: KO

Lane 6-7: J20

Lane 8-9: J20xKO

**WB:** beta-Actin

**Order of loading:**

Lane 1: Molecular Weight Marker

Lane 2-3: WT

Lane 4-5: KO

Lane 6-7: J20

Lane 8-9: J20xKO
