# Supplementary material for: Lack of ADAP1/Centaurin-α1 Ameliorates Cognitive Impairment and Neuropathological Hallmarks in a Mouse Model of Alzheimer's Disease
Source: eNeuro. 2025 Nov 21;12(11):ENEURO.0063-25.2025. doi: 10.1523/ENEURO.0063-25.2025 (PMC12658313; doi:10.1523/ENEURO.0063-25.2025)
Supplement: Figure 6-1 — Report of RNA integrity (RIN) scores. All RNA integrity (RIN) scores for the samples analyzed in Figure 6 were between 9.7–10. Download Figure 6, DOCX file. [file eneuro-12-ENEURO.0063-25.2025-s003.docx]

Assay Class: Data Path:

Eukaryote Total RNA Nano

C:\...Data\2020-11-09\Eukaryote Total RNA Nano_2020-11-09_001.xad

Created: Modified:

11/9/2020 11:43:24 AM

11/9/2020 12:07:15 PM

# Electrophoresis File Run Summary


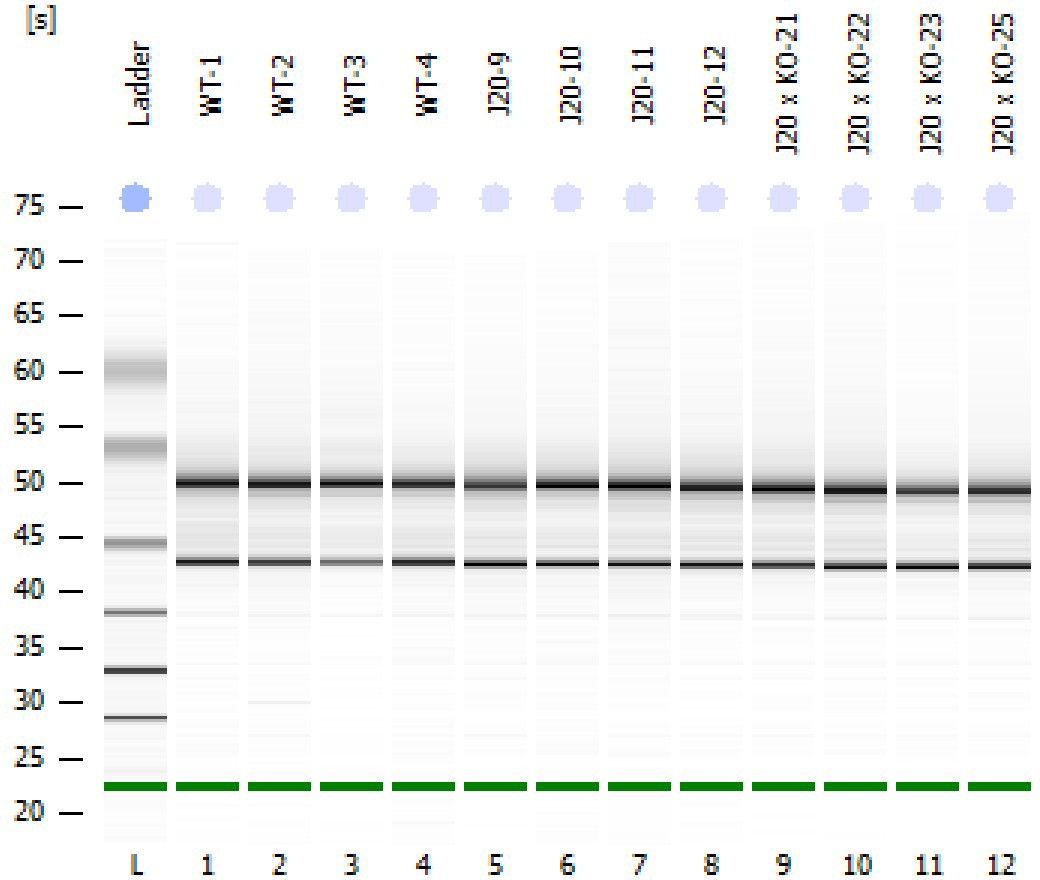
Instrument Information:

Instrument Name: DE13804527 Firmware: C.01.069

Serial#:

Assay Information:

DE13804527

Type: G2939A

Assay Origin Path: C:\Program Files\Agilent\2100 bioanalyzer\2100

expert\assays\RNA\Eukaryote Total RNA Nano Series II.xsy

Assay Class: Version:

Assay Comments:

Chip Information: Chip Lot #: Reagent Kit Lot #: Chip Comments:


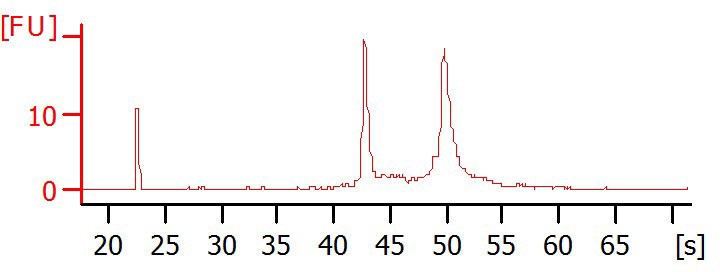

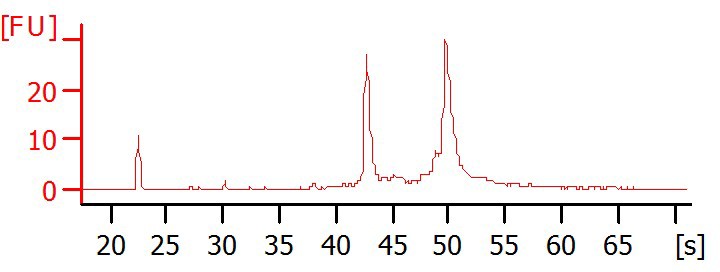

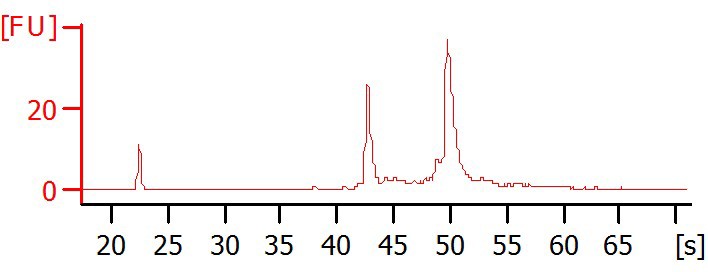


**WT-1**

**WT-2**

**WT-3**

RIN: 9.70 RIN: 9.80 RIN:10

Eukaryote Total RNA Nano 2.6

Total RNA Analysis ng sensitivity (Eukaryote)

© Copyright 2003 - 2009 Agilent Technologies, Inc.


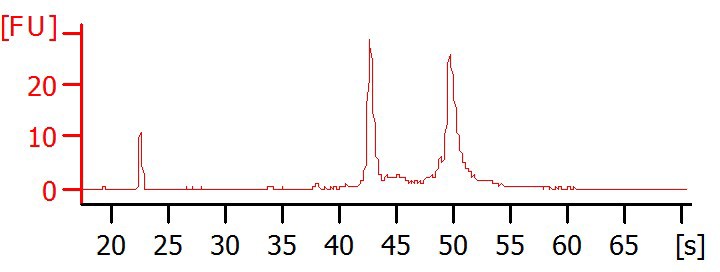

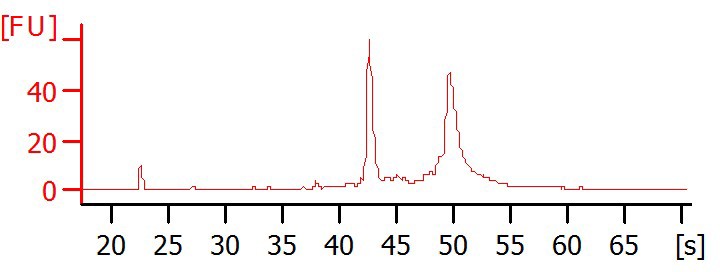

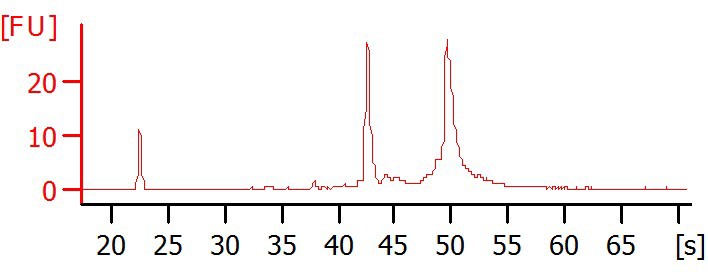


**WT-4**

**J20-9**

**J20-10**

RIN: 9.90 RIN: 9.70 RIN: 9.80


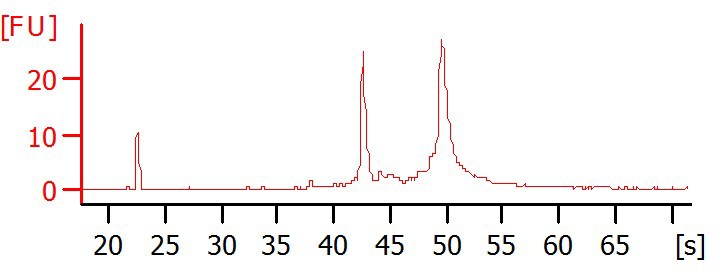

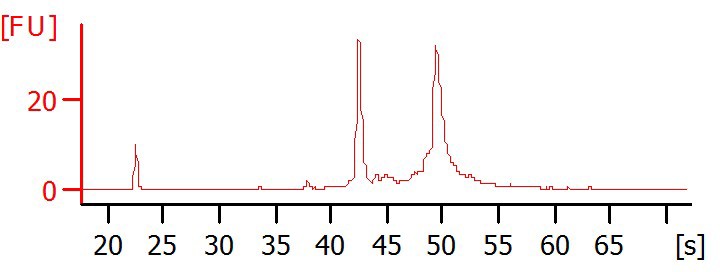

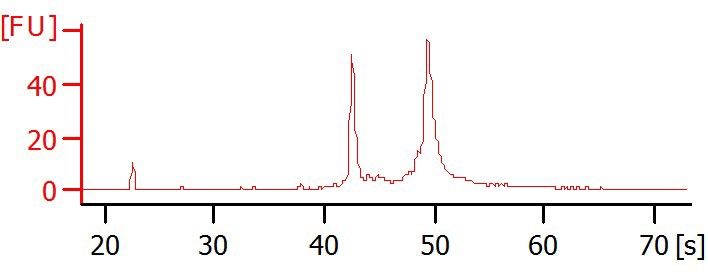


**J20-11**

**J20-12**

**J20 x KO-21**

RIN: 9.80 RIN: 9.80 RIN:10


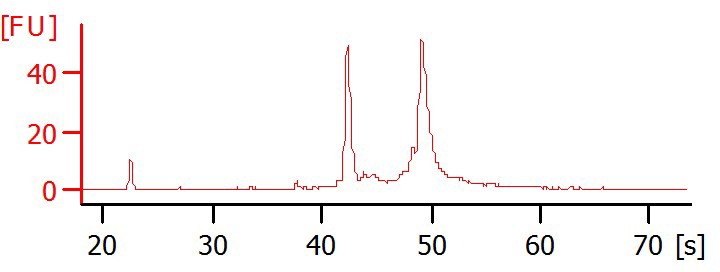

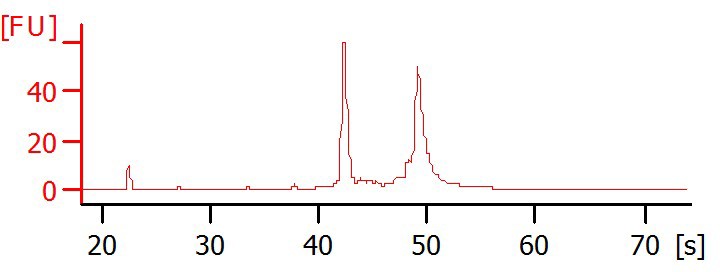

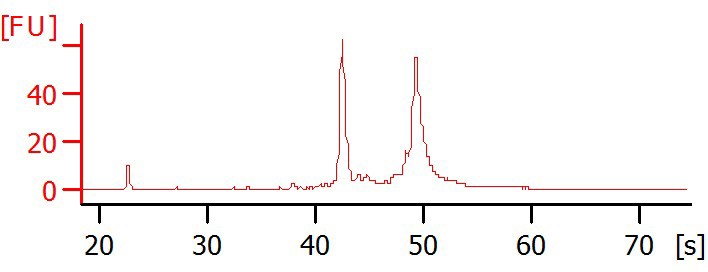


**J20 x KO-22**

**J20 x KO-23**

**J20 x KO-25**

RIN: 9.70 RIN: 9.80 RIN: 9.80

Assay Class: Data Path:

Eukaryote Total RNA Nano

C:\...Data\2020-11-09\Eukaryote Total RNA Nano_2020-11-09_001.xad

Created: Modified:

11/9/2020 11:43:24 AM

11/9/2020 12:07:15 PM

# Electrophoresis File Run Summary (Chip Summary)

**Sample Name Sample Comment Status Result Label Result Color**

WT-1 RIN: 9.70


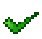

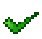

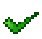

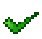

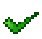

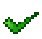

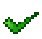

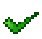

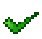

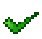

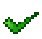

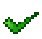

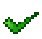


WT-2 RIN: 9.80

WT-3 RIN:10

WT-4 RIN: 9.90

J20-9 RIN: 9.70

J20-10 RIN: 9.80

J20-11 RIN: 9.80

J20-12 RIN: 9.80

J20 x KO-21 RIN:10

J20 x KO-22 RIN: 9.70

J20 x KO-23 RIN: 9.80

J20 x KO-25 RIN: 9.80

Ladder All Other Samples

**Chip Lot # Reagent Kit Lot #**

## Chip Comments :

Assay Class: Data Path:

Eukaryote Total RNA Nano

C:\...Data\2020-11-09\Eukaryote Total RNA Nano_2020-11-09_001.xad

Created: Modified:

11/9/2020 11:43:24 AM

11/9/2020 12:07:15 PM

# Electrophoresis Assay Details

## General Analysis Settings

Number of Available Sample and Ladder Wells (Max.) : 13 Minimum Visible Range [s] : 17

Maximum Visible Range [s] : 70 Start Analysis Time Range [s] : 19 End Analysis Time Range [s] : 69 Ladder Concentration [ng/µl] : 150

Lower Marker Concentration [ng/µl] : 0 Upper Marker Concentration [ng/µl] : 0 Used Lower Marker for Quantitation Standard Curve Fit is Logarithmic Show Data Aligned to Lower Marker

**Integrator Settings** Integration Start Time [s] : 19 Integration End Time [s] : 69

Slope Threshold : 0.6

Height Threshold [FU] : 0.5 Area Threshold : 0.2

Width Threshold [s] : 0.5 Baseline Plateau [s] : 6

| **Filter Settings** |  |
| --- | --- |
| Filter Width [s] : 0.5 Polynomial Order : 4 |  |
| **Ladder** |  |
| **Ladder Peak** | **Size** |
| 1 | 25 |
| 2 | 200 |
| 3 | 500 |
| 4 | 1000 |
| 5 | 2000 |
| 6 | 4000 |

Assay Class: Data Path:

Eukaryote Total RNA Nano

C:\...Data\2020-11-09\Eukaryote Total RNA Nano_2020-11-09_001.xad

Created: Modified:

11/9/2020 11:43:24 AM

11/9/2020 12:07:15 PM

# Electropherogram Summary


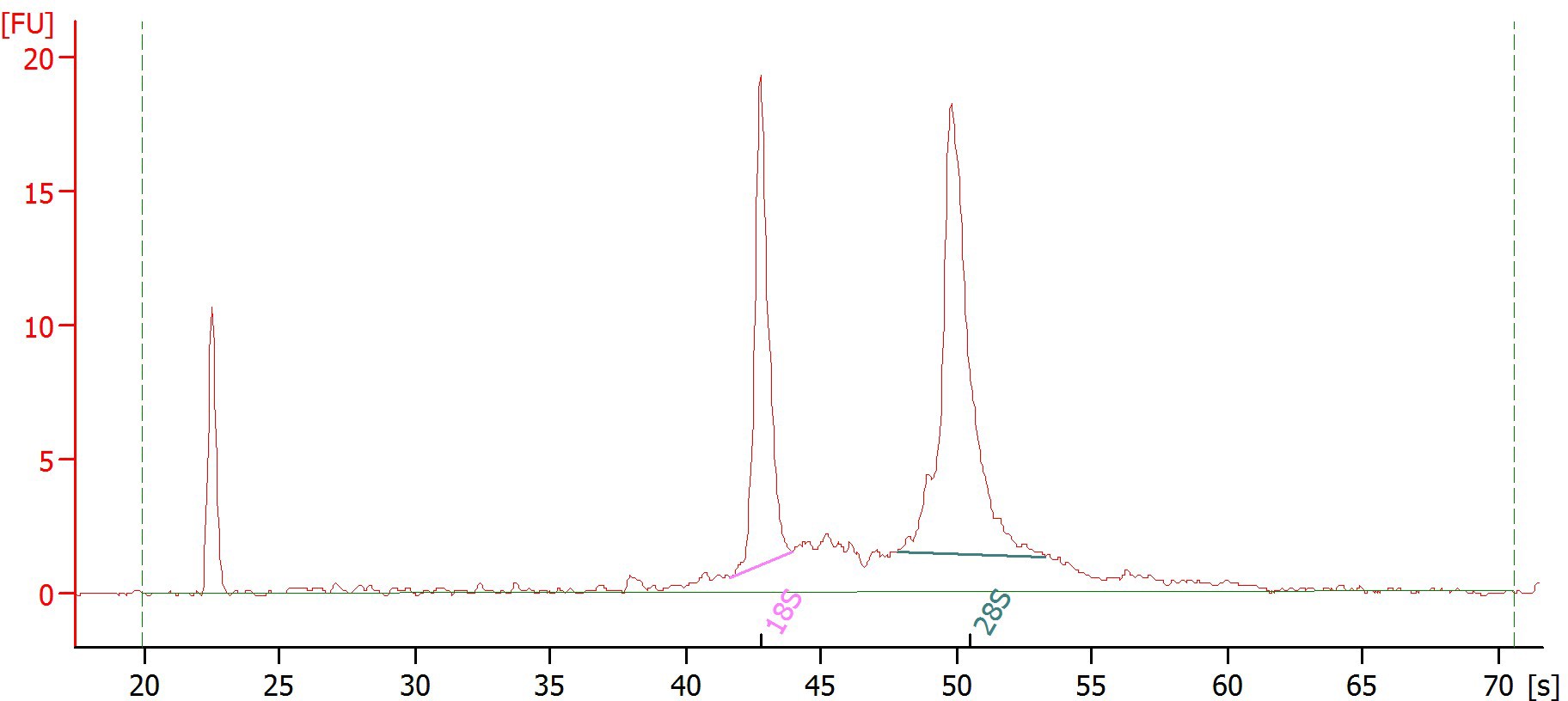
WT-1


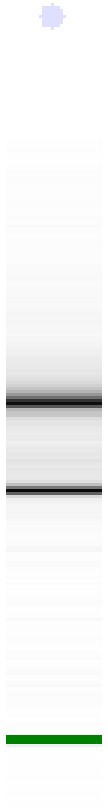


## Overall Results for sample 1 : WT-1

RNA Area: 117.1

RNA Concentration: 188 ng/µl

rRNA Ratio [28s / 18s]: 1.6

RNA Integrity Number (RIN): 9.7 (B.02.08) Result Flagging Color:

Result Flagging Label: RIN: 9.70

## Fragment table for sample 1 : WT-1

| **Name** | **Start Time [s]** | **End Time [s]** | **Area** | **% of total Area** |
| --- | --- | --- | --- | --- |
| 18S | 41.68 | 43.88 | 25.6 | 21.8 |
| 28S | 47.81 | 53.28 | 40.2 | 34.3 |

Assay Class: Data Path:

Eukaryote Total RNA Nano

C:\...Data\2020-11-09\Eukaryote Total RNA Nano_2020-11-09_001.xad

Created: Modified:

11/9/2020 11:43:24 AM

11/9/2020 12:07:15 PM

# Electropherogram Summary Continued ...


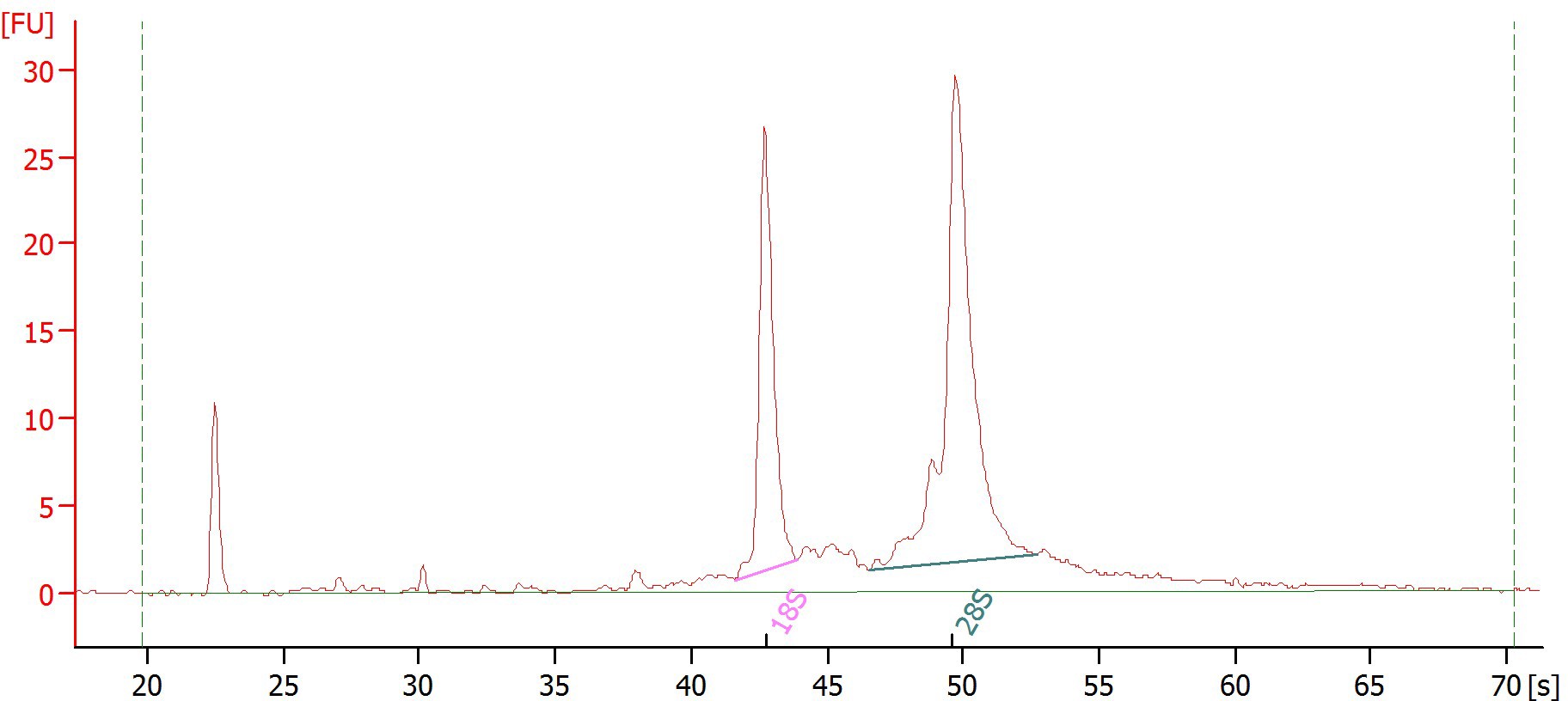
WT-2


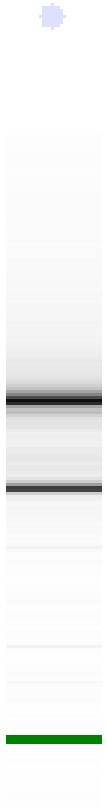


## Overall Results for sample 2 : WT-2

RNA Area: 170.1

RNA Concentration: 273 ng/µl

rRNA Ratio [28s / 18s]: 1.8

RNA Integrity Number (RIN): 9.8 (B.02.08) Result Flagging Color:

Result Flagging Label: RIN: 9.80

## Fragment table for sample 2 : WT-2

| **Name** | **Start Time [s]** | **End Time [s]** | **Area** | **% of total Area** |
| --- | --- | --- | --- | --- |
| 18S | 41.59 | 43.88 | 35.4 | 20.8 |
| 28S | 46.53 | 52.69 | 65.2 | 38.4 |

Assay Class: Data Path:

Eukaryote Total RNA Nano

C:\...Data\2020-11-09\Eukaryote Total RNA Nano_2020-11-09_001.xad

Created: Modified:

11/9/2020 11:43:24 AM

11/9/2020 12:07:15 PM

# Electropherogram Summary Continued ...


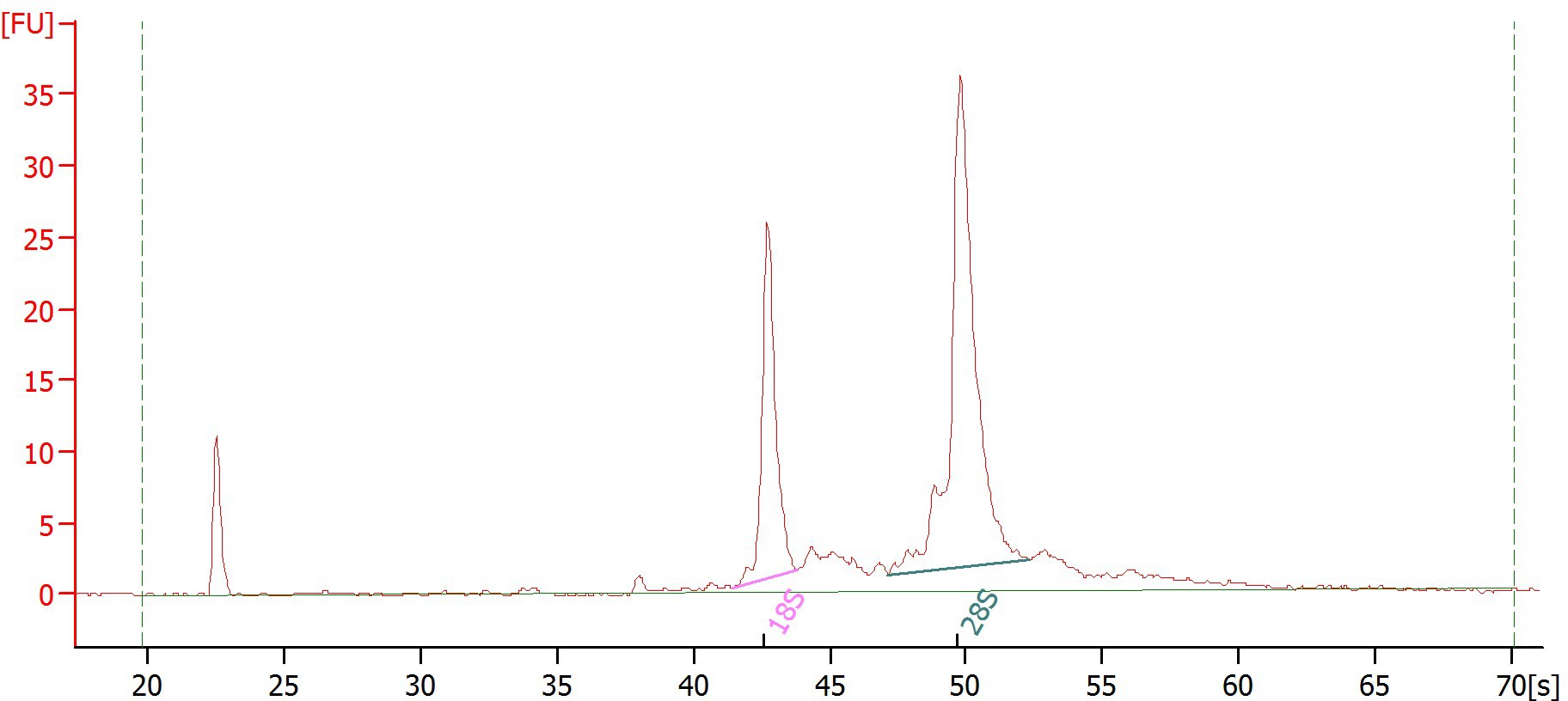
WT-3


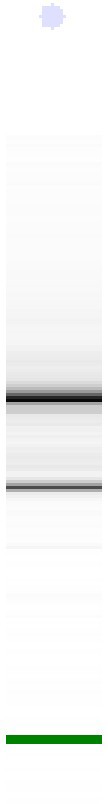


## Overall Results for sample 3 : WT-3

RNA Area: 163.3

RNA Concentration: 262 ng/µl

rRNA Ratio [28s / 18s]: 2.2

RNA Integrity Number (RIN): 10 (B.02.08) Result Flagging Color:

Result Flagging Label: RIN:10

## Fragment table for sample 3 : WT-3

| **Name** | **Start Time [s]** | **End Time [s]** | **Area** | **% of total Area** |
| --- | --- | --- | --- | --- |
| 18S | 41.44 | 43.78 | 32.5 | 19.9 |
| 28S | 47.13 | 52.36 | 71.1 | 43.5 |

Assay Class: Data Path:

Eukaryote Total RNA Nano

C:\...Data\2020-11-09\Eukaryote Total RNA Nano_2020-11-09_001.xad

Created: Modified:

11/9/2020 11:43:24 AM

11/9/2020 12:07:15 PM

# Electropherogram Summary Continued ...


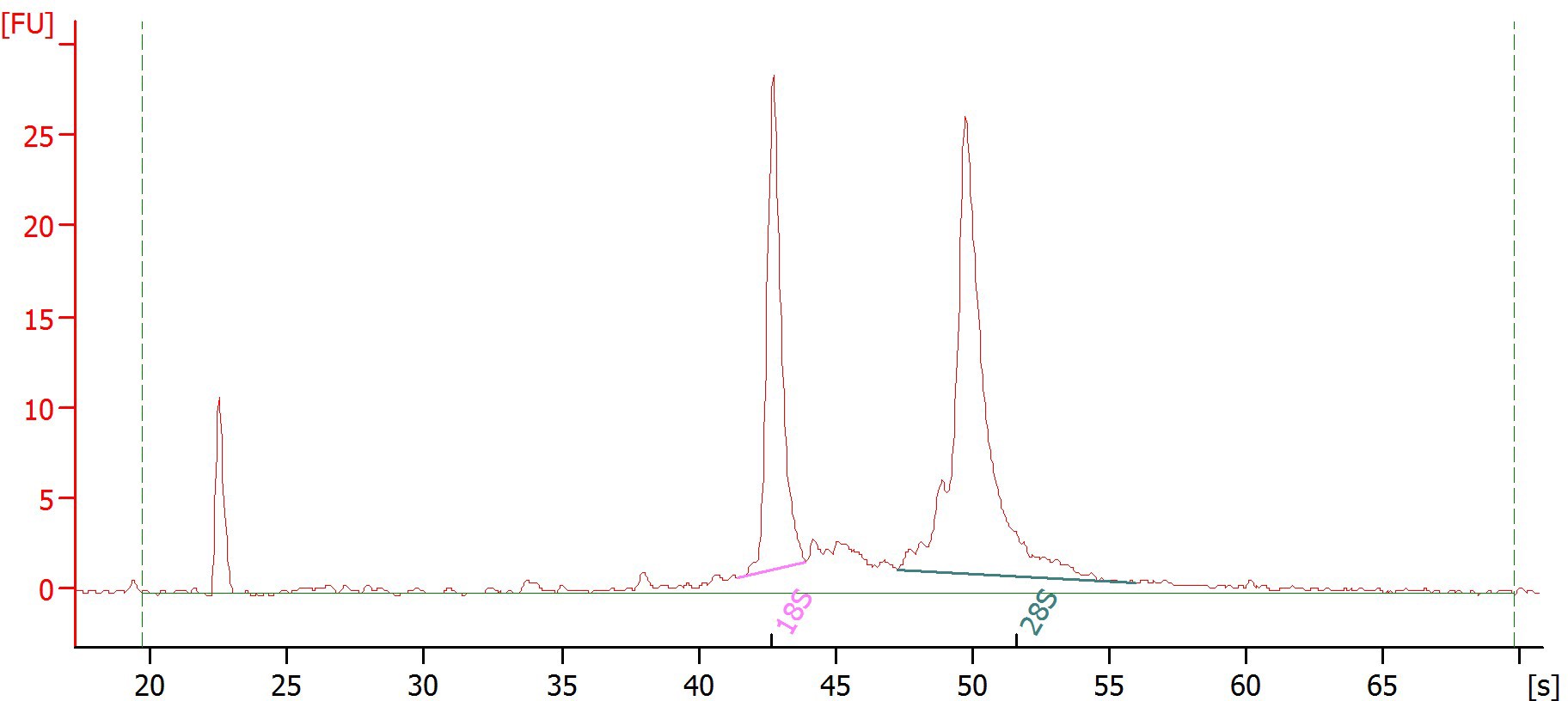
WT-4


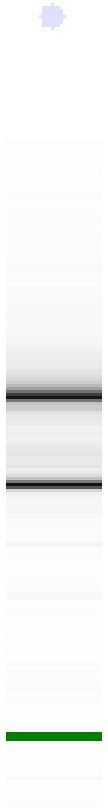


## Overall Results for sample 4 : WT-4

RNA Area: 156.4

RNA Concentration: 251 ng/µl

rRNA Ratio [28s / 18s]: 1.7

RNA Integrity Number (RIN): 9.9 (B.02.08) Result Flagging Color:

Result Flagging Label: RIN: 9.90

## Fragment table for sample 4 : WT-4

| **Name** | **Start Time [s]** | **End Time [s]** | **Area** | **% of total Area** |
| --- | --- | --- | --- | --- |
| 18S | 41.46 | 43.94 | 38.7 | 24.7 |
| 28S | 47.33 | 55.97 | 64.8 | 41.5 |

Assay Class: Data Path:

Eukaryote Total RNA Nano

C:\...Data\2020-11-09\Eukaryote Total RNA Nano_2020-11-09_001.xad

Created: Modified:

11/9/2020 11:43:24 AM

11/9/2020 12:07:15 PM

# Electropherogram Summary Continued ...


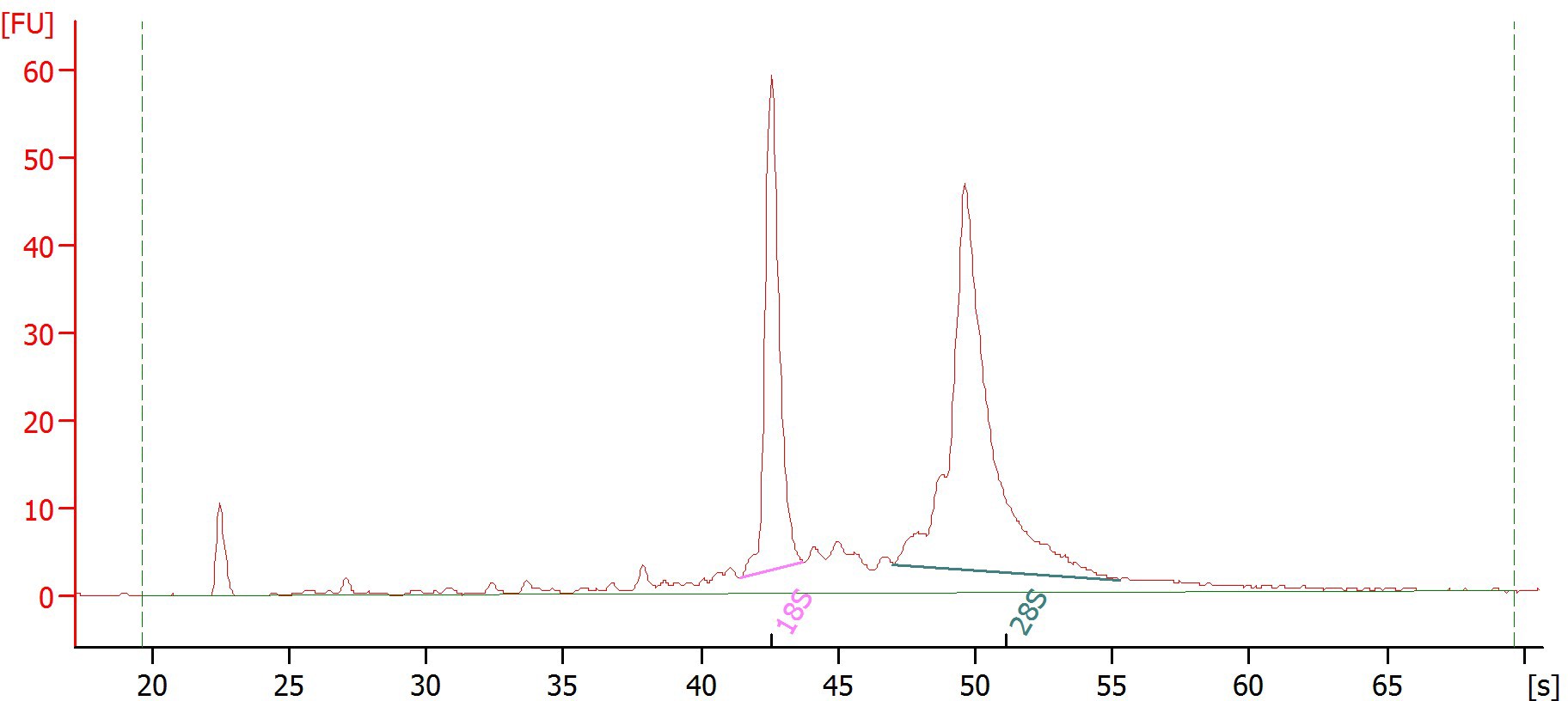
J20-9


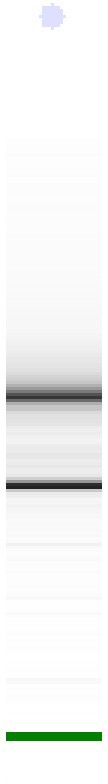


## Overall Results for sample 5 : J20-9

RNA Area: 342.2

RNA Concentration: 550 ng/µl

rRNA Ratio [28s / 18s]: 1.8

RNA Integrity Number (RIN): 9.7 (B.02.08) Result Flagging Color:

Result Flagging Label: RIN: 9.70

## Fragment table for sample 5 : J20-9

| **Name** | **Start Time [s]** | **End Time [s]** | **Area** | **% of total Area** |
| --- | --- | --- | --- | --- |
| 18S | 41.42 | 43.74 | 78.6 | 23.0 |
| 28S | 47.02 | 55.24 | 140.9 | 41.2 |

Assay Class: Data Path:

Eukaryote Total RNA Nano

C:\...Data\2020-11-09\Eukaryote Total RNA Nano_2020-11-09_001.xad

Created: Modified:

11/9/2020 11:43:24 AM

11/9/2020 12:07:15 PM

# Electropherogram Summary Continued ...


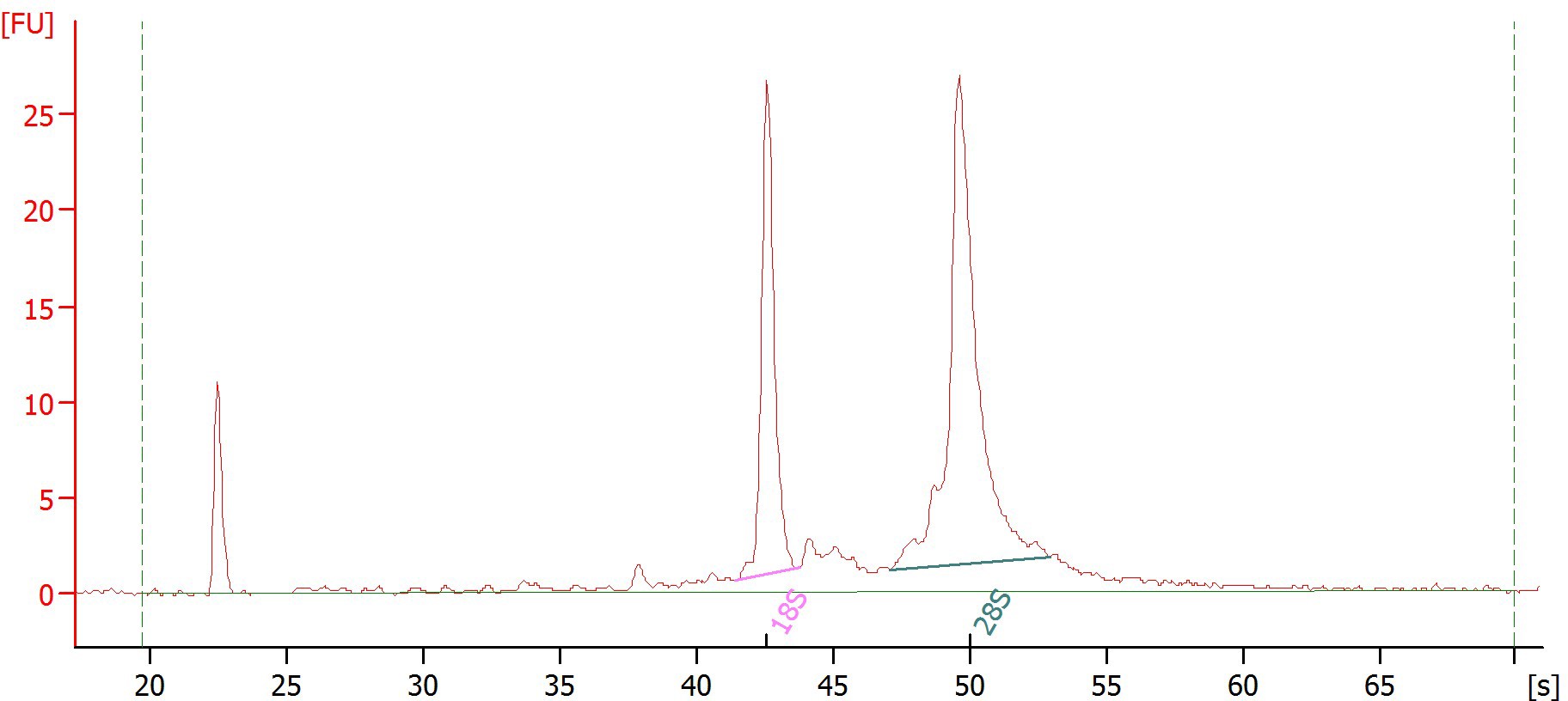
J20-10


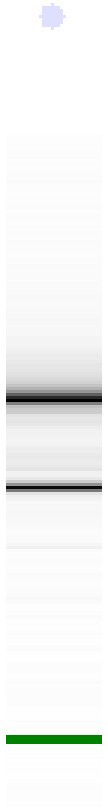


## Overall Results for sample 6 : J20-10

RNA Area: 148.5

RNA Concentration: 239 ng/µl

rRNA Ratio [28s / 18s]: 1.9

RNA Integrity Number (RIN): 9.8 (B.02.08) Result Flagging Color:

Result Flagging Label: RIN: 9.80

## Fragment table for sample 6 : J20-10

| **Name** | **Start Time [s]** | **End Time [s]** | **Area** | **% of total Area** |
| --- | --- | --- | --- | --- |
| 18S | 41.45 | 43.78 | 32.4 | 21.8 |
| 28S | 47.08 | 52.96 | 60.1 | 40.5 |

Assay Class: Data Path:

Eukaryote Total RNA Nano

C:\...Data\2020-11-09\Eukaryote Total RNA Nano_2020-11-09_001.xad

Created: Modified:

11/9/2020 11:43:24 AM

11/9/2020 12:07:15 PM

# Electropherogram Summary Continued ...


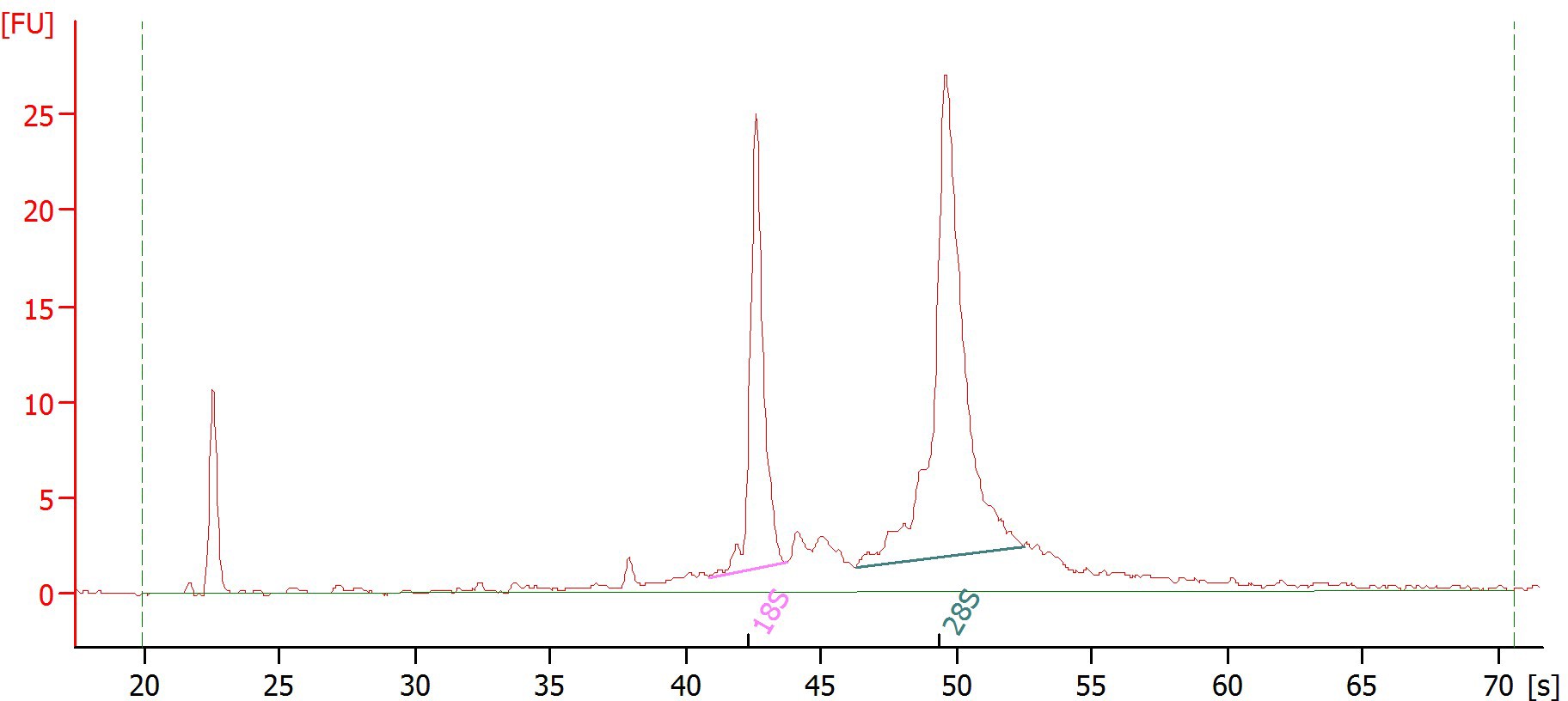
J20-11


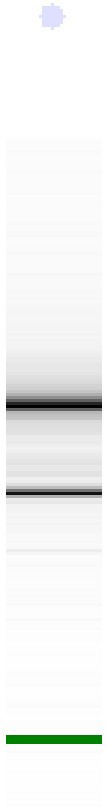


## Overall Results for sample 7 : J20-11

RNA Area: 167.3

RNA Concentration: 269 ng/µl

rRNA Ratio [28s / 18s]: 2.0

RNA Integrity Number (RIN): 9.8 (B.02.08) Result Flagging Color:

Result Flagging Label: RIN: 9.80

## Fragment table for sample 7 : J20-11

| **Name** | **Start Time [s]** | **End Time [s]** | **Area** | **% of total Area** |
| --- | --- | --- | --- | --- |
| 18S | 40.86 | 43.72 | 31.1 | 18.6 |
| 28S | 46.28 | 52.47 | 63.5 | 38.0 |

Assay Class: Data Path:

Eukaryote Total RNA Nano

C:\...Data\2020-11-09\Eukaryote Total RNA Nano_2020-11-09_001.xad

Created: Modified:

11/9/2020 11:43:24 AM

11/9/2020 12:07:15 PM

# Electropherogram Summary Continued ...


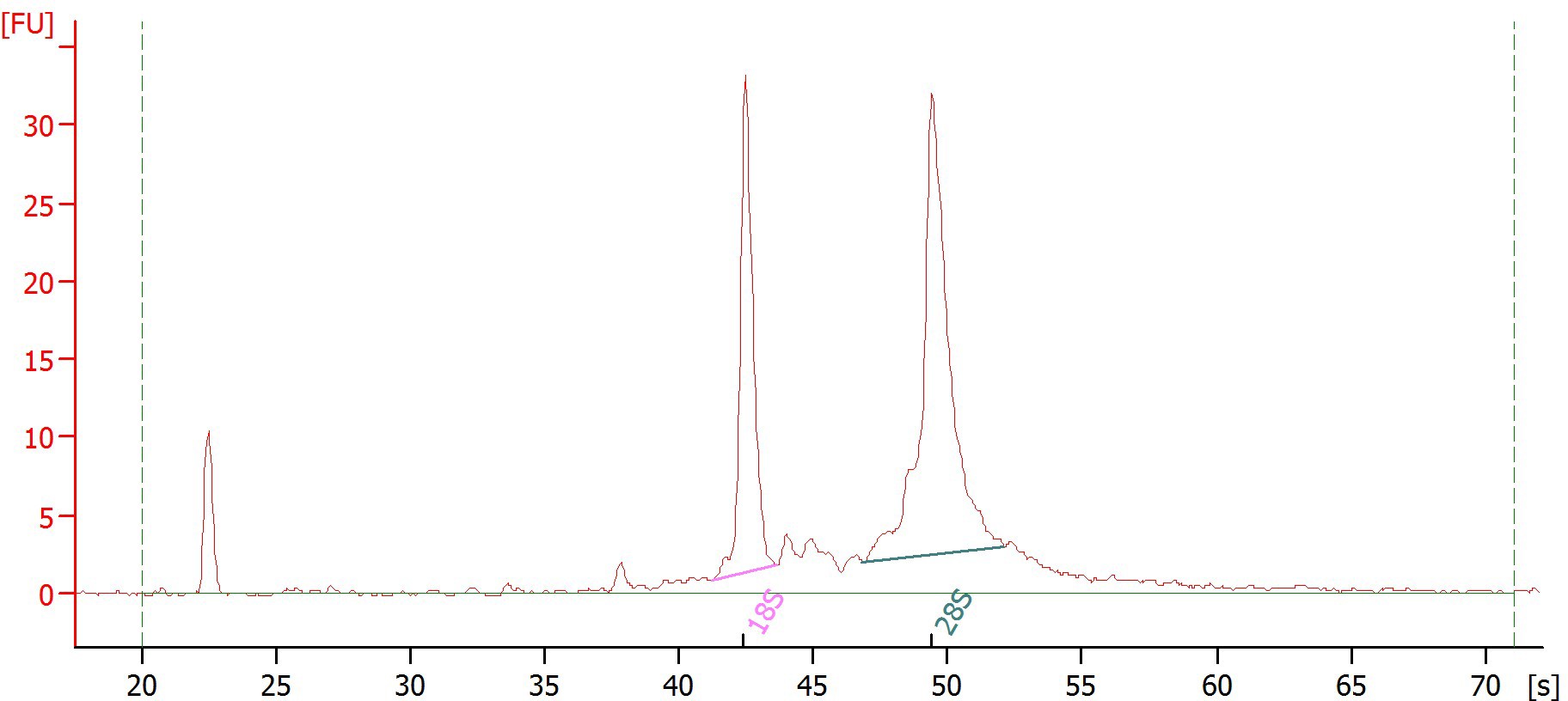
J20-12


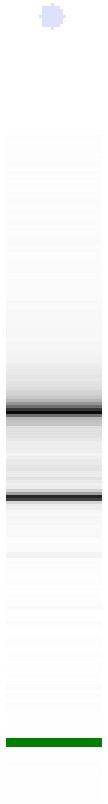


## Overall Results for sample 8 : J20-12

RNA Area: 194.5

RNA Concentration: 313 ng/µl

rRNA Ratio [28s / 18s]: 1.7

RNA Integrity Number (RIN): 9.8 (B.02.08) Result Flagging Color:

Result Flagging Label: RIN: 9.80

## Fragment table for sample 8 : J20-12

| **Name** | **Start Time [s]** | **End Time [s]** | **Area** | **% of total Area** |
| --- | --- | --- | --- | --- |
| 18S | 41.24 | 43.61 | 41.7 | 21.4 |
| 28S | 46.85 | 52.11 | 70.2 | 36.1 |

Assay Class: Data Path:

Eukaryote Total RNA Nano

C:\...Data\2020-11-09\Eukaryote Total RNA Nano_2020-11-09_001.xad

Created: Modified:

11/9/2020 11:43:24 AM

11/9/2020 12:07:15 PM

# Electropherogram Summary Continued ...


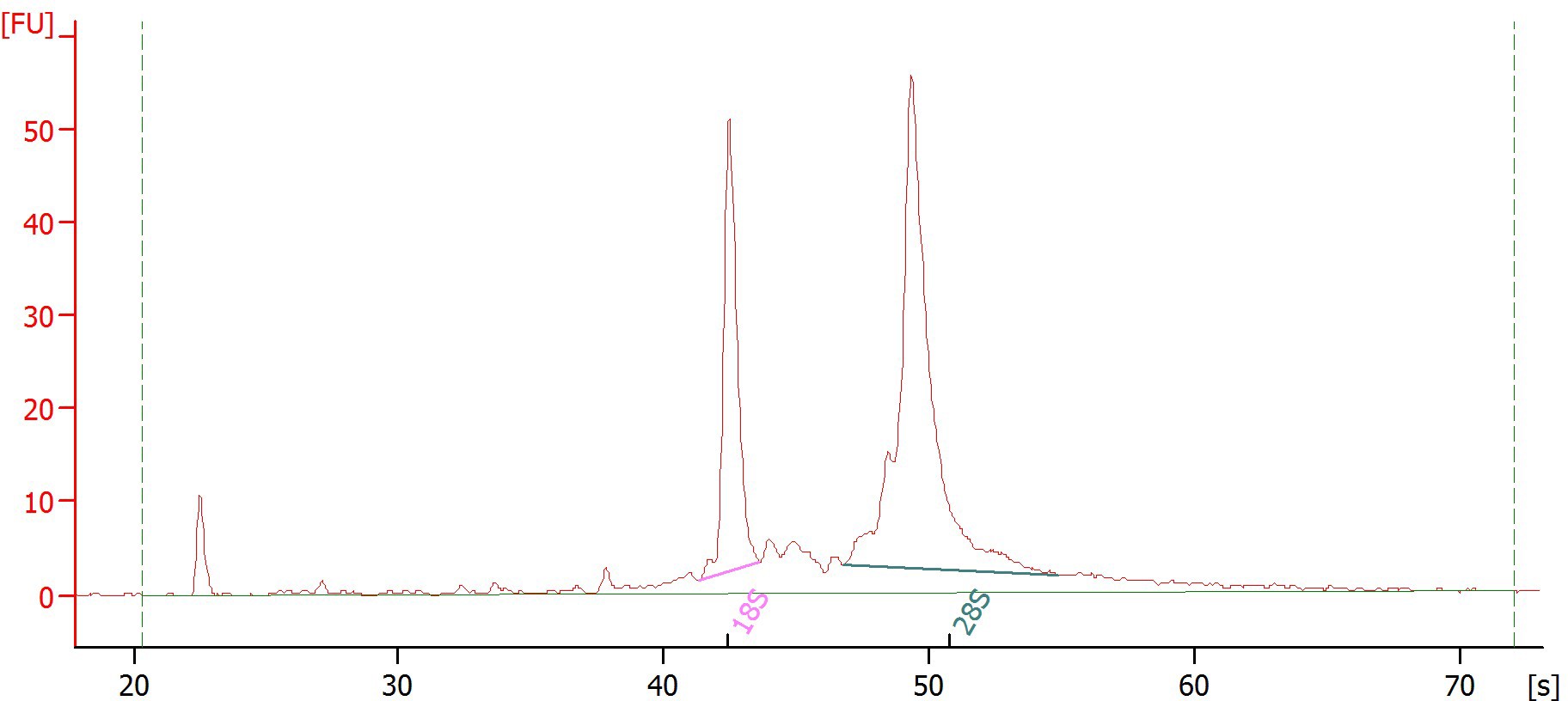
J20 x KO-21


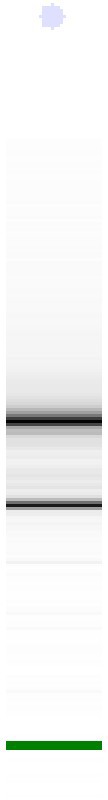


## Overall Results for sample 9 : J20 x KO-21

RNA Area: 328.4

RNA Concentration: 528 ng/µl

rRNA Ratio [28s / 18s]: 2.1

RNA Integrity Number (RIN): 10 (B.02.08) Result Flagging Color:

Result Flagging Label: RIN:10

## Fragment table for sample 9 : J20 x KO-21

| **Name** | **Start Time [s]** | **End Time [s]** | **Area** | **% of total Area** |
| --- | --- | --- | --- | --- |
| 18S | 41.29 | 43.59 | 68.4 | 20.8 |
| 28S | 46.77 | 54.87 | 144.0 | 43.9 |

Assay Class: Data Path:

Eukaryote Total RNA Nano

C:\...Data\2020-11-09\Eukaryote Total RNA Nano_2020-11-09_001.xad

Created: Modified:

11/9/2020 11:43:24 AM

11/9/2020 12:07:15 PM

# Electropherogram Summary Continued ...


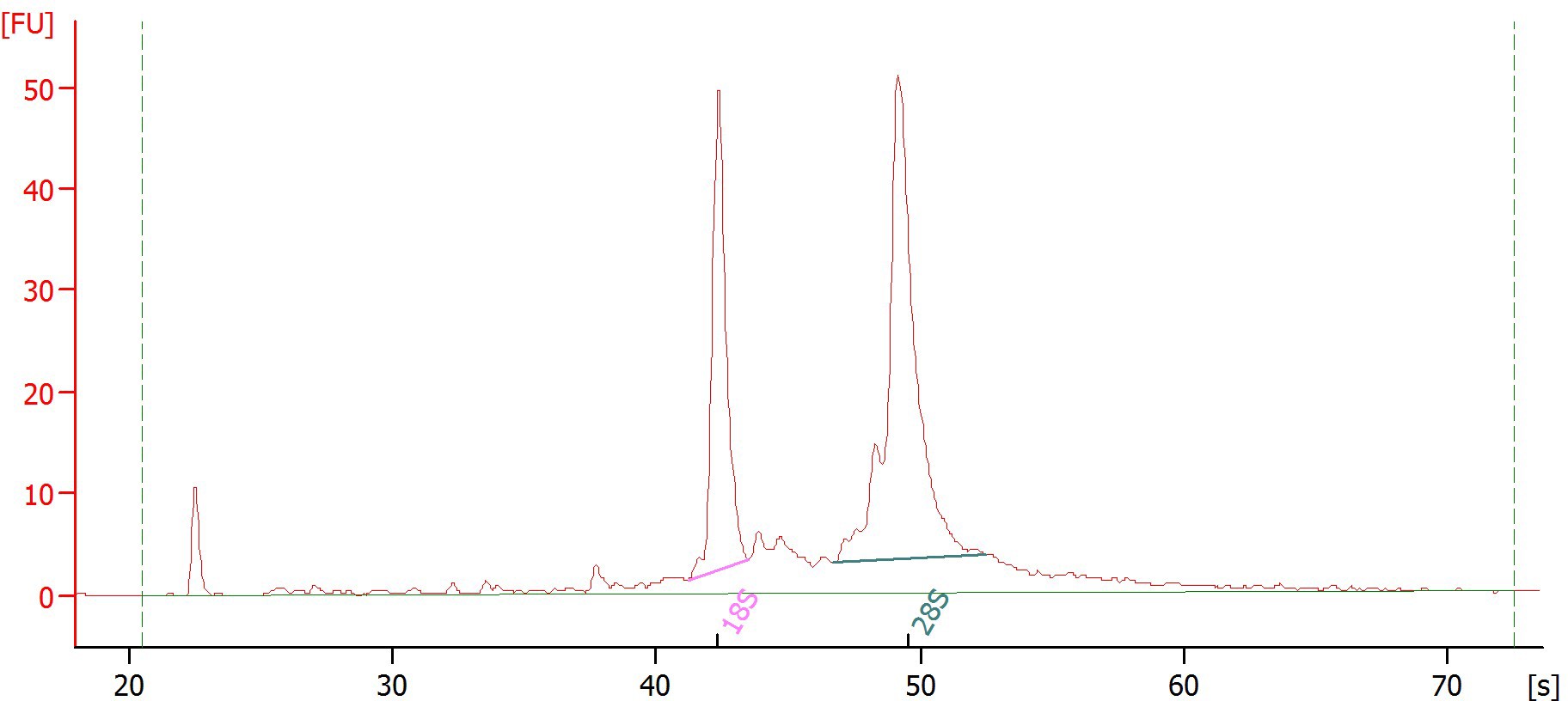
J20 x KO-22


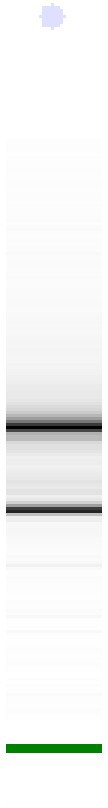


## Overall Results for sample 10 : J20 x KO-22

RNA Area: 320.3

RNA Concentration: 515 ng/µl

rRNA Ratio [28s / 18s]: 1.7

RNA Integrity Number (RIN): 9.7 (B.02.08) Result Flagging Color:

Result Flagging Label: RIN: 9.70

## Fragment table for sample 10 : J20 x KO-22

| **Name** | **Start Time [s]** | **End Time [s]** | **Area** | **% of total Area** |
| --- | --- | --- | --- | --- |
| 18S | 41.21 | 43.48 | 68.3 | 21.3 |
| 28S | 46.68 | 52.52 | 118.3 | 36.9 |

Assay Class: Data Path:

Eukaryote Total RNA Nano

C:\...Data\2020-11-09\Eukaryote Total RNA Nano_2020-11-09_001.xad

Created: Modified:

11/9/2020 11:43:24 AM

11/9/2020 12:07:15 PM

# Electropherogram Summary Continued ...


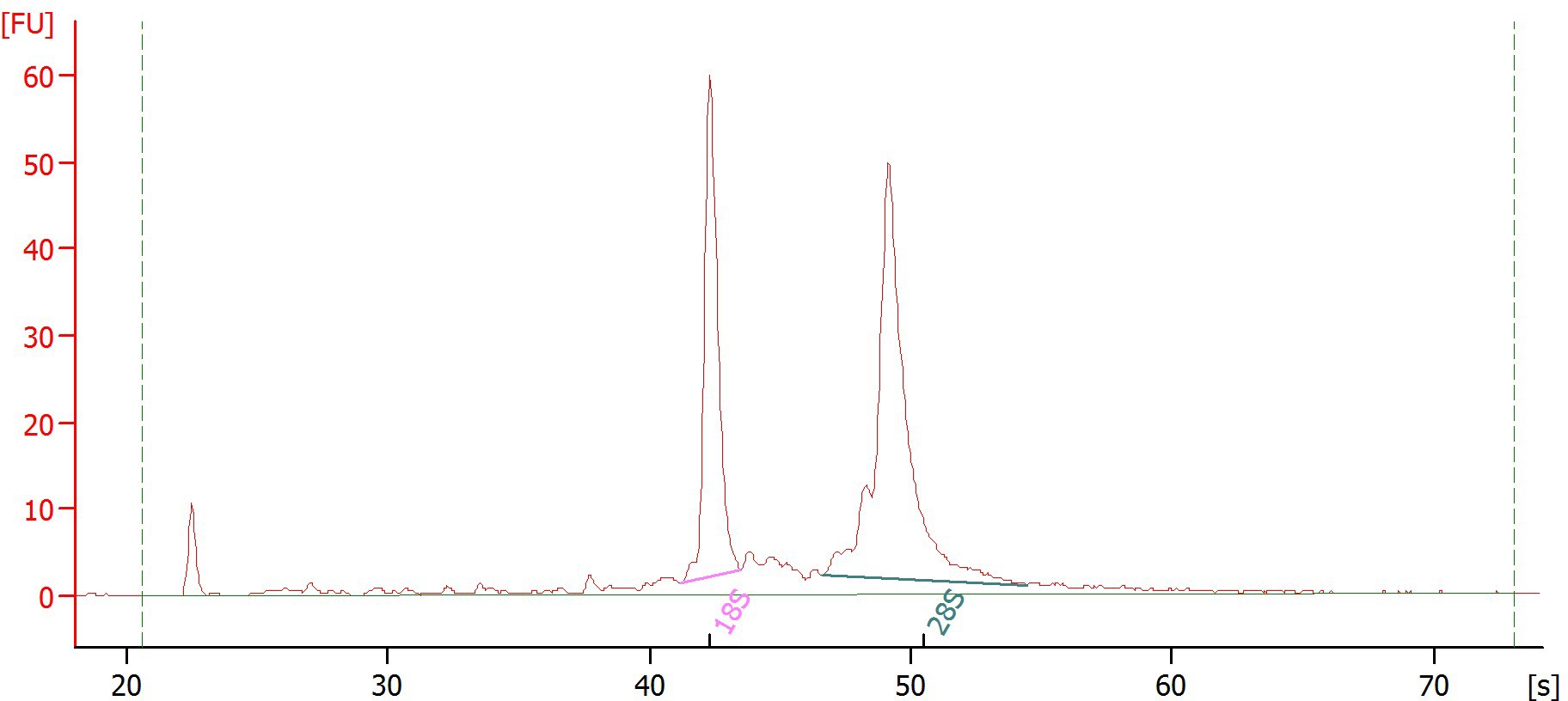
J20 x KO-23


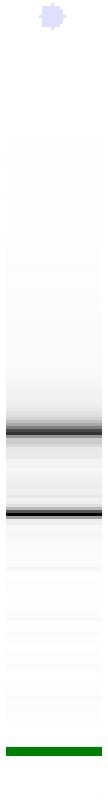


## Overall Results for sample 11 : J20 x KO-23

RNA Area: 314.3

RNA Concentration: 505 ng/µl

rRNA Ratio [28s / 18s]: 1.5

RNA Integrity Number (RIN): 9.8 (B.02.08) Result Flagging Color:

Result Flagging Label: RIN: 9.80

## Fragment table for sample 11 : J20 x KO-23

| **Name** | **Start Time [s]** | **End Time [s]** | **Area** | **% of total Area** |
| --- | --- | --- | --- | --- |
| 18S | 41.24 | 43.52 | 81.8 | 26.0 |
| 28S | 46.69 | 54.42 | 123.9 | 39.4 |

Assay Class: Data Path:

Eukaryote Total RNA Nano

C:\...Data\2020-11-09\Eukaryote Total RNA Nano_2020-11-09_001.xad

Created: Modified:

11/9/2020 11:43:24 AM

11/9/2020 12:07:15 PM

# Electropherogram Summary Continued ...


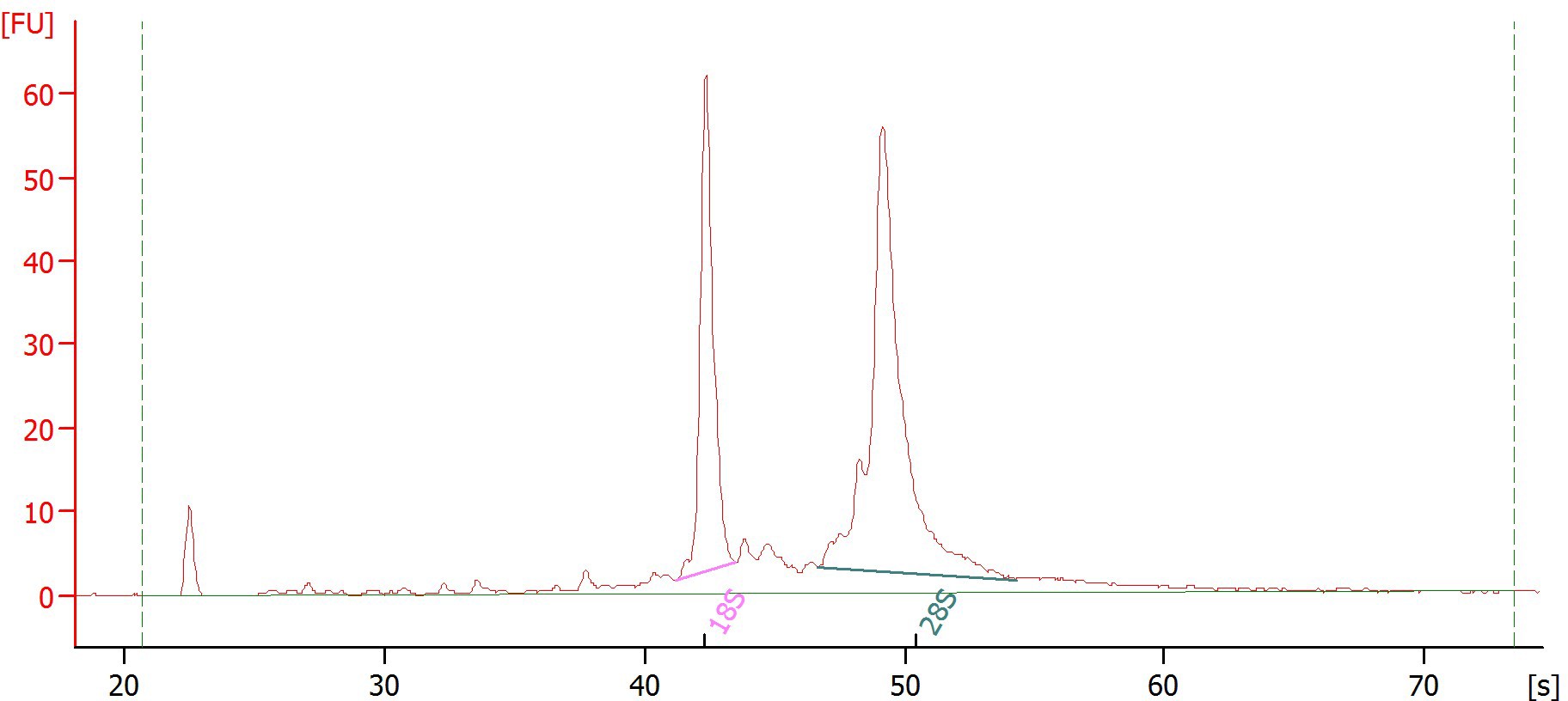
J20 x KO-25


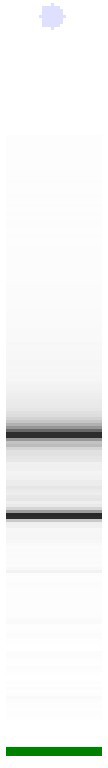


## Overall Results for sample 12 : J20 x KO-25

RNA Area: 354.5

RNA Concentration: 570 ng/µl

rRNA Ratio [28s / 18s]: 1.7

RNA Integrity Number (RIN): 9.8 (B.02.08) Result Flagging Color:

Result Flagging Label: RIN: 9.80

## Fragment table for sample 12 : J20 x KO-25

| **Name** | **Start Time [s]** | **End Time [s]** | **Area** | **% of total Area** |
| --- | --- | --- | --- | --- |
| 18S | 41.22 | 43.51 | 84.2 | 23.8 |
| 28S | 46.70 | 54.26 | 146.2 | 41.3 |

Assay Class: Data Path:

# Gel Image

Eukaryote Total RNA Nano

C:\...Data\2020-11-09\Eukaryote Total RNA Nano_2020-11-09_001.xad

Created: Modified:

11/9/2020 11:43:24 AM

11/9/2020 12:07:15 PM


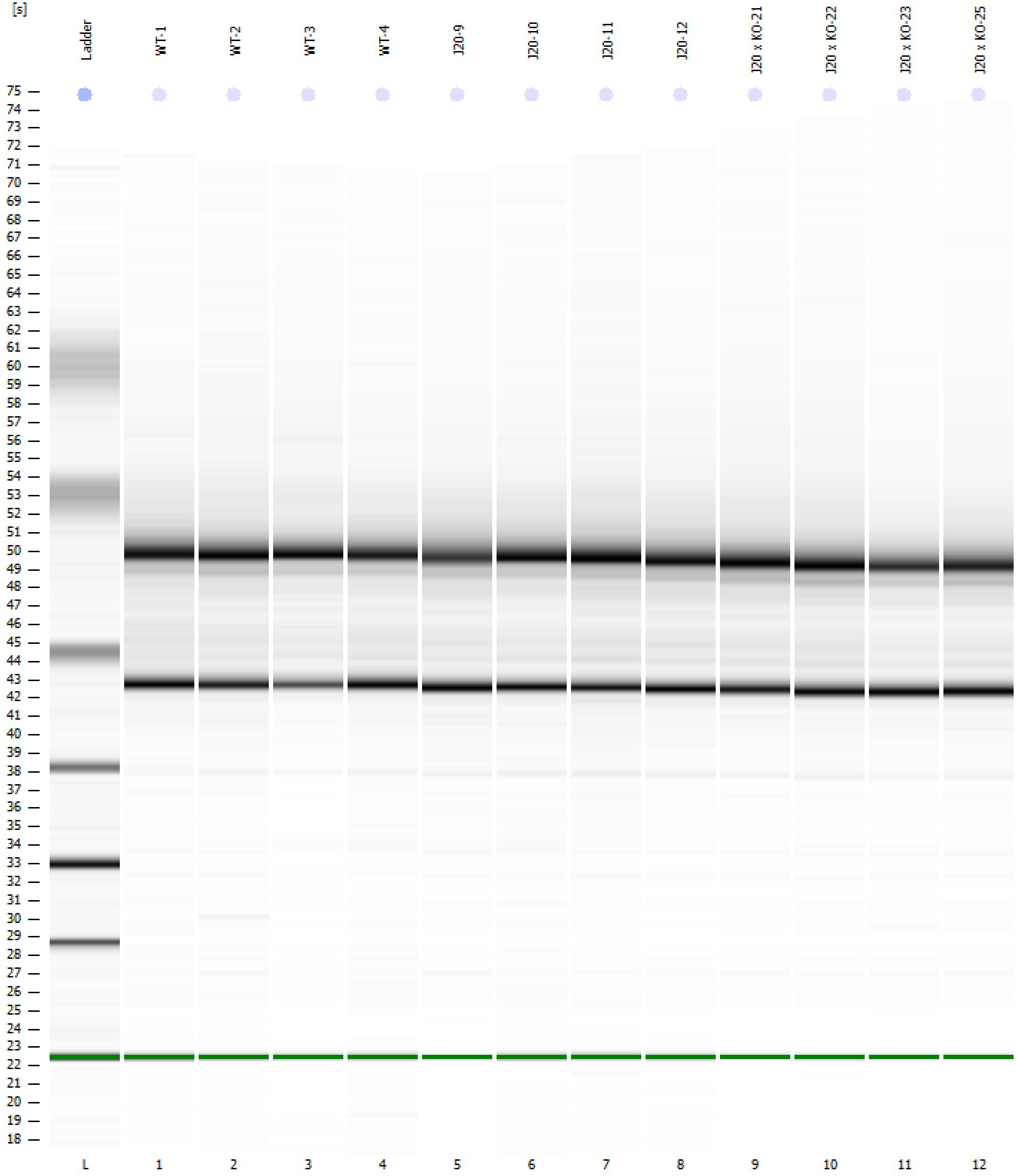


Assay Class: Data Path:

# Curves

Eukaryote Total RNA Nano

C:\...Data\2020-11-09\Eukaryote Total RNA Nano_2020-11-09_001.xad

Created: Modified:

11/9/2020 11:43:24 AM

11/9/2020 12:07:15 PM

**Standard Curve**

**
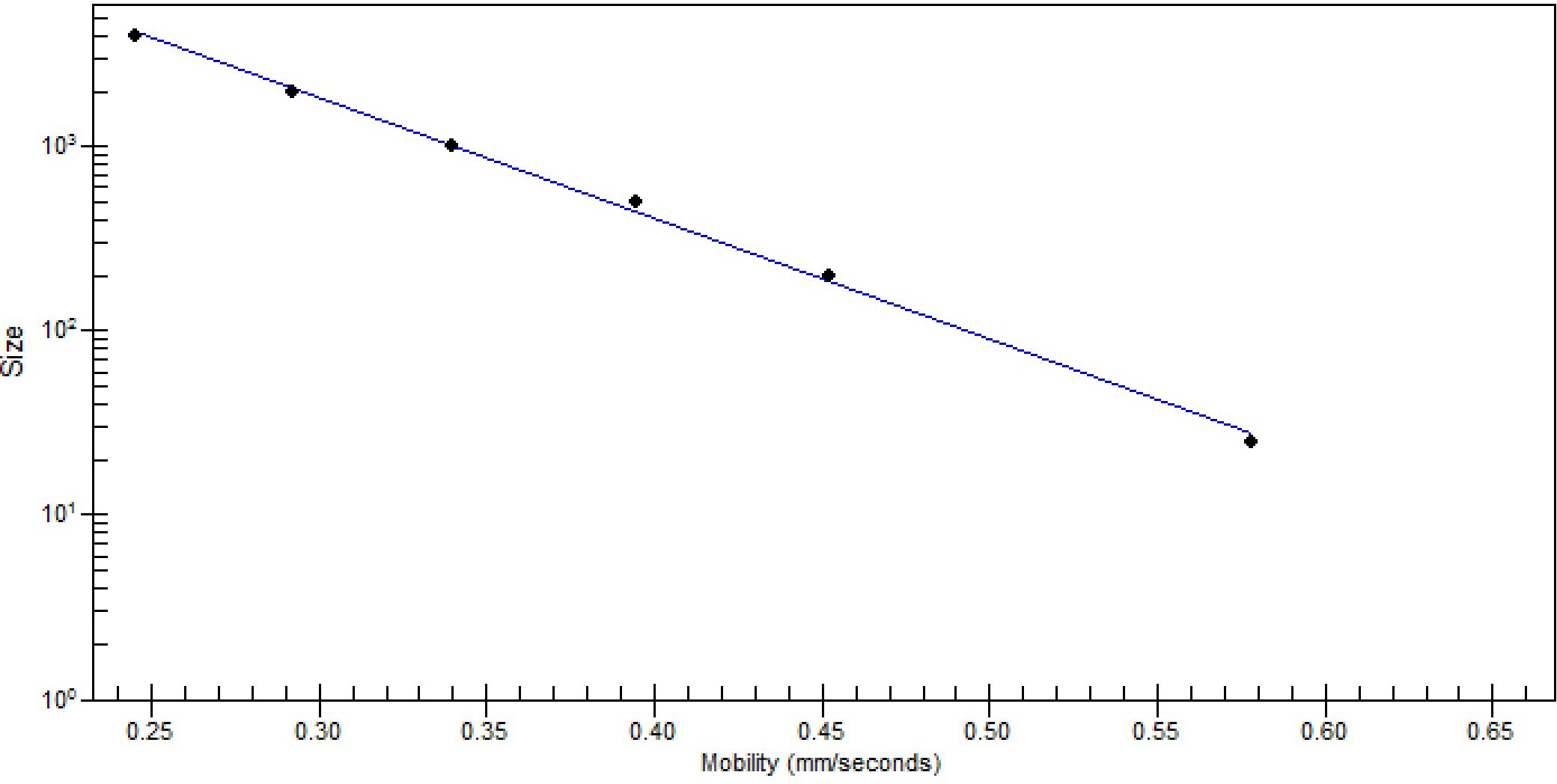
**
