# Supplementary material for: Lack of ADAP1/Centaurin-α1 Ameliorates Cognitive Impairment and Neuropathological Hallmarks in a Mouse Model of Alzheimer's Disease
Source: eNeuro. 2025 Nov 21;12(11):ENEURO.0063-25.2025. doi: 10.1523/ENEURO.0063-25.2025 (PMC12658313; doi:10.1523/ENEURO.0063-25.2025)
Supplement: Figure 6-2 — List of differentially expressed genes (DEGs) identified by NanoString nCounter gene expression profiling. Table shows all differentially expressed genes (DEGs) in the forebrain of J20, J20xKO mice and their wild-type littermates. Download Figure 6-2, DOCX file. [file eneuro-12-ENEURO.0063-25.2025-s004.docx]

|  | **J20 vs J20-KO** | | **WT vs J20** | | **WT vs J20-KO** | |
| --- | --- | --- | --- | --- | --- | --- |
| **Gene Name** | **P-value** | **Fold change** | **P-value** | **Fold change** | **P-value** | **Fold change** |
| **Bid** | 2.69E-04 | 7.95E-01 | 2.72E-01 | 1.07E+00 | 6.00E-02 | 8.53E-01 |
| **Rab3c** | 1.38E-03 | 8.45E-01 | 2.28E-03 | 1.11E+00 | 4.90E-02 | 9.41E-01 |
| **Ncam1** | 1.91E-03 | 8.96E-01 | 5.68E-02 | 1.06E+00 | 1.77E-01 | 9.52E-01 |
| **Nf1** | 1.98E-03 | 8.36E-01 | 5.62E-02 | 1.16E+00 | 7.03E-01 | 9.73E-01 |
| **Cntn4** | 2.27E-03 | 8.89E-01 | 1.57E-03 | 1.22E+00 | 7.44E-02 | 1.08E+00 |
| **Cntn1** | 2.49E-03 | 8.74E-01 | 1.41E-03 | 1.15E+00 | 8.99E-01 | 1.01E+00 |
| **Plxnc1** | 2.49E-03 | 7.55E-01 | 4.94E-02 | 1.20E+00 | 1.67E-01 | 9.06E-01 |
| **Opa1** | 2.58E-03 | 9.05E-01 | 7.84E-01 | 1.01E+00 | 1.74E-02 | 9.12E-01 |
| **Crebbp** | 2.87E-03 | 8.38E-01 | 1.83E-01 | 1.11E+00 | 3.48E-01 | 9.31E-01 |
| **Mtor** | 3.74E-03 | 8.64E-01 | 3.85E-03 | 1.15E+00 | 8.60E-01 | 9.92E-01 |
| **Grin2b** | 3.83E-03 | 7.14E-01 | 1.01E-03 | 1.32E+00 | 5.93E-01 | 9.45E-01 |
| **Adam10** | 4.11E-03 | 9.12E-01 | 5.67E-03 | 1.09E+00 | 8.94E-01 | 9.96E-01 |
| **Atp6v1g2** | 4.29E-03 | 9.34E-01 | 9.71E-01 | 1.00E+00 | 1.63E-01 | 9.36E-01 |
| **Tcerg1** | 4.37E-03 | 8.54E-01 | 1.94E-03 | 1.13E+00 | 3.89E-01 | 9.68E-01 |
| **Lrrc4** | 5.67E-03 | 9.24E-01 | 2.93E-01 | 9.63E-01 | 1.76E-02 | 8.90E-01 |
| **Tenm2** | 6.12E-03 | 8.04E-01 | 2.35E-02 | 1.11E+00 | 5.19E-02 | 8.96E-01 |
| **Nrxn1** | 6.12E-03 | 8.70E-01 | 4.58E-02 | 1.11E+00 | 4.90E-01 | 9.64E-01 |
| **Cacna1b** | 6.23E-03 | 7.33E-01 | 3.75E-02 | 1.24E+00 | 3.90E-01 | 9.08E-01 |
| **Cd8a** | 6.31E-03 | 5.78E-01 | 1.50E-02 | 1.53E+00 | 3.86E-01 | 8.82E-01 |
| **Mmp16** | 7.91E-03 | 9.10E-01 | 1.59E-01 | 1.09E+00 | 8.41E-01 | 9.88E-01 |
| **Stx1a** | 8.99E-03 | 8.91E-01 | 4.14E-01 | 1.06E+00 | 4.27E-01 | 9.43E-01 |
| **Grin2a** | 9.99E-03 | 7.95E-01 | 2.16E-01 | 1.08E+00 | 9.02E-02 | 8.61E-01 |
| **Scn1a** | 1.00E-02 | 8.69E-01 | 3.43E-01 | 1.05E+00 | 1.75E-01 | 9.16E-01 |
| **Npc1** | 1.03E-02 | 9.08E-01 | 1.39E-01 | 1.10E+00 | 9.26E-01 | 9.95E-01 |
| **Cnr1** | 1.06E-02 | 8.09E-01 | 9.85E-02 | 1.15E+00 | 4.50E-01 | 9.31E-01 |
| **Lpar1** | 1.08E-02 | 1.35E+00 | 3.01E-01 | 8.36E-01 | 4.42E-01 | 1.13E+00 |
| **Hcn1** | 1.11E-02 | 8.19E-01 | 2.51E-02 | 1.17E+00 | 4.00E-01 | 9.58E-01 |
| **Stambpl1** | 1.11E-02 | 9.11E-01 | 6.66E-01 | 1.01E+00 | 3.86E-02 | 9.23E-01 |
| **Gpr4** | 1.14E-02 | 1.48E+00 | 2.54E-01 | 7.69E-01 | 4.63E-01 | 1.14E+00 |
| **Mfn2** | 1.17E-02 | 9.18E-01 | 6.36E-02 | 1.05E+00 | 1.91E-01 | 9.63E-01 |
| **Gabrb2** | 1.18E-02 | 8.77E-01 | 9.67E-02 | 1.07E+00 | 2.16E-01 | 9.42E-01 |
| **Pik3cb** | 1.18E-02 | 9.51E-01 | 5.62E-01 | 1.02E+00 | 2.56E-01 | 9.68E-01 |
| **Nos1** | 1.20E-02 | 6.90E-01 | 2.76E-02 | 1.32E+00 | 2.37E-01 | 9.09E-01 |
| **Cacna1c** | 1.28E-02 | 7.86E-01 | 6.03E-01 | 1.05E+00 | 3.87E-02 | 8.23E-01 |
| **Gtf2b** | 1.31E-02 | 1.07E+00 | 7.09E-01 | 1.01E+00 | 2.67E-02 | 1.08E+00 |
| **Phf19** | 1.33E-02 | 1.94E+00 | 5.11E-01 | 7.86E-01 | 1.52E-01 | 1.52E+00 |
| **Chrna7** | 1.38E-02 | 8.43E-01 | 5.26E-02 | 1.19E+00 | 9.47E-01 | 1.01E+00 |
| **Cntnap2** | 1.39E-02 | 8.71E-01 | 1.32E-01 | 1.10E+00 | 5.32E-01 | 9.60E-01 |
| **Tnr** | 1.42E-02 | 8.40E-01 | 6.22E-02 | 1.12E+00 | 3.83E-01 | 9.43E-01 |
| **Mal** | 1.49E-02 | 1.26E+00 | 4.24E-01 | 8.96E-01 | 2.61E-01 | 1.13E+00 |
| **Scn2a1** | 1.51E-02 | 8.16E-01 | 4.95E-02 | 1.13E+00 | 2.24E-01 | 9.20E-01 |
| **Atrn** | 1.61E-02 | 8.68E-01 | 4.94E-02 | 1.08E+00 | 2.15E-01 | 9.34E-01 |
| **Cdkn1a** | 1.62E-02 | 1.72E+00 | 1.73E-01 | 6.87E-01 | 4.21E-01 | 1.18E+00 |
| **L1cam** | 1.63E-02 | 7.92E-01 | 5.69E-02 | 1.19E+00 | 6.21E-01 | 9.45E-01 |
| **Snap91** | 1.64E-02 | 8.91E-01 | 2.19E-01 | 1.06E+00 | 3.09E-01 | 9.45E-01 |
| **Sod2** | 1.67E-02 | 9.50E-01 | 9.58E-01 | 9.99E-01 | 7.23E-02 | 9.49E-01 |
| **Igf1r** | 1.71E-02 | 8.41E-01 | 1.99E-02 | 1.29E+00 | 4.30E-01 | 1.09E+00 |
| **Bcas1** | 1.75E-02 | 1.46E+00 | 1.22E-01 | 6.41E-01 | 7.82E-01 | 9.38E-01 |
| **Ptprn2** | 1.78E-02 | 8.60E-01 | 4.48E-01 | 1.05E+00 | 1.52E-01 | 9.01E-01 |
| **Atm** | 1.80E-02 | 8.26E-01 | 1.11E-01 | 1.15E+00 | 5.84E-01 | 9.49E-01 |
| **Ppp2r5e** | 1.87E-02 | 8.79E-01 | 2.86E-01 | 1.05E+00 | 1.50E-01 | 9.25E-01 |
| **Atp8a2** | 1.94E-02 | 8.05E-01 | 3.83E-02 | 1.18E+00 | 4.99E-01 | 9.53E-01 |
| **Ring1** | 1.95E-02 | 1.19E+00 | 1.38E-02 | 8.89E-01 | 3.66E-01 | 1.06E+00 |
| **Sorl1** | 2.01E-02 | 8.14E-01 | 1.45E-01 | 1.09E+00 | 1.20E-01 | 8.86E-01 |
| **Gfpt1** | 2.02E-02 | 9.22E-01 | 9.92E-03 | 1.13E+00 | 1.48E-01 | 1.04E+00 |
| **Ryr2** | 2.05E-02 | 7.81E-01 | 2.78E-01 | 1.13E+00 | 1.86E-01 | 8.78E-01 |
| **Mbp** | 2.20E-02 | 1.33E+00 | 1.49E-01 | 6.67E-01 | 6.06E-01 | 8.85E-01 |
| **Slc8a1** | 2.20E-02 | 7.98E-01 | 5.81E-03 | 1.30E+00 | 6.40E-01 | 1.04E+00 |
| **Dlat** | 2.23E-02 | 9.10E-01 | 6.58E-01 | 9.79E-01 | 3.73E-02 | 8.90E-01 |
| **Ddit3** | 2.24E-02 | 1.15E+00 | 3.16E-02 | 8.45E-01 | 5.98E-01 | 9.71E-01 |
| **Mapk1** | 2.26E-02 | 8.93E-01 | 3.87E-01 | 1.03E+00 | 1.55E-01 | 9.23E-01 |
| **Oxr1** | 2.31E-02 | 9.25E-01 | 3.26E-02 | 1.06E+00 | 6.14E-01 | 9.84E-01 |
| **Chl1** | 2.37E-02 | 8.19E-01 | 6.17E-02 | 1.15E+00 | 4.13E-01 | 9.44E-01 |
| **Htt** | 2.37E-02 | 8.74E-01 | 1.21E-01 | 1.11E+00 | 6.30E-01 | 9.68E-01 |
| **Synj1** | 2.42E-02 | 8.40E-01 | 8.38E-02 | 1.11E+00 | 3.74E-01 | 9.35E-01 |
| **Gria3** | 2.44E-02 | 8.16E-01 | 4.54E-02 | 1.10E+00 | 1.28E-01 | 8.96E-01 |
| **Trpm2** | 2.52E-02 | 8.57E-01 | 7.64E-02 | 1.16E+00 | 8.91E-01 | 9.92E-01 |
| **Cadm3** | 2.64E-02 | 8.74E-01 | 2.08E-01 | 1.12E+00 | 8.37E-01 | 9.80E-01 |
| **Cacnb4** | 2.66E-02 | 8.69E-01 | 6.22E-01 | 1.02E+00 | 3.17E-02 | 8.84E-01 |
| **Ube3a** | 2.67E-02 | 9.22E-01 | 5.67E-02 | 1.08E+00 | 8.40E-01 | 9.94E-01 |
| **Kcna1** | 2.67E-02 | 8.41E-01 | 2.52E-01 | 1.08E+00 | 2.72E-01 | 9.09E-01 |
| **Atp6v1a** | 2.75E-02 | 9.09E-01 | 3.00E-01 | 1.08E+00 | 8.39E-01 | 9.84E-01 |
| **Nfkbia** | 2.83E-02 | 1.36E+00 | 2.57E-01 | 8.71E-01 | 1.39E-01 | 1.19E+00 |
| **Grm1** | 2.93E-02 | 8.44E-01 | 3.18E-01 | 1.09E+00 | 4.10E-01 | 9.17E-01 |
| **Ube2k** | 3.16E-02 | 9.31E-01 | 9.09E-01 | 9.98E-01 | 1.82E-02 | 9.30E-01 |
| **Cacna1s** | 3.18E-02 | 1.26E+00 | 1.53E-01 | 9.20E-01 | 7.36E-02 | 1.16E+00 |
| **Il10** | 3.18E-02 | 4.06E-01 | 9.61E-01 | 1.01E+00 | 1.99E-02 | 4.10E-01 |
| **Atf6** | 3.19E-02 | 8.75E-01 | 7.52E-01 | 1.02E+00 | 1.13E-01 | 8.90E-01 |
| **Cspg4** | 3.20E-02 | 7.30E-01 | 1.13E-01 | 1.30E+00 | 7.16E-01 | 9.50E-01 |
| **Tie1** | 3.34E-02 | 1.36E+00 | 1.76E-01 | 1.14E+00 | 1.26E-02 | 1.56E+00 |
| **Bcl2l1** | 3.41E-02 | 1.16E+00 | 6.76E-02 | 8.93E-01 | 4.76E-01 | 1.03E+00 |
| **Ubqln1** | 3.43E-02 | 9.37E-01 | 7.11E-01 | 1.01E+00 | 1.02E-01 | 9.47E-01 |
| **Ugcg** | 3.46E-02 | 8.77E-01 | 4.66E-01 | 1.04E+00 | 1.14E-01 | 9.11E-01 |
| **Dagla** | 3.56E-02 | 8.65E-01 | 9.65E-01 | 9.98E-01 | 3.89E-02 | 8.63E-01 |
| **Pla2g4e** | 3.70E-02 | 1.90E+00 | 4.68E-02 | 5.61E-01 | 6.84E-01 | 1.07E+00 |
| **Cacna1a** | 3.85E-02 | 8.65E-01 | 2.96E-01 | 1.04E+00 | 1.03E-01 | 9.03E-01 |
| **Rapgef2** | 3.92E-02 | 8.33E-01 | 2.18E-01 | 1.06E+00 | 1.37E-01 | 8.79E-01 |
| **Cdc27** | 3.95E-02 | 9.05E-01 | 8.37E-04 | 1.14E+00 | 4.54E-01 | 1.03E+00 |
| **Gsk3b** | 4.05E-02 | 9.42E-01 | 3.39E-01 | 1.03E+00 | 4.88E-01 | 9.75E-01 |
| **Nmnat2** | 4.12E-02 | 9.10E-01 | 4.28E-01 | 9.68E-01 | 5.11E-02 | 8.81E-01 |
| **Epha6** | 4.17E-02 | 8.44E-01 | 4.73E-02 | 1.15E+00 | 6.63E-01 | 9.73E-01 |
| **Adcy9** | 4.31E-02 | 8.41E-01 | 9.40E-02 | 1.12E+00 | 5.21E-01 | 9.45E-01 |
| **Slc12a5** | 4.36E-02 | 8.22E-01 | 8.09E-01 | 1.01E+00 | 7.06E-02 | 8.31E-01 |
| **Ppm1l** | 4.73E-02 | 8.30E-01 | 1.56E-01 | 1.09E+00 | 2.06E-01 | 9.05E-01 |
| **Slc9a6** | 4.79E-02 | 9.42E-01 | 7.52E-02 | 1.06E+00 | 9.92E-01 | 1.00E+00 |
| **Gls** | 4.87E-02 | 9.54E-01 | 6.16E-01 | 1.02E+00 | 4.36E-01 | 9.71E-01 |
| **Inpp5f** | 4.98E-02 | 9.45E-01 | 8.40E-01 | 9.92E-01 | 1.27E-01 | 9.38E-01 |
| **Tradd** | 5.07E-02 | 1.38E+00 | 2.83E-01 | 8.57E-01 | 2.42E-01 | 1.18E+00 |
| **Sqstm1** | 5.09E-02 | 9.17E-01 | 4.31E-01 | 1.04E+00 | 4.11E-01 | 9.52E-01 |
| **Icam2** | 5.17E-02 | 1.29E+00 | 7.17E-01 | 1.09E+00 | 1.64E-01 | 1.41E+00 |
| **Hc** | 5.31E-02 | 1.78E+00 | 2.31E-01 | 7.04E-01 | 2.78E-01 | 1.25E+00 |
| **Cacna1d** | 5.36E-02 | 8.63E-01 | 1.22E-01 | 1.10E+00 | 2.48E-01 | 9.52E-01 |
| **Cadps** | 5.46E-02 | 9.51E-01 | 8.33E-01 | 1.00E+00 | 3.04E-02 | 9.55E-01 |
| **Inpp4a** | 5.74E-02 | 9.21E-01 | 8.77E-02 | 9.73E-01 | 1.94E-02 | 8.97E-01 |
| **Insr** | 5.81E-02 | 8.76E-01 | 2.57E-02 | 1.17E+00 | 7.32E-01 | 1.02E+00 |
| **Lrp1** | 5.85E-02 | 8.39E-01 | 2.23E-02 | 1.25E+00 | 5.92E-01 | 1.05E+00 |
| **Thy1** | 5.89E-02 | 9.39E-01 | 4.77E-01 | 1.04E+00 | 6.72E-01 | 9.76E-01 |
| **Apaf1** | 6.09E-02 | 8.35E-01 | 6.14E-02 | 1.20E+00 | 9.88E-01 | 1.00E+00 |
| **Syt1** | 6.22E-02 | 8.32E-01 | 6.01E-01 | 1.04E+00 | 2.52E-01 | 8.66E-01 |
| **Ap3m2** | 6.34E-02 | 8.89E-01 | 4.99E-01 | 1.04E+00 | 3.16E-01 | 9.27E-01 |
| **Mapk10** | 6.38E-02 | 9.00E-01 | 8.95E-01 | 1.00E+00 | 8.51E-02 | 9.05E-01 |
| **Gsr** | 6.42E-02 | 9.05E-01 | 5.87E-01 | 1.03E+00 | 1.58E-01 | 9.34E-01 |
| **Plcb1** | 6.43E-02 | 8.92E-01 | 2.86E-01 | 1.04E+00 | 1.95E-01 | 9.31E-01 |
| **Dgke** | 6.43E-02 | 9.04E-01 | 2.27E-01 | 1.08E+00 | 6.90E-01 | 9.72E-01 |
| **Ncl** | 6.44E-02 | 9.03E-01 | 9.80E-02 | 1.13E+00 | 7.77E-01 | 1.02E+00 |
| **Gnaq** | 6.44E-02 | 9.10E-01 | 5.85E-01 | 9.87E-01 | 3.98E-02 | 8.98E-01 |
| **Lsr** | 6.61E-02 | 1.31E+00 | 3.96E-02 | 1.44E+00 | 8.89E-04 | 1.88E+00 |
| **Unc13a** | 6.64E-02 | 8.95E-01 | 7.53E-01 | 9.89E-01 | 3.79E-02 | 8.85E-01 |
| **Gria1** | 6.64E-02 | 8.73E-01 | 7.90E-01 | 1.01E+00 | 1.10E-01 | 8.80E-01 |
| **Vcp** | 6.74E-02 | 9.19E-01 | 7.74E-01 | 1.01E+00 | 1.13E-01 | 9.28E-01 |
| **Slc17a6** | 7.14E-02 | 1.47E+00 | 6.74E-01 | 9.09E-01 | 2.38E-01 | 1.33E+00 |
| **Nts** | 7.31E-02 | 1.81E+00 | 8.36E-01 | 9.26E-01 | 1.90E-01 | 1.67E+00 |
| **Atp6v0c** | 7.32E-02 | 9.39E-01 | 3.61E-01 | 1.07E+00 | 9.20E-01 | 1.01E+00 |
| **Kif3a** | 7.40E-02 | 8.80E-01 | 6.90E-01 | 1.02E+00 | 2.22E-01 | 9.02E-01 |
| **Casp6** | 7.41E-02 | 1.46E+00 | 6.70E-01 | 9.60E-01 | 8.75E-02 | 1.40E+00 |
| **Ipcef1** | 7.43E-02 | 8.30E-01 | 2.51E-01 | 1.12E+00 | 3.92E-01 | 9.32E-01 |
| **Grm5** | 7.46E-02 | 8.61E-01 | 2.41E-01 | 1.07E+00 | 3.53E-01 | 9.23E-01 |
| **Atxn2** | 7.63E-02 | 9.02E-01 | 1.69E-01 | 1.04E+00 | 2.79E-01 | 9.41E-01 |
| **Rab3a** | 7.89E-02 | 8.91E-01 | 6.85E-01 | 9.70E-01 | 1.14E-01 | 8.64E-01 |
| **Gpr84** | 7.96E-02 | 5.90E-01 | 1.46E-01 | 1.41E+00 | 3.79E-01 | 8.32E-01 |
| **Atxn7** | 8.07E-02 | 1.15E+00 | 1.40E-01 | 9.13E-01 | 4.71E-01 | 1.05E+00 |
| **Tspo** | 8.10E-02 | 1.79E+00 | 1.19E-01 | 1.39E+00 | 2.86E-02 | 2.48E+00 |
| **Myh10** | 8.11E-02 | 8.92E-01 | 8.88E-03 | 1.09E+00 | 6.89E-01 | 9.75E-01 |
| **Sla** | 8.32E-02 | 6.37E-01 | 2.15E-01 | 1.38E+00 | 6.47E-01 | 8.77E-01 |
| **Cdk5r1** | 8.49E-02 | 9.28E-01 | 1.18E-01 | 9.43E-01 | 1.85E-02 | 8.75E-01 |
| **Prpf3** | 8.51E-02 | 1.08E+00 | 4.83E-02 | 9.00E-01 | 5.27E-01 | 9.74E-01 |
| **Atp6v1h** | 8.62E-02 | 9.44E-01 | 4.59E-01 | 1.04E+00 | 8.21E-01 | 9.86E-01 |
| **Gabrb3** | 8.69E-02 | 8.96E-01 | 5.48E-01 | 1.03E+00 | 1.71E-01 | 9.19E-01 |
| **Myd88** | 8.70E-02 | 1.27E+00 | 3.28E-01 | 8.66E-01 | 4.83E-01 | 1.10E+00 |
| **Cln8** | 8.72E-02 | 9.17E-01 | 6.71E-01 | 1.02E+00 | 2.60E-01 | 9.34E-01 |
| **Emp2** | 8.75E-02 | 1.27E+00 | 8.59E-01 | 1.01E+00 | 9.15E-02 | 1.29E+00 |
| **Nelfa** | 8.80E-02 | 9.31E-01 | 6.92E-01 | 1.02E+00 | 3.44E-01 | 9.50E-01 |
| **F2** | 8.89E-02 | 6.80E-01 | 5.73E-01 | 1.10E+00 | 1.19E-01 | 7.51E-01 |
| **Esam** | 8.97E-02 | 1.20E+00 | 4.52E-01 | 1.07E+00 | 2.70E-02 | 1.28E+00 |
| **Prl** | 9.27E-02 | 4.85E-01 | 8.63E-02 | 1.91E+00 | 7.87E-01 | 9.28E-01 |
| **Cnksr2** | 9.27E-02 | 9.02E-01 | 9.76E-01 | 9.99E-01 | 2.86E-02 | 9.01E-01 |
| **Prkce** | 9.46E-02 | 9.10E-01 | 5.32E-01 | 9.63E-01 | 7.61E-02 | 8.77E-01 |
| **Gaa** | 9.47E-02 | 8.59E-01 | 1.02E-01 | 1.12E+00 | 6.49E-01 | 9.61E-01 |
| **Gjb1** | 9.48E-02 | 8.83E-01 | 8.13E-01 | 1.03E+00 | 4.44E-01 | 9.06E-01 |
| **Myc** | 9.55E-02 | 8.85E-01 | 2.27E-02 | 1.17E+00 | 6.26E-01 | 1.04E+00 |
| **Magee1** | 9.57E-02 | 8.80E-01 | 7.81E-01 | 1.01E+00 | 2.01E-01 | 8.93E-01 |
| **App** | 9.73E-02 | 7.18E-01 | 2.63E-07 | 2.97E+00 | 3.85E-02 | 2.13E+00 |
| **Gpd1l** | 9.80E-02 | 9.04E-01 | 7.73E-01 | 9.89E-01 | 6.56E-02 | 8.95E-01 |
| **Rims1** | 9.81E-02 | 9.01E-01 | 5.20E-01 | 1.06E+00 | 5.62E-01 | 9.53E-01 |
| **Grin1** | 9.90E-02 | 8.75E-01 | 3.57E-01 | 1.07E+00 | 4.60E-01 | 9.35E-01 |
| **Taz** | 1.00E-01 | 1.17E+00 | 4.82E-01 | 9.48E-01 | 2.19E-01 | 1.10E+00 |
| **Dgkb** | 1.01E-01 | 9.32E-01 | 8.79E-02 | 1.08E+00 | 8.40E-01 | 1.01E+00 |
| **Hdac6** | 1.03E-01 | 1.20E+00 | 6.92E-03 | 8.39E-01 | 9.17E-01 | 1.01E+00 |
| **Amph** | 1.03E-01 | 9.12E-01 | 7.30E-01 | 1.03E+00 | 4.03E-01 | 9.38E-01 |
| **Gprasp1** | 1.03E-01 | 8.45E-01 | 4.51E-01 | 1.06E+00 | 3.16E-01 | 8.93E-01 |
| **Tbp** | 1.03E-01 | 1.12E+00 | 2.86E-02 | 8.91E-01 | 9.96E-01 | 1.00E+00 |
| **Nova1** | 1.05E-01 | 1.05E+00 | 9.44E-02 | 9.04E-01 | 3.42E-01 | 9.51E-01 |
| **Rtn4** | 1.06E-01 | 9.44E-01 | 5.15E-01 | 1.02E+00 | 2.38E-01 | 9.64E-01 |
| **Nell2** | 1.08E-01 | 8.63E-01 | 9.63E-01 | 9.96E-01 | 2.41E-01 | 8.60E-01 |
| **Cul1** | 1.10E-01 | 1.03E+00 | 1.39E-01 | 1.03E+00 | 1.92E-02 | 1.06E+00 |
| **Pla2g4d** | 1.11E-01 | 1.48E+00 | 7.04E-01 | 1.13E+00 | 3.95E-02 | 1.67E+00 |
| **Flt1** | 1.12E-01 | 8.83E-01 | 7.33E-03 | 1.35E+00 | 8.91E-02 | 1.20E+00 |
| **Scamp2** | 1.13E-01 | 1.18E+00 | 5.78E-01 | 1.03E+00 | 4.26E-02 | 1.22E+00 |
| **P2rx4** | 1.14E-01 | 9.23E-01 | 1.42E-01 | 1.11E+00 | 7.78E-01 | 1.02E+00 |
| **Ap2b1** | 1.16E-01 | 9.00E-01 | 8.27E-01 | 1.01E+00 | 1.58E-01 | 9.08E-01 |
| **Cxxc1** | 1.20E-01 | 1.14E+00 | 3.31E-02 | 9.07E-01 | 6.80E-01 | 1.03E+00 |
| **Sncaip** | 1.20E-01 | 1.24E+00 | 8.07E-01 | 1.03E+00 | 1.51E-01 | 1.27E+00 |
| **Rras** | 1.25E-01 | 1.42E+00 | 6.15E-01 | 1.03E+00 | 1.06E-01 | 1.47E+00 |
| **Sf3a2** | 1.27E-01 | 9.23E-01 | 7.07E-01 | 9.83E-01 | 3.49E-02 | 9.08E-01 |
| **Pmp22** | 1.27E-01 | 8.75E-01 | 5.15E-02 | 1.25E+00 | 3.95E-01 | 1.09E+00 |
| **Prkcg** | 1.29E-01 | 8.20E-01 | 5.60E-01 | 1.06E+00 | 3.58E-01 | 8.68E-01 |
| **Fgf14** | 1.29E-01 | 9.43E-01 | 2.96E-01 | 1.04E+00 | 2.64E-01 | 9.80E-01 |
| **Prkca** | 1.30E-01 | 8.71E-01 | 5.50E-01 | 1.04E+00 | 1.62E-01 | 9.10E-01 |
| **Cycs** | 1.30E-01 | 1.12E+00 | 2.22E-02 | 8.71E-01 | 6.87E-01 | 9.75E-01 |
| **Nptn** | 1.31E-01 | 9.30E-01 | 4.74E-01 | 1.04E+00 | 6.23E-01 | 9.67E-01 |
| **Ap4s1** | 1.31E-01 | 1.12E+00 | 2.89E-01 | 9.36E-01 | 5.64E-01 | 1.05E+00 |
| **Slc4a10** | 1.31E-01 | 9.03E-01 | 7.48E-03 | 1.25E+00 | 7.45E-02 | 1.12E+00 |
| **Sart1** | 1.32E-01 | 8.82E-01 | 8.47E-01 | 9.83E-01 | 9.49E-02 | 8.67E-01 |
| **Pla2g2f** | 1.33E-01 | 1.28E+00 | 2.50E-01 | 8.56E-01 | 4.83E-01 | 1.10E+00 |
| **Cntnap1** | 1.34E-01 | 8.95E-01 | 8.63E-01 | 9.93E-01 | 7.80E-02 | 8.88E-01 |
| **Cers2** | 1.34E-01 | 1.09E+00 | 9.78E-01 | 1.00E+00 | 2.77E-01 | 1.09E+00 |
| **Pqbp1** | 1.36E-01 | 9.65E-01 | 9.28E-01 | 1.00E+00 | 5.58E-01 | 9.69E-01 |
| **Gnb5** | 1.36E-01 | 9.31E-01 | 8.21E-01 | 1.01E+00 | 3.44E-01 | 9.43E-01 |
| **Syt4** | 1.38E-01 | 8.80E-01 | 3.48E-01 | 1.08E+00 | 6.48E-01 | 9.53E-01 |
| **Taf10** | 1.39E-01 | 1.17E+00 | 5.38E-01 | 9.65E-01 | 2.41E-01 | 1.13E+00 |
| **Mapk8** | 1.40E-01 | 9.55E-01 | 1.26E-02 | 1.12E+00 | 1.41E-01 | 1.07E+00 |
| **Nme5** | 1.41E-01 | 1.20E+00 | 3.12E-01 | 1.12E+00 | 6.30E-02 | 1.34E+00 |
| **Nsf** | 1.41E-01 | 8.51E-01 | 8.59E-01 | 1.01E+00 | 2.41E-01 | 8.63E-01 |
| **Ppp3cc** | 1.41E-01 | 1.10E+00 | 5.57E-01 | 9.54E-01 | 5.34E-01 | 1.05E+00 |
| **Nfe2l2** | 1.41E-01 | 1.16E+00 | 8.90E-01 | 1.01E+00 | 1.78E-01 | 1.17E+00 |
| **Bace1** | 1.42E-01 | 9.12E-01 | 4.55E-01 | 1.03E+00 | 3.43E-01 | 9.37E-01 |
| **Mog** | 1.42E-01 | 1.15E+00 | 7.78E-01 | 9.69E-01 | 2.72E-01 | 1.12E+00 |
| **Hif1a** | 1.43E-01 | 9.36E-01 | 1.16E-02 | 1.10E+00 | 5.21E-01 | 1.03E+00 |
| **Atp6v0e2** | 1.44E-01 | 9.54E-01 | 6.92E-01 | 9.82E-01 | 2.37E-01 | 9.37E-01 |
| **Fmr1** | 1.45E-01 | 9.36E-01 | 6.08E-01 | 1.02E+00 | 1.83E-01 | 9.56E-01 |
| **Syt7** | 1.47E-01 | 9.71E-01 | 1.35E-01 | 9.05E-01 | 6.87E-02 | 8.79E-01 |
| **Pak1** | 1.48E-01 | 9.32E-01 | 8.03E-01 | 1.01E+00 | 3.13E-01 | 9.44E-01 |
| **Ager** | 1.49E-01 | 1.38E+00 | 9.06E-01 | 9.78E-01 | 2.40E-01 | 1.35E+00 |
| **Hras** | 1.50E-01 | 1.12E+00 | 6.33E-04 | 8.93E-01 | 9.50E-01 | 1.00E+00 |
| **Rasgrp1** | 1.51E-01 | 9.07E-01 | 6.36E-01 | 1.02E+00 | 1.92E-01 | 9.28E-01 |
| **Islr2** | 1.52E-01 | 9.17E-01 | 7.02E-01 | 1.03E+00 | 4.80E-01 | 9.44E-01 |
| **Prkacb** | 1.52E-01 | 9.28E-01 | 3.43E-01 | 1.03E+00 | 3.94E-01 | 9.59E-01 |
| **Atp2b3** | 1.53E-01 | 8.37E-01 | 1.57E-02 | 1.29E+00 | 5.40E-01 | 1.08E+00 |
| **Fam126a** | 1.53E-01 | 1.14E+00 | 9.91E-01 | 9.99E-01 | 2.46E-01 | 1.13E+00 |
| **Il13ra1** | 1.54E-01 | 1.27E+00 | 1.20E-01 | 1.12E+00 | 6.07E-02 | 1.42E+00 |
| **Kel** | 1.55E-01 | 6.76E-01 | 5.35E-01 | 1.15E+00 | 4.07E-02 | 7.74E-01 |
| **Cd9** | 1.56E-01 | 1.41E+00 | 5.91E-02 | 1.27E+00 | 4.77E-02 | 1.80E+00 |
| **Sirt2** | 1.56E-01 | 1.10E+00 | 3.44E-01 | 9.46E-01 | 6.10E-01 | 1.04E+00 |
| **C3** | 1.57E-01 | 1.23E+00 | 4.73E-01 | 1.09E+00 | 1.04E-01 | 1.34E+00 |
| **Fgf12** | 1.57E-01 | 9.44E-01 | 4.44E-01 | 1.06E+00 | 9.72E-01 | 9.97E-01 |
| **Nostrin** | 1.58E-01 | 1.26E+00 | 1.10E-01 | 8.08E-01 | 8.92E-01 | 1.02E+00 |
| **Gstp1** | 1.58E-01 | 1.11E+00 | 7.37E-01 | 1.01E+00 | 1.57E-01 | 1.12E+00 |
| **Prnp** | 1.59E-01 | 7.38E-01 | 7.47E-07 | 3.55E+00 | 2.97E-02 | 2.62E+00 |
| **Arc** | 1.61E-01 | 1.53E+00 | 2.31E-02 | 6.69E-01 | 9.25E-01 | 1.02E+00 |
| **Mmp19** | 1.61E-01 | 1.43E+00 | 4.79E-01 | 1.12E+00 | 7.52E-02 | 1.60E+00 |
| **Flt4** | 1.62E-01 | 8.46E-01 | 2.58E-01 | 1.16E+00 | 8.96E-01 | 9.83E-01 |
| **Negr1** | 1.62E-01 | 8.80E-01 | 4.19E-01 | 1.06E+00 | 2.13E-01 | 9.31E-01 |
| **Mapt** | 1.64E-01 | 9.42E-01 | 7.65E-01 | 9.92E-01 | 6.92E-02 | 9.35E-01 |
| **Erbb3** | 1.64E-01 | 9.19E-01 | 9.71E-01 | 1.01E+00 | 6.13E-01 | 9.24E-01 |
| **Inhbb** | 1.64E-01 | 1.29E+00 | 3.34E-01 | 1.22E+00 | 2.69E-02 | 1.57E+00 |
| **Egf** | 1.65E-01 | 2.11E+00 | 7.19E-01 | 1.12E+00 | 1.58E-01 | 2.36E+00 |
| **Pvalb** | 1.68E-01 | 1.20E+00 | 1.33E-01 | 8.84E-01 | 6.73E-01 | 1.06E+00 |
| **Phf21a** | 1.69E-01 | 8.98E-01 | 3.20E-01 | 1.08E+00 | 5.58E-01 | 9.67E-01 |
| **Chd4** | 1.69E-01 | 9.23E-01 | 7.25E-02 | 1.08E+00 | 8.84E-01 | 9.92E-01 |
| **Pdgfrb** | 1.71E-01 | 1.28E+00 | 7.99E-01 | 1.04E+00 | 1.38E-01 | 1.33E+00 |
| **Clu** | 1.72E-01 | 1.27E+00 | 5.90E-01 | 1.05E+00 | 1.31E-01 | 1.33E+00 |
| **Mapk9** | 1.73E-01 | 9.59E-01 | 9.28E-01 | 1.00E+00 | 2.60E-01 | 9.61E-01 |
| **Arrb2** | 1.78E-01 | 9.08E-01 | 6.13E-01 | 9.72E-01 | 8.34E-02 | 8.82E-01 |
| **Ap3s1** | 1.80E-01 | 1.13E+00 | 3.86E-01 | 9.61E-01 | 3.85E-01 | 1.08E+00 |
| **Atp6v0e** | 1.82E-01 | 1.29E+00 | 2.72E-01 | 1.11E+00 | 9.36E-02 | 1.44E+00 |
| **Meaf6** | 1.83E-01 | 9.35E-01 | 2.83E-01 | 1.05E+00 | 6.91E-01 | 9.79E-01 |
| **Emcn** | 1.85E-01 | 1.21E+00 | 1.41E-01 | 1.24E+00 | 1.39E-02 | 1.50E+00 |
| **Acaa1a** | 1.85E-01 | 1.13E+00 | 8.45E-01 | 1.01E+00 | 1.48E-01 | 1.14E+00 |
| **Npc2** | 1.86E-01 | 1.09E+00 | 2.39E-01 | 1.11E+00 | 6.56E-02 | 1.21E+00 |
| **Pnkd** | 1.87E-01 | 9.18E-01 | 4.85E-03 | 9.39E-01 | 3.63E-02 | 8.62E-01 |
| **Lsm2** | 1.88E-01 | 1.08E+00 | 1.59E-01 | 9.12E-01 | 8.70E-01 | 9.89E-01 |
| **Arhgap44** | 1.88E-01 | 1.09E+00 | 1.45E-01 | 9.66E-01 | 3.97E-01 | 1.06E+00 |
| **Nqo1** | 1.89E-01 | 1.38E+00 | 1.36E-01 | 1.32E+00 | 4.57E-02 | 1.82E+00 |
| **Atp6v1d** | 1.90E-01 | 9.56E-01 | 6.92E-01 | 1.02E+00 | 7.03E-01 | 9.77E-01 |
| **Slc2a1** | 1.91E-01 | 1.23E+00 | 8.77E-01 | 1.02E+00 | 1.64E-01 | 1.26E+00 |
| **Jam3** | 1.93E-01 | 1.08E+00 | 9.59E-01 | 1.00E+00 | 2.83E-01 | 1.09E+00 |
| **Ccnd1** | 1.97E-01 | 8.73E-01 | 7.97E-01 | 1.02E+00 | 1.30E-01 | 8.91E-01 |
| **Fasl** | 1.97E-01 | 7.74E-01 | 2.29E-02 | 1.86E+00 | 8.76E-02 | 1.44E+00 |
| **Ctns** | 1.98E-01 | 1.09E+00 | 3.25E-01 | 9.76E-01 | 3.56E-01 | 1.06E+00 |
| **Tor1a** | 1.98E-01 | 1.05E+00 | 8.52E-02 | 9.36E-01 | 6.72E-01 | 9.84E-01 |
| **Stx2** | 1.99E-01 | 1.18E+00 | 2.87E-01 | 9.01E-01 | 5.92E-01 | 1.07E+00 |
| **Pde4d** | 2.01E-01 | 9.04E-01 | 5.90E-01 | 1.04E+00 | 1.81E-01 | 9.37E-01 |
| **Atcay** | 2.02E-01 | 9.39E-01 | 1.33E-01 | 9.44E-01 | 5.56E-02 | 8.86E-01 |
| **Cntf** | 2.04E-01 | 1.19E+00 | 9.13E-01 | 9.84E-01 | 2.04E-01 | 1.17E+00 |
| **Ep300** | 2.06E-01 | 9.00E-01 | 2.06E-01 | 1.09E+00 | 8.53E-01 | 9.84E-01 |
| **Park7** | 2.06E-01 | 1.09E+00 | 4.30E-01 | 9.80E-01 | 2.99E-01 | 1.07E+00 |
| **Hspb1** | 2.06E-01 | 1.33E+00 | 4.43E-01 | 1.16E+00 | 8.98E-02 | 1.54E+00 |
| **Ppp2r5c** | 2.07E-01 | 1.10E+00 | 2.18E-01 | 9.59E-01 | 4.65E-01 | 1.06E+00 |
| **Gsn** | 2.07E-01 | 1.23E+00 | 2.10E-02 | 1.28E+00 | 3.43E-02 | 1.58E+00 |
| **Chat** | 2.08E-01 | 1.13E+00 | 6.61E-01 | 8.30E-01 | 8.73E-01 | 9.38E-01 |
| **Gnai1** | 2.09E-01 | 9.41E-01 | 7.91E-01 | 1.01E+00 | 3.03E-01 | 9.51E-01 |
| **Cybb** | 2.09E-01 | 1.31E+00 | 9.04E-02 | 1.17E+00 | 8.94E-02 | 1.53E+00 |
| **Crh** | 2.09E-01 | 8.72E-01 | 4.61E-02 | 1.22E+00 | 6.09E-01 | 1.06E+00 |
| **Grm8** | 2.09E-01 | 9.04E-01 | 7.99E-02 | 1.21E+00 | 3.65E-01 | 1.09E+00 |
| **Rad23b** | 2.12E-01 | 9.52E-01 | 6.42E-01 | 1.02E+00 | 5.56E-01 | 9.71E-01 |
| **Col4a1** | 2.12E-01 | 1.26E+00 | 5.78E-01 | 9.33E-01 | 3.83E-01 | 1.17E+00 |
| **Cacnb2** | 2.13E-01 | 9.22E-01 | 7.53E-01 | 9.82E-01 | 2.65E-01 | 9.05E-01 |
| **Pfn1** | 2.14E-01 | 1.08E+00 | 6.59E-01 | 9.80E-01 | 3.95E-01 | 1.06E+00 |
| **Htra2** | 2.15E-01 | 1.17E+00 | 3.13E-01 | 8.91E-01 | 6.80E-01 | 1.04E+00 |
| **Cplx1** | 2.15E-01 | 9.57E-01 | 3.31E-01 | 9.54E-01 | 8.94E-02 | 9.13E-01 |
| **Plcb4** | 2.17E-01 | 1.24E+00 | 6.65E-01 | 9.07E-01 | 5.29E-01 | 1.13E+00 |
| **Gria2** | 2.18E-01 | 9.06E-01 | 8.73E-01 | 1.00E+00 | 2.46E-01 | 9.10E-01 |
| **Ccs** | 2.22E-01 | 1.14E+00 | 8.67E-01 | 1.01E+00 | 2.33E-01 | 1.15E+00 |
| **Angpt2** | 2.22E-01 | 1.41E+00 | 8.41E-02 | 1.76E+00 | 1.30E-02 | 2.48E+00 |
| **Efna5** | 2.22E-01 | 1.12E+00 | 3.79E-01 | 1.07E+00 | 1.10E-01 | 1.20E+00 |
| **Tbr1** | 2.23E-01 | 9.39E-01 | 4.05E-01 | 1.04E+00 | 6.84E-01 | 9.74E-01 |
| **Adora2a** | 2.24E-01 | 1.19E+00 | 5.72E-01 | 1.07E+00 | 1.59E-01 | 1.27E+00 |
| **Ptprr** | 2.24E-01 | 9.15E-01 | 3.30E-02 | 1.13E+00 | 6.90E-01 | 1.03E+00 |
| **Epha4** | 2.25E-01 | 8.95E-01 | 5.37E-01 | 1.04E+00 | 3.67E-01 | 9.31E-01 |
| **Akt2** | 2.26E-01 | 1.15E+00 | 3.51E-01 | 8.95E-01 | 7.96E-01 | 1.03E+00 |
| **Mta1** | 2.27E-01 | 1.02E+00 | 3.64E-02 | 9.41E-01 | 9.05E-02 | 9.61E-01 |
| **Bad** | 2.27E-01 | 1.17E+00 | 5.10E-01 | 9.41E-01 | 3.68E-01 | 1.10E+00 |
| **Tpm1** | 2.28E-01 | 1.08E+00 | 6.69E-01 | 1.01E+00 | 1.80E-01 | 1.09E+00 |
| **Slc18a3** | 2.29E-01 | 1.47E+00 | 2.52E-01 | 8.48E-01 | 4.30E-01 | 1.25E+00 |
| **Map2k2** | 2.31E-01 | 1.06E+00 | 1.02E-01 | 9.31E-01 | 6.39E-01 | 9.85E-01 |
| **Lsm7** | 2.34E-01 | 1.18E+00 | 2.81E-01 | 8.84E-01 | 7.59E-01 | 1.04E+00 |
| **Sptbn2** | 2.37E-01 | 1.12E+00 | 5.31E-01 | 9.43E-01 | 6.19E-01 | 1.06E+00 |
| **Pcsk2** | 2.39E-01 | 8.80E-01 | 4.63E-01 | 1.08E+00 | 5.98E-01 | 9.48E-01 |
| **Polr2l** | 2.39E-01 | 1.19E+00 | 1.64E-01 | 8.64E-01 | 8.31E-01 | 1.02E+00 |
| **Ap2a2** | 2.40E-01 | 9.34E-01 | 3.53E-01 | 1.04E+00 | 7.25E-01 | 9.75E-01 |
| **Man2b1** | 2.40E-01 | 9.23E-01 | 1.12E-04 | 1.45E+00 | 2.89E-03 | 1.34E+00 |
| **Lclat1** | 2.44E-01 | 9.38E-01 | 3.47E-01 | 1.06E+00 | 9.66E-01 | 9.98E-01 |
| **Grin3b** | 2.45E-01 | 1.58E+00 | 3.67E-02 | 6.28E-01 | 9.82E-01 | 9.93E-01 |
| **Sirt1** | 2.46E-01 | 9.39E-01 | 2.04E-01 | 1.05E+00 | 7.79E-01 | 9.85E-01 |
| **Camk2g** | 2.46E-01 | 9.71E-01 | 7.52E-01 | 1.01E+00 | 5.46E-01 | 9.80E-01 |
| **Polr2b** | 2.47E-01 | 1.05E+00 | 3.86E-02 | 1.08E+00 | 1.15E-02 | 1.14E+00 |
| **Fa2h** | 2.48E-01 | 1.12E+00 | 6.03E-01 | 9.39E-01 | 5.61E-01 | 1.06E+00 |
| **Ltbr** | 2.49E-01 | 1.18E+00 | 2.89E-01 | 1.14E+00 | 6.36E-02 | 1.34E+00 |
| **Aldh1l1** | 2.51E-01 | 1.44E+00 | 7.11E-01 | 9.51E-01 | 3.27E-01 | 1.37E+00 |
| **Chrm5** | 2.51E-01 | 8.58E-01 | 1.81E-01 | 1.22E+00 | 7.97E-01 | 1.05E+00 |
| **Slc18a2** | 2.53E-01 | 1.33E+00 | 9.48E-01 | 9.85E-01 | 1.29E-01 | 1.31E+00 |
| **3110043O21Rik** | 2.53E-01 | 1.07E+00 | 9.98E-02 | 9.25E-01 | 9.05E-01 | 9.92E-01 |
| **Polr2k** | 2.53E-01 | 1.25E+00 | 7.56E-01 | 9.50E-01 | 4.24E-01 | 1.19E+00 |
| **Bcas2** | 2.53E-01 | 1.10E+00 | 7.69E-01 | 9.84E-01 | 3.98E-01 | 1.08E+00 |
| **Serpinb6a** | 2.53E-01 | 1.11E+00 | 8.44E-01 | 1.02E+00 | 2.46E-01 | 1.13E+00 |
| **Comt** | 2.56E-01 | 1.06E+00 | 1.85E-01 | 1.03E+00 | 8.92E-02 | 1.10E+00 |
| **Ppargc1a** | 2.56E-01 | 9.37E-01 | 4.60E-01 | 1.02E+00 | 4.21E-01 | 9.56E-01 |
| **Dnm1l** | 2.56E-01 | 9.48E-01 | 9.95E-01 | 1.00E+00 | 3.28E-01 | 9.48E-01 |
| **Akt3** | 2.58E-01 | 9.52E-01 | 2.31E-01 | 9.61E-01 | 9.53E-02 | 9.15E-01 |
| **Galc** | 2.58E-01 | 1.10E+00 | 4.09E-01 | 1.06E+00 | 5.97E-02 | 1.16E+00 |
| **Ube2n** | 2.58E-01 | 9.63E-01 | 7.45E-01 | 9.87E-01 | 2.34E-01 | 9.50E-01 |
| **Dnm2** | 2.59E-01 | 1.08E+00 | 7.64E-01 | 9.80E-01 | 4.88E-01 | 1.06E+00 |
| **Dcx** | 2.61E-01 | 8.60E-01 | 5.46E-01 | 1.06E+00 | 3.79E-01 | 9.14E-01 |
| **Cck** | 2.65E-01 | 1.06E+00 | 3.44E-02 | 9.10E-01 | 5.05E-01 | 9.63E-01 |
| **Sirt7** | 2.65E-01 | 1.18E+00 | 3.40E-02 | 8.25E-01 | 8.47E-01 | 9.74E-01 |
| **Prkcq** | 2.67E-01 | 1.23E+00 | 3.17E-01 | 1.17E+00 | 1.15E-01 | 1.44E+00 |
| **Psen1** | 2.68E-01 | 9.34E-01 | 7.65E-01 | 1.02E+00 | 4.82E-01 | 9.49E-01 |
| **Nefl** | 2.68E-01 | 9.77E-01 | 1.90E-01 | 9.66E-01 | 3.76E-02 | 9.44E-01 |
| **Pla2g16** | 2.68E-01 | 1.16E+00 | 7.00E-01 | 1.05E+00 | 2.63E-01 | 1.22E+00 |
| **Mmp14** | 2.69E-01 | 1.15E+00 | 9.60E-01 | 9.97E-01 | 2.99E-01 | 1.15E+00 |
| **Hap1** | 2.70E-01 | 8.76E-01 | 4.97E-01 | 1.08E+00 | 5.62E-01 | 9.47E-01 |
| **Cd40** | 2.70E-01 | 1.24E+00 | 8.31E-03 | 2.74E+00 | 5.78E-03 | 3.39E+00 |
| **Polr2j** | 2.70E-01 | 1.09E+00 | 3.78E-01 | 9.44E-01 | 7.59E-01 | 1.03E+00 |
| **Usp30** | 2.72E-01 | 1.08E+00 | 4.52E-01 | 9.52E-01 | 5.94E-01 | 1.03E+00 |
| **Ryr3** | 2.72E-01 | 8.83E-01 | 8.34E-01 | 9.76E-01 | 1.55E-01 | 8.62E-01 |
| **Sucla2** | 2.74E-01 | 9.65E-01 | 3.62E-01 | 1.03E+00 | 8.11E-01 | 9.91E-01 |
| **Efnb3** | 2.74E-01 | 9.02E-01 | 9.77E-01 | 1.00E+00 | 2.68E-01 | 9.03E-01 |
| **Mgmt** | 2.76E-01 | 1.35E+00 | 9.11E-01 | 1.01E+00 | 2.51E-01 | 1.37E+00 |
| **Mmp9** | 2.79E-01 | 1.18E+00 | 4.67E-01 | 1.17E+00 | 9.54E-02 | 1.39E+00 |
| **Slc1a1** | 2.81E-01 | 8.98E-01 | 5.77E-01 | 1.03E+00 | 4.99E-01 | 9.26E-01 |
| **Ctnnb1** | 2.82E-01 | 1.05E+00 | 2.49E-01 | 1.03E+00 | 1.18E-01 | 1.08E+00 |
| **B4galt6** | 2.83E-01 | 9.42E-01 | 4.06E-01 | 1.03E+00 | 5.64E-01 | 9.68E-01 |
| **Lypla1** | 2.84E-01 | 1.11E+00 | 5.51E-01 | 1.03E+00 | 1.86E-01 | 1.14E+00 |
| **Ehmt1** | 2.87E-01 | 9.53E-01 | 9.74E-01 | 9.99E-01 | 3.75E-01 | 9.52E-01 |
| **Glrb** | 2.87E-01 | 9.63E-01 | 3.25E-01 | 1.03E+00 | 9.25E-01 | 9.96E-01 |
| **Sod1** | 2.88E-01 | 1.11E+00 | 9.69E-01 | 9.98E-01 | 3.13E-01 | 1.10E+00 |
| **Arhgef10** | 2.88E-01 | 1.12E+00 | 9.39E-01 | 1.01E+00 | 3.87E-01 | 1.13E+00 |
| **Cxcl11** | 2.91E-01 | 8.12E-01 | 1.14E-01 | 1.95E+00 | 2.51E-01 | 1.58E+00 |
| **H2-Ea-ps** | 2.91E-01 | 8.35E-01 | 2.22E-01 | 1.32E+00 | 6.49E-01 | 1.11E+00 |
| **C4a** | 2.94E-01 | 1.41E+00 | 6.11E-01 | 1.10E+00 | 2.31E-01 | 1.56E+00 |
| **Cp** | 2.94E-01 | 1.27E+00 | 2.77E-02 | 1.44E+00 | 4.36E-02 | 1.84E+00 |
| **Napsa** | 2.94E-01 | 1.15E+00 | 4.26E-01 | 1.17E+00 | 1.90E-01 | 1.35E+00 |
| **Bax** | 2.94E-01 | 1.13E+00 | 2.58E-01 | 9.46E-01 | 5.52E-01 | 1.07E+00 |
| **Npy** | 2.94E-01 | 1.59E+00 | 3.52E-02 | 8.93E-01 | 3.95E-01 | 1.42E+00 |
| **Ppp3ca** | 2.94E-01 | 9.09E-01 | 5.50E-01 | 9.73E-01 | 1.79E-01 | 8.84E-01 |
| **Adora1** | 2.98E-01 | 9.55E-01 | 2.41E-01 | 9.45E-01 | 1.45E-02 | 9.03E-01 |
| **Gnai2** | 2.99E-01 | 1.10E+00 | 7.31E-01 | 9.81E-01 | 4.39E-01 | 1.08E+00 |
| **Ran** | 2.99E-01 | 9.72E-01 | 2.10E-01 | 1.05E+00 | 6.00E-01 | 1.02E+00 |
| **Egfl7** | 2.99E-01 | 1.10E+00 | 7.63E-01 | 9.81E-01 | 3.79E-01 | 1.08E+00 |
| **Palm** | 3.00E-01 | 1.13E+00 | 2.19E-01 | 8.73E-01 | 9.19E-01 | 9.89E-01 |
| **Epo** | 3.00E-01 | 7.26E-01 | 6.81E-01 | 1.13E+00 | 6.40E-01 | 8.18E-01 |
| **Hnrnpm** | 3.01E-01 | 9.67E-01 | 6.21E-01 | 9.82E-01 | 2.50E-01 | 9.50E-01 |
| **Slu7** | 3.02E-01 | 9.63E-01 | 4.27E-01 | 9.76E-01 | 8.60E-02 | 9.39E-01 |
| **Prkcb** | 3.04E-01 | 9.61E-01 | 2.56E-01 | 9.52E-01 | 4.62E-02 | 9.15E-01 |
| **Map2k1** | 3.06E-01 | 9.32E-01 | 9.42E-01 | 9.96E-01 | 3.90E-01 | 9.29E-01 |
| **Pllp** | 3.08E-01 | 1.15E+00 | 2.22E-01 | 8.71E-01 | 9.83E-01 | 1.00E+00 |
| **Bnip3** | 3.08E-01 | 1.06E+00 | 5.19E-01 | 9.69E-01 | 6.71E-01 | 1.03E+00 |
| **Ncf1** | 3.11E-01 | 1.15E+00 | 9.43E-01 | 9.89E-01 | 4.74E-01 | 1.14E+00 |
| **Il6ra** | 3.11E-01 | 1.06E+00 | 1.97E-01 | 1.13E+00 | 8.44E-02 | 1.19E+00 |
| **Acvrl1** | 3.12E-01 | 1.15E+00 | 1.33E-01 | 1.28E+00 | 3.82E-03 | 1.46E+00 |
| **Prpf31** | 3.12E-01 | 9.50E-01 | 6.76E-02 | 1.10E+00 | 2.45E-01 | 1.04E+00 |
| **Homer1** | 3.15E-01 | 1.05E+00 | 4.08E-01 | 9.57E-01 | 9.02E-01 | 1.01E+00 |
| **Atp13a2** | 3.15E-01 | 1.11E+00 | 8.02E-02 | 9.18E-01 | 8.63E-01 | 1.02E+00 |
| **Xiap** | 3.15E-01 | 1.04E+00 | 8.58E-01 | 1.01E+00 | 4.65E-01 | 1.05E+00 |
| **Il6** | 3.16E-01 | 1.54E+00 | 1.49E-01 | 8.86E-01 | 4.33E-01 | 1.37E+00 |
| **Ppp2ca** | 3.17E-01 | 9.72E-01 | 8.61E-01 | 9.95E-01 | 3.89E-01 | 9.67E-01 |
| **Sri** | 3.17E-01 | 1.08E+00 | 4.19E-01 | 9.61E-01 | 6.40E-01 | 1.04E+00 |
| **Polr2h** | 3.23E-01 | 1.11E+00 | 5.67E-01 | 9.62E-01 | 5.29E-01 | 1.06E+00 |
| **Calm1** | 3.23E-01 | 1.06E+00 | 3.51E-01 | 9.50E-01 | 9.44E-01 | 1.00E+00 |
| **Tnfrsf1a** | 3.23E-01 | 1.17E+00 | 1.19E-01 | 1.26E+00 | 4.56E-02 | 1.48E+00 |
| **Cyp4x1** | 3.26E-01 | 8.86E-01 | 7.50E-01 | 1.04E+00 | 1.56E-01 | 9.18E-01 |
| **Plcl2** | 3.27E-01 | 1.06E+00 | 7.45E-01 | 9.82E-01 | 5.14E-01 | 1.04E+00 |
| **Bche** | 3.30E-01 | 8.61E-01 | 1.09E-01 | 1.25E+00 | 6.73E-01 | 1.08E+00 |
| **Grik2** | 3.34E-01 | 9.20E-01 | 5.50E-01 | 1.06E+00 | 5.49E-01 | 9.72E-01 |
| **Kcnj10** | 3.44E-01 | 1.52E+00 | 7.97E-01 | 9.61E-01 | 3.92E-01 | 1.46E+00 |
| **Myrf** | 3.45E-01 | 9.14E-01 | 8.31E-01 | 9.74E-01 | 4.12E-01 | 8.90E-01 |
| **Trp53** | 3.45E-01 | 1.09E+00 | 7.51E-01 | 9.73E-01 | 4.79E-01 | 1.06E+00 |
| **Chmp2b** | 3.46E-01 | 1.05E+00 | 4.98E-01 | 1.03E+00 | 1.78E-01 | 1.08E+00 |
| **Npas4** | 3.47E-01 | 1.45E+00 | 2.29E-01 | 6.25E-01 | 7.98E-01 | 9.04E-01 |
| **Htr5a** | 3.48E-01 | 8.95E-01 | 2.17E-01 | 1.19E+00 | 5.50E-01 | 1.07E+00 |
| **Pcna** | 3.48E-01 | 1.06E+00 | 6.54E-01 | 9.86E-01 | 4.77E-01 | 1.05E+00 |
| **Tnfrsf12a** | 3.48E-01 | 1.28E+00 | 7.12E-01 | 1.05E+00 | 2.70E-01 | 1.34E+00 |
| **Gdpd2** | 3.50E-01 | 1.20E+00 | 9.89E-01 | 1.00E+00 | 3.78E-01 | 1.21E+00 |
| **Cab39** | 3.50E-01 | 9.66E-01 | 6.24E-01 | 1.02E+00 | 8.01E-01 | 9.89E-01 |
| **Avp** | 3.52E-01 | 1.24E+00 | 2.79E-01 | 7.09E-01 | 5.65E-01 | 8.79E-01 |
| **Fos** | 3.53E-01 | 1.50E+00 | 1.73E-01 | 7.10E-01 | 8.55E-01 | 1.06E+00 |
| **Gng2** | 3.53E-01 | 9.54E-01 | 4.40E-01 | 9.64E-01 | 9.07E-02 | 9.19E-01 |
| **Cx3cl1** | 3.54E-01 | 9.28E-01 | 5.83E-01 | 9.62E-01 | 2.41E-01 | 8.92E-01 |
| **Pik3r1** | 3.57E-01 | 1.08E+00 | 9.24E-01 | 9.92E-01 | 4.12E-01 | 1.07E+00 |
| **Tcirg1** | 3.57E-01 | 1.25E+00 | 4.29E-01 | 1.15E+00 | 1.18E-01 | 1.44E+00 |
| **Gabra4** | 3.60E-01 | 1.05E+00 | 4.59E-01 | 1.02E+00 | 2.55E-01 | 1.07E+00 |
| **Myct1** | 3.60E-01 | 1.24E+00 | 3.02E-01 | 1.29E+00 | 1.15E-01 | 1.60E+00 |
| **Adrb2** | 3.61E-01 | 1.10E+00 | 5.62E-01 | 1.07E+00 | 2.41E-01 | 1.18E+00 |
| **Cdk5** | 3.61E-01 | 1.08E+00 | 1.84E-01 | 9.31E-01 | 9.67E-01 | 1.00E+00 |
| **Psmb8** | 3.62E-01 | 1.20E+00 | 5.01E-02 | 1.44E+00 | 4.15E-02 | 1.73E+00 |
| **Rhoa** | 3.63E-01 | 1.05E+00 | 3.02E-01 | 1.04E+00 | 1.63E-01 | 1.09E+00 |
| **Plcb3** | 3.64E-01 | 1.18E+00 | 8.94E-01 | 9.89E-01 | 4.06E-01 | 1.17E+00 |
| **Fxn** | 3.65E-01 | 1.08E+00 | 5.77E-01 | 1.04E+00 | 2.65E-01 | 1.13E+00 |
| **Usp21** | 3.66E-01 | 1.06E+00 | 1.06E-01 | 9.03E-01 | 3.91E-01 | 9.55E-01 |
| **Dlx1** | 3.68E-01 | 1.19E+00 | 4.30E-01 | 8.48E-01 | 9.76E-01 | 1.01E+00 |
| **Icam1** | 3.70E-01 | 1.94E+00 | 1.53E-02 | 1.82E+00 | 2.02E-01 | 3.53E+00 |
| **Twistnb** | 3.71E-01 | 1.06E+00 | 2.30E-01 | 9.67E-01 | 7.32E-01 | 1.02E+00 |
| **Cers4** | 3.72E-01 | 1.12E+00 | 9.34E-01 | 1.01E+00 | 3.02E-01 | 1.13E+00 |
| **Smyd1** | 3.72E-01 | 1.18E+00 | 8.21E-01 | 9.51E-01 | 5.97E-01 | 1.12E+00 |
| **Wfs1** | 3.75E-01 | 9.36E-01 | 6.64E-01 | 1.03E+00 | 7.14E-01 | 9.68E-01 |
| **Casp8** | 3.79E-01 | 1.20E+00 | 4.80E-01 | 1.12E+00 | 1.32E-01 | 1.35E+00 |
| **Prkaa2** | 3.79E-01 | 9.61E-01 | 7.19E-02 | 1.05E+00 | 8.10E-01 | 1.01E+00 |
| **S100b** | 3.82E-01 | 1.17E+00 | 4.40E-01 | 8.78E-01 | 9.04E-01 | 1.03E+00 |
| **Gnptg** | 3.84E-01 | 1.07E+00 | 8.02E-01 | 9.82E-01 | 5.44E-01 | 1.05E+00 |
| **Ache** | 3.87E-01 | 1.09E+00 | 4.92E-01 | 9.57E-01 | 6.95E-01 | 1.04E+00 |
| **Cxcl16** | 3.87E-01 | 1.27E+00 | 5.15E-02 | 1.49E+00 | 6.39E-02 | 1.89E+00 |
| **Dll4** | 3.90E-01 | 1.17E+00 | 3.41E-01 | 1.16E+00 | 1.79E-01 | 1.36E+00 |
| **Plekho2** | 3.90E-01 | 8.83E-01 | 5.40E-01 | 1.12E+00 | 9.56E-01 | 9.91E-01 |
| **Smn1** | 3.91E-01 | 1.07E+00 | 8.99E-01 | 9.91E-01 | 5.14E-01 | 1.06E+00 |
| **Aqp4** | 3.91E-01 | 1.18E+00 | 4.28E-01 | 1.12E+00 | 2.11E-01 | 1.32E+00 |
| **Entpd2** | 3.91E-01 | 1.16E+00 | 8.58E-01 | 9.82E-01 | 4.05E-01 | 1.14E+00 |
| **Calb1** | 3.92E-01 | 8.44E-01 | 4.21E-01 | 9.11E-01 | 1.46E-01 | 7.69E-01 |
| **Aif1** | 3.92E-01 | 1.20E+00 | 5.09E-01 | 1.15E+00 | 2.19E-01 | 1.38E+00 |
| **Atf4** | 3.93E-01 | 9.53E-01 | 7.63E-01 | 1.02E+00 | 7.52E-01 | 9.73E-01 |
| **Atp6v0d1** | 3.93E-01 | 9.86E-01 | 3.04E-01 | 9.61E-01 | 1.83E-01 | 9.48E-01 |
| **Atg5** | 3.97E-01 | 1.05E+00 | 7.03E-01 | 9.81E-01 | 6.56E-01 | 1.03E+00 |
| **Gfap** | 3.98E-01 | 2.07E+00 | 9.44E-02 | 1.48E+00 | 2.80E-01 | 3.06E+00 |
| **Mmp12** | 4.03E-01 | 7.46E-01 | 8.48E-01 | 9.46E-01 | 1.40E-01 | 7.06E-01 |
| **Olig2** | 4.05E-01 | 9.62E-01 | 5.37E-01 | 9.42E-01 | 3.19E-01 | 9.07E-01 |
| **Jun** | 4.06E-01 | 9.59E-01 | 7.58E-01 | 1.02E+00 | 7.89E-01 | 9.79E-01 |
| **Eif2s1** | 4.09E-01 | 1.08E+00 | 8.96E-01 | 9.90E-01 | 5.13E-01 | 1.07E+00 |
| **Cers6** | 4.09E-01 | 1.03E+00 | 6.03E-01 | 9.75E-01 | 9.93E-01 | 1.00E+00 |
| **Neo1** | 4.10E-01 | 9.68E-01 | 5.97E-02 | 1.12E+00 | 1.54E-01 | 1.08E+00 |
| **Camk4** | 4.12E-01 | 9.41E-01 | 9.75E-01 | 1.00E+00 | 3.89E-01 | 9.43E-01 |
| **Fas** | 4.13E-01 | 1.08E+00 | 1.04E-01 | 1.28E+00 | 9.11E-03 | 1.38E+00 |
| **Stat1** | 4.13E-01 | 1.10E+00 | 3.44E-03 | 1.26E+00 | 3.02E-02 | 1.38E+00 |
| **Dlg3** | 4.14E-01 | 9.24E-01 | 4.04E-01 | 9.50E-01 | 1.91E-01 | 8.77E-01 |
| **Mapkapk2** | 4.15E-01 | 1.08E+00 | 8.84E-01 | 9.90E-01 | 4.23E-01 | 1.07E+00 |
| **C1qc** | 4.15E-01 | 1.21E+00 | 1.15E-01 | 1.30E+00 | 9.84E-02 | 1.57E+00 |
| **C1qb** | 4.16E-01 | 1.17E+00 | 1.61E-01 | 1.25E+00 | 8.32E-02 | 1.46E+00 |
| **Grin2c** | 4.17E-01 | 1.13E+00 | 5.49E-01 | 9.12E-01 | 8.73E-01 | 1.03E+00 |
| **Taf4** | 4.17E-01 | 1.09E+00 | 4.39E-01 | 9.14E-01 | 9.46E-01 | 9.93E-01 |
| **Trem1** | 4.17E-01 | 1.57E+00 | 8.21E-01 | 1.09E+00 | 3.67E-01 | 1.71E+00 |
| **Chrnb2** | 4.20E-01 | 9.11E-01 | 5.59E-01 | 9.64E-01 | 3.00E-01 | 8.78E-01 |
| **Ccr5** | 4.20E-01 | 1.17E+00 | 5.27E-02 | 1.32E+00 | 6.92E-02 | 1.54E+00 |
| **Tlr2** | 4.22E-01 | 8.34E-01 | 4.85E-02 | 1.54E+00 | 2.31E-01 | 1.28E+00 |
| **Cacna1f** | 4.25E-01 | 7.65E-01 | 5.04E-01 | 7.69E-01 | 2.23E-01 | 5.89E-01 |
| **Htr1a** | 4.27E-01 | 9.53E-01 | 2.86E-01 | 9.54E-01 | 1.67E-01 | 9.09E-01 |
| **Pten** | 4.28E-01 | 1.03E+00 | 6.08E-01 | 9.84E-01 | 7.02E-01 | 1.01E+00 |
| **Nmb** | 4.31E-01 | 1.20E+00 | 1.80E-01 | 8.43E-01 | 9.70E-01 | 1.01E+00 |
| **Pink1** | 4.32E-01 | 1.05E+00 | 2.36E-01 | 9.33E-01 | 7.75E-01 | 9.79E-01 |
| **Gnao1** | 4.33E-01 | 9.79E-01 | 1.29E-01 | 9.71E-01 | 5.37E-02 | 9.50E-01 |
| **Hdac7** | 4.33E-01 | 1.10E+00 | 5.44E-01 | 9.48E-01 | 7.33E-01 | 1.04E+00 |
| **Entpd4** | 4.34E-01 | 9.00E-01 | 1.54E-01 | 1.16E+00 | 7.62E-01 | 1.04E+00 |
| **Gata2** | 4.35E-01 | 9.19E-01 | 1.81E-01 | 1.25E+00 | 4.72E-01 | 1.15E+00 |
| **Fyn** | 4.35E-01 | 1.02E+00 | 8.31E-02 | 9.46E-01 | 2.45E-01 | 9.67E-01 |
| **Ppp3cb** | 4.39E-01 | 9.79E-01 | 5.05E-01 | 9.73E-01 | 2.56E-01 | 9.52E-01 |
| **Gnai3** | 4.39E-01 | 1.04E+00 | 1.64E-01 | 1.09E+00 | 1.36E-01 | 1.14E+00 |
| **Pik3ca** | 4.42E-01 | 9.64E-01 | 2.12E-01 | 1.06E+00 | 6.81E-01 | 1.02E+00 |
| **AI464131** | 4.43E-01 | 1.07E+00 | 6.05E-01 | 9.43E-01 | 9.65E-01 | 1.00E+00 |
| **Ccr2** | 4.44E-01 | 8.37E-01 | 4.27E-01 | 1.24E+00 | 8.90E-01 | 1.04E+00 |
| **Akt1s1** | 4.45E-01 | 1.05E+00 | 2.07E-01 | 9.41E-01 | 8.28E-01 | 9.88E-01 |
| **Marco** | 4.48E-01 | 8.61E-01 | 9.28E-02 | 1.43E+00 | 1.91E-01 | 1.23E+00 |
| **Fus** | 4.50E-01 | 9.71E-01 | 3.07E-01 | 1.02E+00 | 8.44E-01 | 9.92E-01 |
| **Idh1** | 4.51E-01 | 1.11E+00 | 3.28E-02 | 1.12E+00 | 1.48E-01 | 1.24E+00 |
| **Xbp1** | 4.53E-01 | 1.05E+00 | 1.90E-01 | 9.10E-01 | 4.21E-01 | 9.59E-01 |
| **Mmp24** | 4.55E-01 | 1.06E+00 | 1.69E-01 | 8.72E-01 | 2.69E-01 | 9.26E-01 |
| **Txnl1** | 4.58E-01 | 1.06E+00 | 6.80E-01 | 1.04E+00 | 3.76E-01 | 1.10E+00 |
| **Ins2** | 4.58E-01 | 1.41E+00 | 2.54E-02 | 1.94E+00 | 1.20E-01 | 2.72E+00 |
| **Becn1** | 4.60E-01 | 1.04E+00 | 1.73E-01 | 9.65E-01 | 9.98E-01 | 1.00E+00 |
| **Gnptab** | 4.60E-01 | 9.62E-01 | 6.11E-01 | 1.03E+00 | 7.60E-01 | 9.87E-01 |
| **Gpr37** | 4.61E-01 | 9.57E-01 | 8.22E-01 | 9.83E-01 | 4.14E-01 | 9.41E-01 |
| **Apoe** | 4.61E-01 | 1.09E+00 | 9.23E-01 | 9.91E-01 | 5.78E-01 | 1.08E+00 |
| **Fn1** | 4.64E-01 | 8.92E-01 | 1.36E-02 | 1.61E+00 | 5.87E-02 | 1.44E+00 |
| **Egr1** | 4.64E-01 | 1.20E+00 | 3.34E-01 | 8.72E-01 | 8.20E-01 | 1.05E+00 |
| **Stat3** | 4.66E-01 | 1.09E+00 | 2.15E-01 | 1.11E+00 | 1.89E-01 | 1.21E+00 |
| **Pla2g4a** | 4.66E-01 | 1.15E+00 | 9.68E-01 | 1.01E+00 | 3.95E-01 | 1.16E+00 |
| **Rit2** | 4.68E-01 | 1.04E+00 | 8.75E-01 | 9.91E-01 | 6.64E-01 | 1.03E+00 |
| **Naglu** | 4.68E-01 | 1.07E+00 | 2.77E-03 | 1.31E+00 | 1.36E-02 | 1.40E+00 |
| **Egr2** | 4.70E-01 | 1.34E+00 | 4.08E-01 | 9.15E-01 | 5.97E-01 | 1.23E+00 |
| **Pecam1** | 4.70E-01 | 1.08E+00 | 1.36E-02 | 1.31E+00 | 1.02E-02 | 1.41E+00 |
| **Dot1l** | 4.72E-01 | 1.11E+00 | 1.71E-01 | 8.26E-01 | 5.76E-01 | 9.19E-01 |
| **Shh** | 4.75E-01 | 9.28E-01 | 4.99E-01 | 9.57E-01 | 2.55E-01 | 8.88E-01 |
| **Rac1** | 4.79E-01 | 1.02E+00 | 4.50E-01 | 1.03E+00 | 1.63E-01 | 1.04E+00 |
| **Ina** | 4.80E-01 | 9.56E-01 | 8.81E-01 | 1.01E+00 | 5.97E-01 | 9.64E-01 |
| **Gabra1** | 4.81E-01 | 9.69E-01 | 1.01E-01 | 1.07E+00 | 4.10E-01 | 1.04E+00 |
| **Pla2g4b** | 4.82E-01 | 1.14E+00 | 8.29E-01 | 9.64E-01 | 4.26E-01 | 1.10E+00 |
| **Park2** | 4.85E-01 | 1.17E+00 | 1.12E-01 | 8.70E-01 | 9.36E-01 | 1.02E+00 |
| **Col4a2** | 4.87E-01 | 1.13E+00 | 2.97E-01 | 8.37E-01 | 7.98E-01 | 9.47E-01 |
| **Nos3** | 4.87E-01 | 1.12E+00 | 5.64E-01 | 1.10E+00 | 2.85E-01 | 1.23E+00 |
| **Drd4** | 4.89E-01 | 1.25E+00 | 9.33E-01 | 1.02E+00 | 4.43E-01 | 1.27E+00 |
| **Ugt8a** | 4.89E-01 | 1.04E+00 | 8.43E-01 | 1.03E+00 | 6.02E-01 | 1.07E+00 |
| **Nlrp3** | 4.90E-01 | 8.84E-01 | 3.86E-01 | 8.76E-01 | 3.75E-02 | 7.74E-01 |
| **Gtf2ird1** | 4.90E-01 | 1.06E+00 | 3.98E-01 | 1.06E+00 | 2.84E-01 | 1.13E+00 |
| **Fcrls** | 4.92E-01 | 1.17E+00 | 1.36E-01 | 1.31E+00 | 7.13E-02 | 1.54E+00 |
| **Rela** | 4.94E-01 | 1.07E+00 | 2.20E-01 | 1.11E+00 | 1.99E-01 | 1.18E+00 |
| **Notch1** | 4.95E-01 | 9.40E-01 | 1.45E-01 | 1.18E+00 | 1.40E-01 | 1.11E+00 |
| **Casp1** | 4.96E-01 | 8.63E-01 | 1.57E-01 | 1.31E+00 | 6.90E-01 | 1.13E+00 |
| **P2ry12** | 4.99E-01 | 8.82E-01 | 2.79E-01 | 1.15E+00 | 9.26E-01 | 1.02E+00 |
| **Adcyap1** | 4.99E-01 | 1.08E+00 | 6.32E-01 | 1.05E+00 | 1.72E-01 | 1.14E+00 |
| **Dnaja2** | 5.00E-01 | 9.62E-01 | 6.95E-01 | 1.03E+00 | 9.12E-01 | 9.91E-01 |
| **Uchl1** | 5.00E-01 | 9.20E-01 | 8.46E-01 | 9.78E-01 | 2.69E-01 | 9.00E-01 |
| **Katna1** | 5.01E-01 | 1.06E+00 | 9.76E-01 | 1.00E+00 | 5.97E-01 | 1.06E+00 |
| **Lamp1** | 5.02E-01 | 1.05E+00 | 6.84E-01 | 1.02E+00 | 4.06E-01 | 1.07E+00 |
| **Trim28** | 5.04E-01 | 9.74E-01 | 9.16E-01 | 9.95E-01 | 5.38E-01 | 9.69E-01 |
| **Stab1** | 5.05E-01 | 1.11E+00 | 3.64E-01 | 1.17E+00 | 1.54E-01 | 1.30E+00 |
| **Hmgb1** | 5.06E-01 | 1.03E+00 | 9.16E-01 | 1.00E+00 | 5.27E-01 | 1.03E+00 |
| **Car2** | 5.07E-01 | 9.67E-01 | 8.48E-01 | 1.02E+00 | 9.03E-01 | 9.86E-01 |
| **Sh3tc2** | 5.08E-01 | 9.08E-01 | 9.07E-01 | 9.83E-01 | 3.15E-01 | 8.93E-01 |
| **Sf3b2** | 5.10E-01 | 9.86E-01 | 1.58E-01 | 9.48E-01 | 8.40E-02 | 9.35E-01 |
| **Gusb** | 5.10E-01 | 1.13E+00 | 1.76E-01 | 1.26E+00 | 1.40E-01 | 1.42E+00 |
| **Cd4** | 5.11E-01 | 1.11E+00 | 9.55E-01 | 9.87E-01 | 7.04E-01 | 1.10E+00 |
| **Prf1** | 5.11E-01 | 8.37E-01 | 5.48E-01 | 1.15E+00 | 8.72E-01 | 9.59E-01 |
| **Actn1** | 5.15E-01 | 9.51E-01 | 4.15E-01 | 1.05E+00 | 9.93E-01 | 1.00E+00 |
| **Rdx** | 5.16E-01 | 1.04E+00 | 8.31E-02 | 1.10E+00 | 9.87E-02 | 1.14E+00 |
| **Cul3** | 5.16E-01 | 9.74E-01 | 5.42E-01 | 1.02E+00 | 8.88E-01 | 9.94E-01 |
| **Xk** | 5.16E-01 | 9.59E-01 | 4.85E-01 | 1.04E+00 | 9.89E-01 | 1.00E+00 |
| **Pdpk1** | 5.19E-01 | 9.82E-01 | 9.04E-02 | 9.61E-01 | 9.70E-02 | 9.44E-01 |
| **Plxnb3** | 5.24E-01 | 9.37E-01 | 4.79E-01 | 9.05E-01 | 2.10E-01 | 8.48E-01 |
| **Ptgs2** | 5.26E-01 | 9.56E-01 | 3.39E-01 | 1.11E+00 | 6.54E-01 | 1.06E+00 |
| **Gad1** | 5.28E-01 | 9.47E-01 | 1.37E-01 | 8.82E-01 | 2.80E-02 | 8.35E-01 |
| **Cx3cr1** | 5.34E-01 | 8.83E-01 | 1.36E-01 | 1.33E+00 | 3.25E-01 | 1.17E+00 |
| **Slc6a3** | 5.36E-01 | 7.11E-01 | 3.81E-01 | 1.60E+00 | 7.41E-01 | 1.14E+00 |
| **Calm4** | 5.38E-01 | 1.21E+00 | 5.95E-01 | 8.48E-01 | 9.11E-01 | 1.03E+00 |
| **Drd1** | 5.38E-01 | 1.46E+00 | 3.44E-01 | 1.30E+00 | 3.58E-01 | 1.89E+00 |
| **Tlr4** | 5.40E-01 | 1.15E+00 | 1.22E-01 | 1.27E+00 | 1.77E-01 | 1.46E+00 |
| **Tardbp** | 5.41E-01 | 1.03E+00 | 6.85E-01 | 9.76E-01 | 9.37E-01 | 1.00E+00 |
| **Tfam** | 5.42E-01 | 1.04E+00 | 7.33E-01 | 1.02E+00 | 4.63E-01 | 1.06E+00 |
| **Sox9** | 5.45E-01 | 1.09E+00 | 4.54E-01 | 1.08E+00 | 3.35E-01 | 1.17E+00 |
| **Ddc** | 5.46E-01 | 1.11E+00 | 9.28E-02 | 1.31E+00 | 4.16E-02 | 1.45E+00 |
| **Cdc40** | 5.50E-01 | 9.74E-01 | 6.65E-01 | 9.88E-01 | 4.52E-01 | 9.63E-01 |
| **Frmpd4** | 5.51E-01 | 9.61E-01 | 8.28E-01 | 1.01E+00 | 6.53E-01 | 9.73E-01 |
| **Itgal** | 5.51E-01 | 9.19E-01 | 9.72E-01 | 9.95E-01 | 6.42E-01 | 9.14E-01 |
| **Amigo1** | 5.56E-01 | 9.61E-01 | 4.44E-01 | 9.59E-01 | 2.67E-01 | 9.21E-01 |
| **Snrpa** | 5.56E-01 | 9.82E-01 | 2.43E-01 | 9.39E-01 | 1.59E-01 | 9.22E-01 |
| **Osmr** | 5.56E-01 | 1.09E+00 | 5.06E-02 | 1.35E+00 | 1.83E-02 | 1.47E+00 |
| **Epha7** | 5.56E-01 | 1.04E+00 | 7.91E-02 | 8.88E-01 | 2.49E-01 | 9.21E-01 |
| **Trem2** | 5.59E-01 | 8.80E-01 | 3.26E-02 | 1.72E+00 | 6.74E-02 | 1.51E+00 |
| **Mthfr** | 5.60E-01 | 8.68E-01 | 4.35E-01 | 1.20E+00 | 8.25E-01 | 1.04E+00 |
| **Mnat1** | 5.64E-01 | 1.04E+00 | 8.27E-01 | 1.02E+00 | 5.27E-01 | 1.05E+00 |
| **Dlgap1** | 5.64E-01 | 9.67E-01 | 3.45E-01 | 9.43E-01 | 1.46E-01 | 9.12E-01 |
| **Cd14** | 5.68E-01 | 9.17E-01 | 1.56E-02 | 1.75E+00 | 1.67E-02 | 1.61E+00 |
| **Nos2** | 5.68E-01 | 1.12E+00 | 1.80E-01 | 1.23E+00 | 1.31E-01 | 1.37E+00 |
| **Tmem119** | 5.69E-01 | 1.05E+00 | 1.34E-01 | 1.11E+00 | 1.68E-01 | 1.17E+00 |
| **Slc11a1** | 5.69E-01 | 1.13E+00 | 2.51E-01 | 1.31E+00 | 1.13E-01 | 1.48E+00 |
| **Zfp24** | 5.72E-01 | 1.04E+00 | 3.05E-02 | 1.12E+00 | 9.87E-02 | 1.17E+00 |
| **Cds1** | 5.73E-01 | 9.66E-01 | 2.72E-01 | 1.07E+00 | 6.06E-01 | 1.04E+00 |
| **Msn** | 5.75E-01 | 1.13E+00 | 1.61E-02 | 1.32E+00 | 1.42E-01 | 1.49E+00 |
| **Acin1** | 5.75E-01 | 9.64E-01 | 2.55E-01 | 9.44E-01 | 2.09E-01 | 9.10E-01 |
| **Itga7** | 5.75E-01 | 9.42E-01 | 2.04E-01 | 8.80E-01 | 3.18E-02 | 8.29E-01 |
| **Ccl5** | 5.77E-01 | 1.16E+00 | 2.38E-01 | 1.46E+00 | 2.08E-01 | 1.70E+00 |
| **Camk2b** | 5.77E-01 | 9.55E-01 | 1.33E-01 | 9.25E-01 | 1.67E-01 | 8.84E-01 |
| **Gabrg2** | 5.77E-01 | 1.03E+00 | 1.60E-01 | 1.04E+00 | 2.06E-01 | 1.06E+00 |
| **Dlg4** | 5.78E-01 | 1.04E+00 | 1.51E-01 | 8.87E-01 | 2.47E-01 | 9.19E-01 |
| **Abl1** | 5.79E-01 | 1.03E+00 | 6.41E-01 | 1.03E+00 | 3.76E-01 | 1.07E+00 |
| **Camk2d** | 5.79E-01 | 9.05E-01 | 5.69E-01 | 1.12E+00 | 9.48E-01 | 1.01E+00 |
| **Abat** | 5.80E-01 | 1.03E+00 | 4.83E-01 | 9.49E-01 | 7.83E-01 | 9.79E-01 |
| **Gal3st1** | 5.86E-01 | 1.04E+00 | 2.20E-01 | 8.71E-01 | 3.32E-01 | 9.09E-01 |
| **Hexb** | 5.87E-01 | 1.10E+00 | 4.27E-02 | 1.35E+00 | 7.36E-02 | 1.49E+00 |
| **Bdnf** | 5.95E-01 | 1.05E+00 | 9.85E-01 | 1.00E+00 | 6.23E-01 | 1.05E+00 |
| **Psmb9** | 5.95E-01 | 1.09E+00 | 3.07E-02 | 1.35E+00 | 7.25E-02 | 1.47E+00 |
| **Lox** | 6.00E-01 | 8.29E-01 | 5.20E-02 | 2.08E+00 | 2.74E-01 | 1.73E+00 |
| **Phf2** | 6.00E-01 | 1.02E+00 | 2.57E-01 | 9.16E-01 | 3.68E-01 | 9.35E-01 |
| **Epha3** | 6.03E-01 | 1.04E+00 | 5.59E-01 | 9.42E-01 | 8.06E-01 | 9.81E-01 |
| **Cd33** | 6.06E-01 | 1.15E+00 | 6.42E-01 | 1.07E+00 | 4.40E-01 | 1.23E+00 |
| **Gabrr3** | 6.06E-01 | 1.07E+00 | 9.67E-01 | 9.91E-01 | 7.29E-01 | 1.07E+00 |
| **Il10ra** | 6.06E-01 | 1.12E+00 | 2.04E-01 | 1.15E+00 | 3.08E-01 | 1.29E+00 |
| **Gss** | 6.06E-01 | 1.04E+00 | 5.30E-01 | 9.67E-01 | 9.62E-01 | 1.00E+00 |
| **Ppt1** | 6.06E-01 | 1.03E+00 | 3.07E-01 | 1.04E+00 | 2.22E-01 | 1.07E+00 |
| **Casp3** | 6.10E-01 | 1.10E+00 | 7.70E-02 | 1.28E+00 | 1.06E-01 | 1.41E+00 |
| **Notch3** | 6.10E-01 | 9.42E-01 | 2.07E-01 | 1.22E+00 | 2.77E-01 | 1.15E+00 |
| **Psen2** | 6.13E-01 | 1.02E+00 | 8.49E-01 | 9.90E-01 | 8.31E-01 | 1.01E+00 |
| **Ang** | 6.16E-01 | 9.27E-01 | 1.76E-01 | 1.29E+00 | 1.29E-01 | 1.20E+00 |
| **Taf4b** | 6.16E-01 | 9.62E-01 | 2.51E-01 | 1.06E+00 | 7.99E-01 | 1.02E+00 |
| **Drd2** | 6.16E-01 | 9.06E-01 | 7.91E-01 | 9.57E-01 | 3.65E-01 | 8.67E-01 |
| **Gucy1b3** | 6.21E-01 | 9.65E-01 | 7.85E-01 | 1.02E+00 | 7.71E-01 | 9.79E-01 |
| **Epha2** | 6.22E-01 | 1.08E+00 | 6.04E-01 | 9.20E-01 | 9.91E-01 | 9.98E-01 |
| **Erlec1** | 6.24E-01 | 1.02E+00 | 7.32E-02 | 1.07E+00 | 1.61E-01 | 1.09E+00 |
| **Lif** | 6.25E-01 | 1.11E+00 | 8.33E-01 | 9.66E-01 | 6.58E-01 | 1.07E+00 |
| **Sp1** | 6.30E-01 | 9.72E-01 | 8.38E-01 | 1.01E+00 | 8.47E-01 | 9.86E-01 |
| **Nrg1** | 6.32E-01 | 9.60E-01 | 1.20E-01 | 1.14E+00 | 3.57E-01 | 1.10E+00 |
| **Gba** | 6.39E-01 | 1.04E+00 | 8.87E-02 | 1.13E+00 | 9.07E-02 | 1.18E+00 |
| **Slc1a2** | 6.39E-01 | 1.06E+00 | 8.63E-01 | 1.02E+00 | 6.12E-01 | 1.08E+00 |
| **Mmp2** | 6.40E-01 | 9.27E-01 | 9.16E-03 | 1.59E+00 | 1.16E-01 | 1.48E+00 |
| **C1qa** | 6.44E-01 | 1.08E+00 | 7.40E-02 | 1.36E+00 | 7.02E-02 | 1.47E+00 |
| **Ngf** | 6.44E-01 | 9.59E-01 | 7.08E-01 | 1.04E+00 | 9.26E-01 | 9.93E-01 |
| **Calb2** | 6.45E-01 | 1.07E+00 | 3.79E-01 | 8.71E-01 | 5.26E-01 | 9.30E-01 |
| **Hdac1** | 6.51E-01 | 9.69E-01 | 9.33E-01 | 1.00E+00 | 7.47E-01 | 9.73E-01 |
| **Ide** | 6.56E-01 | 1.03E+00 | 7.93E-01 | 9.85E-01 | 8.28E-01 | 1.02E+00 |
| **Nr4a2** | 6.58E-01 | 1.04E+00 | 9.76E-01 | 9.97E-01 | 7.88E-01 | 1.04E+00 |
| **Dlx2** | 6.59E-01 | 1.06E+00 | 6.21E-01 | 9.12E-01 | 8.53E-01 | 9.67E-01 |
| **Pla2g2e** | 6.62E-01 | 1.12E+00 | 9.04E-01 | 9.74E-01 | 6.79E-01 | 1.09E+00 |
| **Lama2** | 6.63E-01 | 9.47E-01 | 1.03E-02 | 1.35E+00 | 1.58E-01 | 1.28E+00 |
| **Atp6v1e1** | 6.65E-01 | 1.02E+00 | 4.70E-01 | 1.03E+00 | 3.74E-01 | 1.05E+00 |
| **Itga5** | 6.66E-01 | 1.08E+00 | 3.04E-01 | 1.24E+00 | 1.66E-01 | 1.34E+00 |
| **Lmna** | 6.71E-01 | 1.02E+00 | 9.38E-01 | 1.00E+00 | 6.96E-01 | 1.02E+00 |
| **Pgam1** | 6.72E-01 | 9.90E-01 | 9.80E-01 | 9.99E-01 | 7.75E-01 | 9.89E-01 |
| **Il1b** | 6.73E-01 | 8.92E-01 | 4.66E-01 | 1.33E+00 | 6.32E-01 | 1.19E+00 |
| **Gria4** | 6.73E-01 | 1.03E+00 | 2.67E-01 | 1.08E+00 | 1.06E-01 | 1.11E+00 |
| **Nol3** | 6.75E-01 | 1.05E+00 | 6.91E-01 | 1.05E+00 | 4.76E-01 | 1.10E+00 |
| **Cln3** | 6.76E-01 | 1.04E+00 | 5.88E-01 | 1.05E+00 | 2.36E-01 | 1.10E+00 |
| **Lrrc25** | 6.76E-01 | 9.42E-01 | 3.77E-02 | 1.46E+00 | 6.54E-02 | 1.38E+00 |
| **Pls1** | 6.78E-01 | 1.03E+00 | 7.03E-01 | 1.06E+00 | 5.55E-01 | 1.09E+00 |
| **Sgpl1** | 6.79E-01 | 1.03E+00 | 9.13E-02 | 1.12E+00 | 1.26E-01 | 1.16E+00 |
| **Tnf** | 6.79E-01 | 8.66E-01 | 3.37E-01 | 1.57E+00 | 4.41E-01 | 1.36E+00 |
| **Taf9** | 6.80E-01 | 1.03E+00 | 4.23E-01 | 9.68E-01 | 9.08E-01 | 9.93E-01 |
| **Ntng1** | 6.81E-01 | 9.72E-01 | 6.88E-01 | 9.79E-01 | 4.34E-01 | 9.51E-01 |
| **Ap1s1** | 6.82E-01 | 1.05E+00 | 8.38E-01 | 9.89E-01 | 7.29E-01 | 1.04E+00 |
| **Prkaca** | 6.83E-01 | 9.83E-01 | 8.72E-01 | 9.92E-01 | 5.89E-01 | 9.75E-01 |
| **Ccnh** | 6.85E-01 | 1.02E+00 | 8.16E-01 | 1.01E+00 | 5.72E-01 | 1.03E+00 |
| **Syt13** | 6.86E-01 | 9.84E-01 | 8.84E-01 | 9.92E-01 | 6.82E-01 | 9.76E-01 |
| **Itpr2** | 6.87E-01 | 9.48E-01 | 1.96E-01 | 1.21E+00 | 4.04E-01 | 1.15E+00 |
| **Efna1** | 6.88E-01 | 9.66E-01 | 2.14E-01 | 1.10E+00 | 5.43E-01 | 1.06E+00 |
| **Cldn5** | 6.92E-01 | 9.44E-01 | 6.53E-02 | 1.27E+00 | 2.89E-01 | 1.20E+00 |
| **Atxn3** | 6.92E-01 | 9.60E-01 | 8.21E-01 | 1.02E+00 | 7.84E-01 | 9.77E-01 |
| **Ddx23** | 6.93E-01 | 9.89E-01 | 5.50E-01 | 9.75E-01 | 4.27E-01 | 9.64E-01 |
| **Lpo** | 6.99E-01 | 1.10E+00 | 7.27E-01 | 1.09E+00 | 4.57E-01 | 1.19E+00 |
| **Dnah1** | 7.01E-01 | 1.07E+00 | 7.22E-01 | 9.42E-01 | 9.72E-01 | 1.01E+00 |
| **Sorcs3** | 7.02E-01 | 9.57E-01 | 1.97E-01 | 1.09E+00 | 7.32E-01 | 1.04E+00 |
| **Parp1** | 7.03E-01 | 9.67E-01 | 3.33E-01 | 9.37E-01 | 2.57E-01 | 9.06E-01 |
| **Gabrp** | 7.03E-01 | 1.07E+00 | 5.77E-01 | 7.87E-01 | 6.89E-01 | 8.41E-01 |
| **Cast** | 7.04E-01 | 1.06E+00 | 4.11E-03 | 1.42E+00 | 4.02E-02 | 1.51E+00 |
| **Kcnb1** | 7.07E-01 | 9.75E-01 | 6.30E-01 | 9.76E-01 | 4.36E-01 | 9.52E-01 |
| **Cd68** | 7.08E-01 | 9.09E-01 | 2.27E-02 | 1.78E+00 | 1.10E-01 | 1.62E+00 |
| **Cxcl12** | 7.09E-01 | 9.41E-01 | 4.37E-01 | 1.07E+00 | 9.82E-01 | 1.00E+00 |
| **Grm2** | 7.15E-01 | 1.03E+00 | 1.33E-02 | 8.28E-01 | 1.16E-02 | 8.51E-01 |
| **Igf1** | 7.15E-01 | 9.63E-01 | 1.54E-01 | 1.18E+00 | 9.43E-02 | 1.14E+00 |
| **Cxcr4** | 7.18E-01 | 1.07E+00 | 2.05E-02 | 1.33E+00 | 1.14E-01 | 1.42E+00 |
| **Ptdss1** | 7.19E-01 | 9.87E-01 | 3.46E-02 | 1.06E+00 | 2.52E-01 | 1.05E+00 |
| **Ninj2** | 7.23E-01 | 9.64E-01 | 5.66E-02 | 1.27E+00 | 1.07E-01 | 1.23E+00 |
| **Sox10** | 7.27E-01 | 9.80E-01 | 9.62E-01 | 9.93E-01 | 8.53E-01 | 9.73E-01 |
| **Itpr1** | 7.30E-01 | 9.69E-01 | 7.36E-01 | 9.83E-01 | 6.07E-01 | 9.52E-01 |
| **Nes** | 7.31E-01 | 9.62E-01 | 2.56E-02 | 1.35E+00 | 7.20E-02 | 1.30E+00 |
| **Cdk2** | 7.32E-01 | 1.07E+00 | 7.21E-01 | 1.06E+00 | 5.07E-01 | 1.14E+00 |
| **Ikbkb** | 7.34E-01 | 9.87E-01 | 4.09E-01 | 9.73E-01 | 3.08E-01 | 9.60E-01 |
| **Ccl12** | 7.34E-01 | 8.80E-01 | 1.07E-01 | 2.60E+00 | 1.63E-02 | 2.29E+00 |
| **Casp9** | 7.35E-01 | 9.73E-01 | 6.95E-01 | 9.74E-01 | 5.59E-01 | 9.47E-01 |
| **Hmox1** | 7.36E-01 | 1.04E+00 | 1.43E-01 | 1.13E+00 | 2.15E-01 | 1.18E+00 |
| **Optn** | 7.40E-01 | 9.87E-01 | 2.67E-01 | 1.06E+00 | 4.03E-01 | 1.05E+00 |
| **Trpv1** | 7.41E-01 | 1.16E+00 | 6.69E-01 | 1.16E+00 | 4.93E-01 | 1.35E+00 |
| **Gabrr1** | 7.42E-01 | 8.97E-01 | 7.74E-01 | 1.09E+00 | 9.60E-01 | 9.78E-01 |
| **Bcl2** | 7.43E-01 | 1.04E+00 | 7.86E-01 | 1.03E+00 | 6.00E-01 | 1.07E+00 |
| **Pah** | 7.43E-01 | 9.44E-01 | 1.02E-01 | 1.55E+00 | 2.29E-01 | 1.46E+00 |
| **Spast** | 7.51E-01 | 9.87E-01 | 7.04E-01 | 9.87E-01 | 3.39E-01 | 9.74E-01 |
| **Pla2g6** | 7.53E-01 | 1.02E+00 | 6.54E-01 | 9.64E-01 | 8.00E-01 | 9.82E-01 |
| **Arsa** | 7.53E-01 | 1.02E+00 | 1.74E-01 | 1.06E+00 | 2.23E-01 | 1.08E+00 |
| **Tbpl1** | 7.54E-01 | 1.01E+00 | 5.74E-01 | 9.79E-01 | 7.76E-01 | 9.90E-01 |
| **Mag** | 7.56E-01 | 9.73E-01 | 9.14E-01 | 9.86E-01 | 6.96E-01 | 9.60E-01 |
| **Itpr3** | 7.56E-01 | 9.54E-01 | 5.16E-01 | 1.11E+00 | 7.09E-01 | 1.06E+00 |
| **Creb1** | 7.59E-01 | 1.02E+00 | 1.19E-01 | 1.07E+00 | 1.72E-01 | 1.09E+00 |
| **Raf1** | 7.62E-01 | 9.83E-01 | 9.59E-01 | 9.97E-01 | 7.37E-01 | 9.80E-01 |
| **Sec23a** | 7.62E-01 | 1.01E+00 | 9.93E-01 | 1.00E+00 | 8.01E-01 | 1.01E+00 |
| **Mpz** | 7.63E-01 | 1.06E+00 | 3.27E-01 | 7.67E-01 | 4.74E-01 | 8.15E-01 |
| **Ryr1** | 7.63E-01 | 1.06E+00 | 3.15E-02 | 6.67E-01 | 6.12E-02 | 7.04E-01 |
| **Dld** | 7.68E-01 | 1.01E+00 | 1.42E-01 | 1.03E+00 | 1.53E-01 | 1.04E+00 |
| **Ntf3** | 7.71E-01 | 9.55E-01 | 6.35E-02 | 8.37E-01 | 1.60E-01 | 7.99E-01 |
| **Lrrk2** | 7.73E-01 | 9.71E-01 | 4.03E-02 | 1.15E+00 | 3.23E-01 | 1.12E+00 |
| **Csf1** | 7.83E-01 | 1.02E+00 | 6.75E-01 | 1.03E+00 | 5.41E-01 | 1.05E+00 |
| **Mecp2** | 7.85E-01 | 9.82E-01 | 6.84E-01 | 9.76E-01 | 5.57E-01 | 9.59E-01 |
| **Fgf2** | 7.91E-01 | 1.03E+00 | 1.13E-01 | 1.24E+00 | 7.76E-02 | 1.28E+00 |
| **Eng** | 7.91E-01 | 9.66E-01 | 2.65E-02 | 1.37E+00 | 4.89E-02 | 1.32E+00 |
| **Slc6a4** | 7.94E-01 | 1.09E+00 | 4.62E-01 | 1.36E+00 | 1.37E-01 | 1.48E+00 |
| **Mutyh** | 7.95E-01 | 9.42E-01 | 9.56E-01 | 9.93E-01 | 7.45E-01 | 9.36E-01 |
| **Gtf2h1** | 7.96E-01 | 1.02E+00 | 7.93E-01 | 1.01E+00 | 6.72E-01 | 1.02E+00 |
| **Ctse** | 7.98E-01 | 9.43E-01 | 1.38E-01 | 1.37E+00 | 2.61E-01 | 1.29E+00 |
| **Cd44** | 8.00E-01 | 1.03E+00 | 2.73E-02 | 1.38E+00 | 6.01E-02 | 1.42E+00 |
| **Nkx6-2** | 8.05E-01 | 9.60E-01 | 9.56E-01 | 1.01E+00 | 8.69E-01 | 9.71E-01 |
| **Hgf** | 8.09E-01 | 9.66E-01 | 2.24E-01 | 1.18E+00 | 2.76E-01 | 1.14E+00 |
| **Prkcsh** | 8.09E-01 | 1.01E+00 | 3.70E-01 | 1.05E+00 | 2.92E-01 | 1.06E+00 |
| **Gga1** | 8.13E-01 | 1.01E+00 | 4.95E-01 | 1.02E+00 | 4.39E-01 | 1.03E+00 |
| **Brms1l** | 8.16E-01 | 1.01E+00 | 5.55E-02 | 9.29E-01 | 1.72E-01 | 9.40E-01 |
| **Ret** | 8.17E-01 | 1.06E+00 | 8.82E-01 | 9.50E-01 | 9.85E-01 | 1.01E+00 |
| **Ngfr** | 8.18E-01 | 9.37E-01 | 2.67E-01 | 1.28E+00 | 5.33E-01 | 1.20E+00 |
| **Stx1b** | 8.21E-01 | 1.00E+00 | 4.43E-01 | 9.72E-01 | 5.07E-01 | 9.77E-01 |
| **Gad2** | 8.21E-01 | 1.02E+00 | 4.55E-01 | 9.09E-01 | 4.91E-01 | 9.29E-01 |
| **Tnfrsf11b** | 8.21E-01 | 1.05E+00 | 5.21E-01 | 1.16E+00 | 3.23E-01 | 1.22E+00 |
| **Shank2** | 8.24E-01 | 9.78E-01 | 8.64E-01 | 9.79E-01 | 7.00E-01 | 9.58E-01 |
| **Adra2a** | 8.26E-01 | 1.02E+00 | 3.57E-01 | 9.24E-01 | 3.03E-01 | 9.40E-01 |
| **Smpd4** | 8.28E-01 | 1.03E+00 | 6.34E-01 | 9.57E-01 | 8.34E-01 | 9.81E-01 |
| **Dbh** | 8.29E-01 | 9.65E-01 | 4.65E-02 | 1.40E+00 | 2.04E-01 | 1.35E+00 |
| **Mta2** | 8.33E-01 | 1.01E+00 | 7.29E-01 | 9.83E-01 | 8.56E-01 | 9.91E-01 |
| **Gtf2h3** | 8.36E-01 | 1.01E+00 | 4.67E-01 | 9.67E-01 | 7.35E-01 | 9.79E-01 |
| **Axin2** | 8.39E-01 | 1.02E+00 | 4.39E-01 | 9.19E-01 | 5.96E-01 | 9.40E-01 |
| **Kras** | 8.39E-01 | 9.93E-01 | 8.06E-01 | 9.91E-01 | 5.95E-01 | 9.84E-01 |
| **Csf1r** | 8.42E-01 | 1.03E+00 | 1.00E-01 | 1.25E+00 | 1.52E-01 | 1.29E+00 |
| **P2rx7** | 8.42E-01 | 1.02E+00 | 6.57E-01 | 9.54E-01 | 8.69E-01 | 9.77E-01 |
| **Hdac2** | 8.44E-01 | 9.90E-01 | 9.90E-01 | 1.00E+00 | 8.90E-01 | 9.91E-01 |
| **Tgfbr2** | 8.50E-01 | 9.82E-01 | 5.15E-03 | 1.54E+00 | 2.59E-03 | 1.51E+00 |
| **Tnc** | 8.52E-01 | 9.69E-01 | 1.18E-01 | 1.30E+00 | 1.88E-01 | 1.26E+00 |
| **Mapk3** | 8.53E-01 | 1.01E+00 | 7.14E-01 | 9.80E-01 | 7.97E-01 | 9.88E-01 |
| **Cdk5rap3** | 8.54E-01 | 1.02E+00 | 5.53E-01 | 9.65E-01 | 8.55E-01 | 9.83E-01 |
| **Sncb** | 8.54E-01 | 1.01E+00 | 7.61E-01 | 9.73E-01 | 8.67E-01 | 9.83E-01 |
| **Spi1** | 8.55E-01 | 1.05E+00 | 3.40E-01 | 1.18E+00 | 4.03E-01 | 1.23E+00 |
| **Crtc2** | 8.57E-01 | 1.02E+00 | 3.78E-01 | 9.18E-01 | 2.98E-01 | 9.35E-01 |
| **Nwd1** | 8.58E-01 | 1.02E+00 | 8.24E-01 | 1.03E+00 | 7.59E-01 | 1.05E+00 |
| **Gngt1** | 8.60E-01 | 1.04E+00 | 4.11E-01 | 8.08E-01 | 3.96E-01 | 8.38E-01 |
| **Gdnf** | 8.62E-01 | 1.06E+00 | 7.55E-02 | 6.14E-01 | 2.35E-01 | 6.50E-01 |
| **Ggt1** | 8.63E-01 | 9.77E-01 | 8.58E-02 | 1.33E+00 | 1.01E-01 | 1.30E+00 |
| **Hpgds** | 8.63E-01 | 1.05E+00 | 3.55E-01 | 1.26E+00 | 3.48E-01 | 1.33E+00 |
| **Cul2** | 8.64E-01 | 9.94E-01 | 3.33E-02 | 1.06E+00 | 1.60E-01 | 1.05E+00 |
| **C6** | 8.67E-01 | 9.52E-01 | 5.96E-01 | 1.14E+00 | 7.13E-01 | 1.08E+00 |
| **Mmrn2** | 8.67E-01 | 9.73E-01 | 1.23E-01 | 1.21E+00 | 3.00E-01 | 1.18E+00 |
| **Nfkbib** | 8.73E-01 | 1.01E+00 | 5.14E-01 | 9.60E-01 | 7.43E-01 | 9.72E-01 |
| **Tnfrsf10b** | 8.75E-01 | 9.66E-01 | 2.10E-02 | 1.45E+00 | 1.84E-01 | 1.40E+00 |
| **Itgam** | 8.77E-01 | 9.75E-01 | 1.04E-01 | 1.22E+00 | 3.44E-01 | 1.19E+00 |
| **Vegfa** | 8.80E-01 | 1.01E+00 | 9.94E-02 | 9.09E-01 | 3.54E-01 | 9.21E-01 |
| **Adcy5** | 8.91E-01 | 9.81E-01 | 9.68E-01 | 9.95E-01 | 8.84E-01 | 9.76E-01 |
| **Itgax** | 8.93E-01 | 1.04E+00 | 1.82E-01 | 1.55E+00 | 8.90E-03 | 1.61E+00 |
| **Ntrk1** | 8.94E-01 | 9.81E-01 | 5.62E-01 | 8.53E-01 | 5.27E-01 | 8.36E-01 |
| **Cers1** | 8.98E-01 | 9.87E-01 | 1.48E-01 | 8.61E-01 | 1.27E-01 | 8.49E-01 |
| **Taf6l** | 8.98E-01 | 9.89E-01 | 3.54E-01 | 1.08E+00 | 1.97E-01 | 1.07E+00 |
| **Pla2g4f** | 9.00E-01 | 9.65E-01 | 4.01E-01 | 8.07E-01 | 2.83E-01 | 7.79E-01 |
| **Sf3b4** | 9.01E-01 | 1.02E+00 | 2.35E-01 | 8.83E-01 | 4.27E-01 | 8.98E-01 |
| **Cpt1b** | 9.02E-01 | 9.63E-01 | 4.22E-01 | 1.27E+00 | 6.11E-01 | 1.22E+00 |
| **Erg** | 9.02E-01 | 1.03E+00 | 2.49E-01 | 1.23E+00 | 4.43E-01 | 1.27E+00 |
| **Atp7a** | 9.03E-01 | 1.02E+00 | 1.56E-02 | 1.36E+00 | 1.21E-01 | 1.39E+00 |
| **Casp7** | 9.04E-01 | 9.83E-01 | 1.31E-01 | 1.16E+00 | 3.25E-01 | 1.14E+00 |
| **Th** | 9.04E-01 | 1.03E+00 | 8.21E-01 | 1.06E+00 | 7.77E-01 | 1.10E+00 |
| **Il15ra** | 9.07E-01 | 1.02E+00 | 5.15E-01 | 9.48E-01 | 8.51E-01 | 9.69E-01 |
| **Apc** | 9.07E-01 | 9.87E-01 | 8.46E-01 | 1.02E+00 | 9.49E-01 | 1.01E+00 |
| **Tgfb1** | 9.08E-01 | 1.02E+00 | 4.90E-02 | 1.38E+00 | 8.21E-02 | 1.41E+00 |
| **Vip** | 9.09E-01 | 1.02E+00 | 3.82E-01 | 1.13E+00 | 1.97E-01 | 1.15E+00 |
| **Olfm3** | 9.10E-01 | 1.01E+00 | 6.59E-01 | 9.60E-01 | 6.84E-01 | 9.67E-01 |
| **Ldhc** | 9.11E-01 | 9.73E-01 | 8.60E-01 | 1.07E+00 | 9.12E-01 | 1.04E+00 |
| **Keap1** | 9.14E-01 | 9.94E-01 | 5.87E-01 | 9.70E-01 | 3.88E-01 | 9.64E-01 |
| **Srsf4** | 9.18E-01 | 9.97E-01 | 2.19E-01 | 9.46E-01 | 2.21E-01 | 9.43E-01 |
| **Src** | 9.19E-01 | 1.00E+00 | 4.26E-02 | 9.25E-01 | 2.12E-02 | 9.27E-01 |
| **Cd34** | 9.21E-01 | 9.91E-01 | 8.55E-01 | 1.01E+00 | 9.61E-01 | 1.01E+00 |
| **Lamb2** | 9.22E-01 | 1.01E+00 | 1.58E-02 | 1.47E+00 | 3.98E-02 | 1.49E+00 |
| **Sp100** | 9.24E-01 | 1.01E+00 | 6.77E-02 | 1.37E+00 | 7.46E-02 | 1.39E+00 |
| **Grn** | 9.24E-01 | 1.01E+00 | 1.89E-02 | 1.30E+00 | 2.31E-02 | 1.32E+00 |
| **Epha5** | 9.26E-01 | 1.00E+00 | 2.62E-01 | 1.04E+00 | 4.59E-01 | 1.04E+00 |
| **Des** | 9.26E-01 | 9.79E-01 | 8.98E-02 | 1.41E+00 | 1.94E-01 | 1.38E+00 |
| **Ptdss2** | 9.35E-01 | 1.01E+00 | 3.02E-02 | 1.06E+00 | 4.39E-01 | 1.07E+00 |
| **Trim37** | 9.38E-01 | 9.98E-01 | 4.33E-01 | 9.73E-01 | 4.45E-01 | 9.71E-01 |
| **Plcb2** | 9.40E-01 | 9.85E-01 | 9.21E-01 | 1.02E+00 | 9.92E-01 | 1.00E+00 |
| **Pkn1** | 9.41E-01 | 9.95E-01 | 6.83E-01 | 9.58E-01 | 6.48E-01 | 9.52E-01 |
| **Il4ra** | 9.43E-01 | 1.01E+00 | 2.00E-01 | 1.19E+00 | 2.94E-01 | 1.20E+00 |
| **Csf2rb** | 9.48E-01 | 9.84E-01 | 4.82E-01 | 1.19E+00 | 4.04E-01 | 1.17E+00 |
| **Slc32a1** | 9.50E-01 | 9.92E-01 | 9.19E-01 | 9.84E-01 | 8.56E-01 | 9.77E-01 |
| **Pla2g2a** | 9.52E-01 | 1.03E+00 | 6.47E-01 | 1.18E+00 | 5.78E-01 | 1.21E+00 |
| **Snca** | 9.54E-01 | 9.97E-01 | 9.64E-03 | 1.08E+00 | 1.99E-01 | 1.08E+00 |
| **Irf8** | 9.55E-01 | 1.01E+00 | 7.61E-02 | 1.44E+00 | 7.06E-02 | 1.45E+00 |
| **Tnfrsf1b** | 9.64E-01 | 9.94E-01 | 2.31E-01 | 1.18E+00 | 1.51E-01 | 1.17E+00 |
| **Cldn15** | 9.64E-01 | 9.90E-01 | 2.75E-01 | 1.18E+00 | 5.01E-01 | 1.17E+00 |
| **Grin2d** | 9.64E-01 | 9.95E-01 | 8.44E-01 | 9.79E-01 | 8.03E-01 | 9.75E-01 |
| **Notch4** | 9.66E-01 | 9.94E-01 | 6.50E-01 | 1.09E+00 | 6.71E-01 | 1.09E+00 |
| **Xab2** | 9.68E-01 | 9.98E-01 | 9.49E-01 | 9.99E-01 | 9.44E-01 | 9.97E-01 |
| **Gtf2a1** | 9.70E-01 | 9.99E-01 | 8.59E-01 | 9.94E-01 | 8.62E-01 | 9.93E-01 |
| **Adcy8** | 9.72E-01 | 1.00E+00 | 4.54E-01 | 1.09E+00 | 4.06E-01 | 1.09E+00 |
| **Pla2g4c** | 9.72E-01 | 1.01E+00 | 5.00E-02 | 1.52E+00 | 4.06E-02 | 1.53E+00 |
| **Nefh** | 9.74E-01 | 1.00E+00 | 5.21E-01 | 1.03E+00 | 5.76E-01 | 1.03E+00 |
| **Klk6** | 9.78E-01 | 1.00E+00 | 8.46E-01 | 1.03E+00 | 8.59E-01 | 1.04E+00 |
| **Rab2a** | 9.78E-01 | 1.00E+00 | 3.59E-01 | 9.70E-01 | 4.87E-01 | 9.71E-01 |
| **Il1r1** | 9.79E-01 | 1.00E+00 | 2.42E-01 | 8.81E-01 | 4.14E-01 | 8.84E-01 |
| **Pgk1** | 9.80E-01 | 1.00E+00 | 5.32E-01 | 1.03E+00 | 6.14E-01 | 1.03E+00 |
| **Trf** | 9.81E-01 | 9.99E-01 | 3.93E-01 | 1.12E+00 | 3.48E-01 | 1.12E+00 |
| **U2af2** | 9.81E-01 | 1.00E+00 | 6.08E-01 | 1.03E+00 | 7.18E-01 | 1.03E+00 |
| **Cdk7** | 9.83E-01 | 9.99E-01 | 9.12E-01 | 9.95E-01 | 9.16E-01 | 9.94E-01 |
| **Map2** | 9.85E-01 | 1.00E+00 | 9.95E-01 | 1.00E+00 | 9.82E-01 | 1.00E+00 |
| **Akt1** | 9.87E-01 | 9.99E-01 | 2.25E-01 | 9.32E-01 | 2.14E-01 | 9.31E-01 |
| **Egfr** | 9.90E-01 | 1.00E+00 | 5.40E-01 | 1.08E+00 | 5.59E-01 | 1.08E+00 |
| **Cxcl10** | 9.95E-01 | 1.00E+00 | 8.44E-02 | 2.40E+00 | 1.12E-01 | 2.40E+00 |
| **Efr3a** | 9.96E-01 | 1.00E+00 | 1.67E-01 | 1.05E+00 | 3.69E-01 | 1.05E+00 |
| **Pde1b** | 9.96E-01 | 1.00E+00 | 3.23E-02 | 8.97E-01 | 6.03E-02 | 8.97E-01 |
